# Dynamic Control of the Self-Assembling Properties of Cyclodextrins by the Interplay of Aromatic and Host-Guest Interactions

**DOI:** 10.3389/fchem.2019.00072

**Published:** 2019-02-25

**Authors:** Tania Neva, Thais Carmona, Juan M. Benito, Cédric Przybylski, Carmen Ortiz Mellet, Francisco Mendicuti, José M. García Fernández

**Affiliations:** ^1^Instituto de Investigaciones Químicas (IIQ), CSIC - University of Sevilla, Sevilla, Spain; ^2^Department of Analytical Chemistry, Physical Chemistry and Chemical Engineering, Faculty of Chemistry, University of Alcalá, Alcalá de Henares, Madrid, Spain; ^3^Sorbonne Université, CNRS, Institut Parisien de Chimie Moléculaire, IPCM, Paris, France; ^4^Department of Organic Chemistry, Faculty of Chemistry, University of Seville, Seville, Spain

**Keywords:** cyclodextrins, self-assembly, supramolecular chemistry, naphthalene, host-guest chemistry, fluorescence, circular dichroism

## Abstract

The presence of a doubly-linked naphthylene clip at the *O*-2^I^ and *O*-3^II^ positions in the secondary ring of β-cyclodextrin (βCD) derivatives promoted their self-assembly into head-to-head supramolecular dimers in which the aromatic modules act either as cavity extension walls (if the naphthalene moiety is 1,8-disubstituted) or as folding screens that separate the individual βCD units (if 2,3-disubstituted). Dimer architecture is governed by the conformational properties of the monomer constituents, as determined by NMR, fluorescence, circular dichroism, and computational techniques. In a second supramolecular organization level, the topology of the assembly directs host-guest interactions and, reciprocally, guest inclusion impacts the stability of the supramolecular edifice. Thus, inclusion of adamantane carboxylate, a well-known βCD cavity-fitting guest, was found to either preserve the dimeric arrangement, leading to multicomponent species, or elicit dimer disruption. The ensemble of results highlights the potential of the approach to program self-organization and external stimuli responsiveness of CD devices in a controlled manner while keeping full diastereomeric purity.

## Introduction

The incorporation of functional moieties into pre-existing cage molecules and macrocycles represents a powerful strategy to tailor their architectural, inclusion, self-assembling, stimulus responsiveness and biorecognition properties (Sansone and Casnati, 2013; Shetty et al., 2015; Gropp et al., 2017; Liu et al., 2017; Matsuoka and Nabeshima, 2018). A range of sophisticated molecular machines with programmed supramolecular behaviors have been designed by implementing this strategy, with applications in the fields of sensors, electronic or optical devices, natural ligand mimics, artificial catalysts or (bio) molecular carriers, among others (Jiménez Blanco et al., 2017; Webber and Langer, 2017; Yin et al., 2017; Guo et al., 2018; van Dijk et al., 2018; Yu et al., 2018; Zeng et al., 2018). The natural cyclooligosaccharides of the cyclodextrin family are paradigmatic examples in this context (Crini, 2014). The circular arrangement of α-(1 → 4)-linked D-glucopyranosyl units in the commercially available hexa-, hepta-, and octameric representatives (α, β, and γCD, respectively) draws a hollow truncated-cone frame with a hydrophobic interior that can host complementary guests in a size-conditional basis (Simões et al., 2015; Ryzhakov et al., 2016; Álvarez-Lorenzo et al., 2017; García-Moreno et al., 2017; Carreño et al., 2018). The volume constraint can be overcome by the incorporation of shaping and recognition modules leading to cavity extension, cooperativity effects or self-assembly, allowing several levels of organization to be implemented. The design of non-viral gene delivery systems (Ortiz Mellet et al., 2011; Gallego-Yerga et al., 2015; Evenou et al., 2018; Hong et al., 2018), antitoxins (Díaz-Moscoso et al., 2011; Joshi et al., 2011), nanocontainers (Gallego-Yerga et al., 2014b, 2017; Varan et al., 2017; Engel et al., 2018) and actuators (Smiljanic et al., 2006; Tan et al., 2014; Yang et al., 2014) highlights the potential of this tactic.

**Figure 1:**
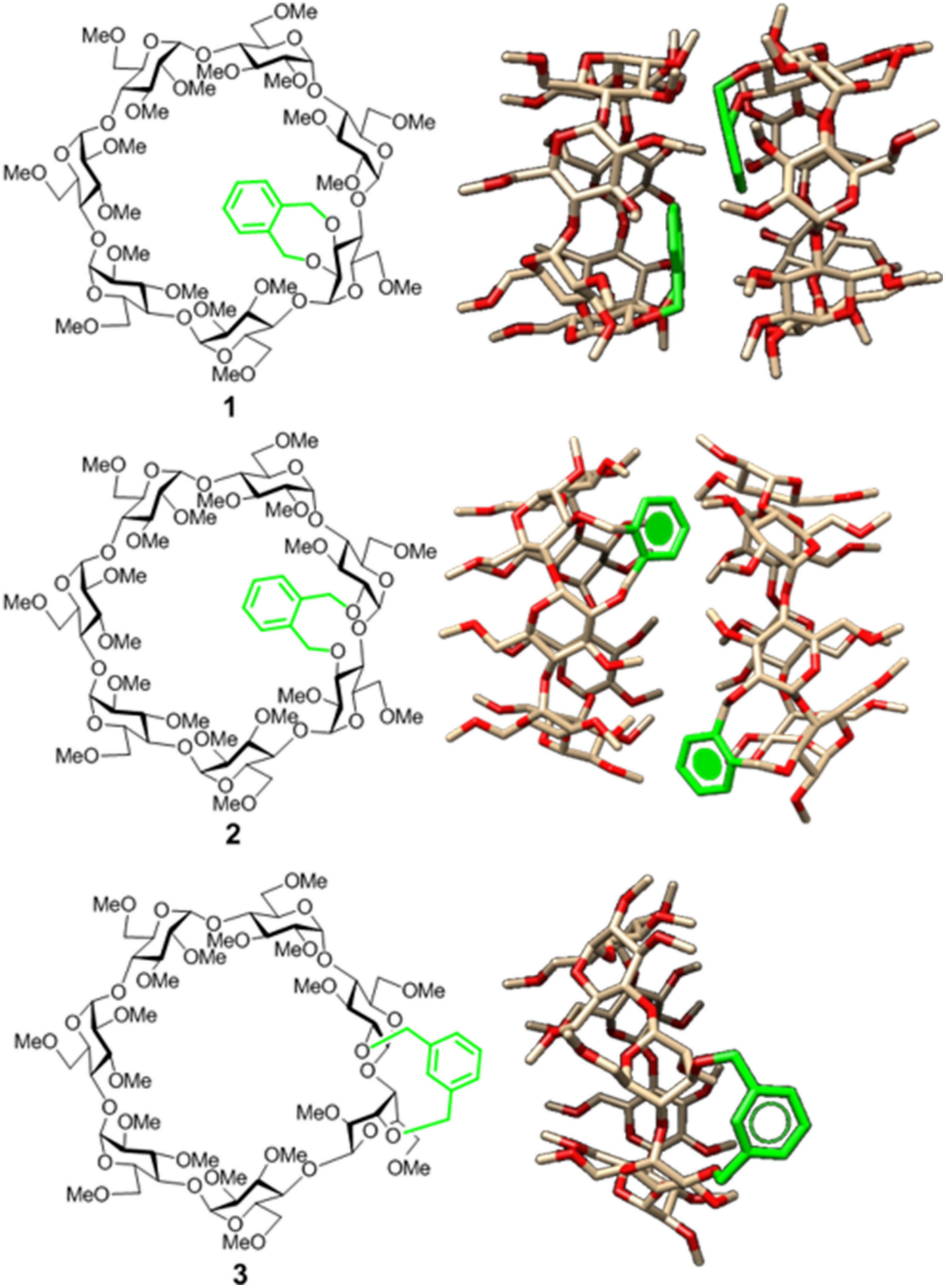
Structures of the doubly-linked isomeric βCD-xylylene conjugates **1**–**3 (Left)** and 3D molecular models of the corresponding minima binding energy dimer (for **1** and **2**) or monomer species (for **3**) present in water solution **(Right)**. Note that the aromatic ring (in green) orientation, relative to the CD cavity, shifts from cap-like to semi-open to fully open on moving from **1** to **3** (González-Álvarez et al., 2013; Neva et al., 2018).

**Figure 2:**
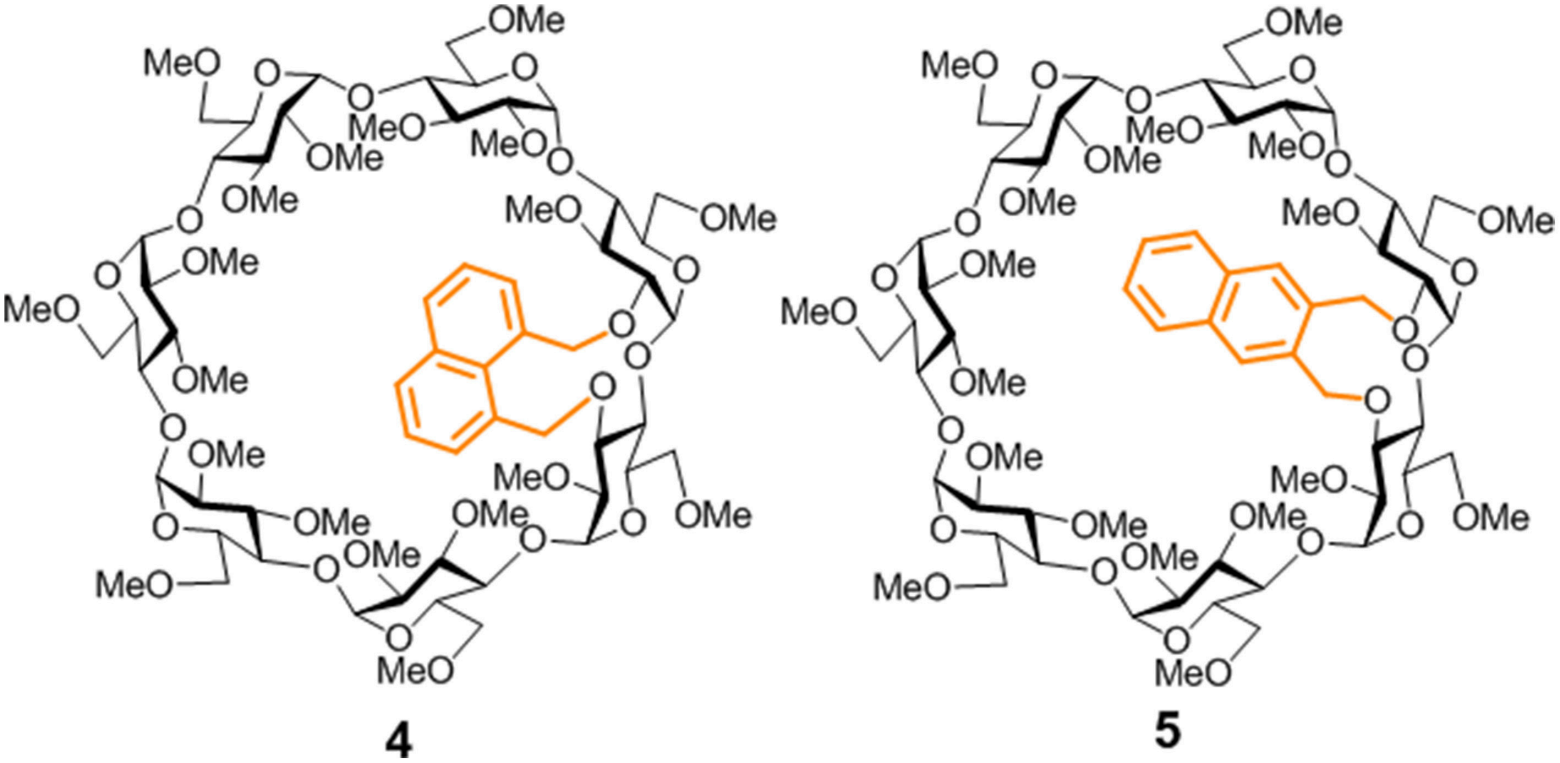
Structures of the new βCD conjugates equipped with naphthalene clips prepared in this work.

Appending aromatic walls at the CD rims has proven particularly beneficial to promote self-association and reinforce interactions with third species in a predictable manner (Benkovics et al., 2017; Zhu et al., 2017; Zhang et al., 2018), eventually enabling additional non-inclusion or interfacial complexing modes (McNally et al., 2009; de Jesus et al., 2012). Most reported examples of CD-aromatic conjugates focus on single position-linked derivatives that keep considerable mobility, which limits accurate three-dimensional definition, however (Gamieldien et al., 2010). Covalently bridging CD building blocks with aromatic tethers has been advantageously exploited for preorganization purposes in some cases (Carmona et al., 2015; Sun et al., 2015; Gallego-Yerga et al., 2018). Alternatively, prototypes featuring a two-point connected aromatic component at either the primary (Yang et al., 2008; Le Gac et al., 2016; Ménand et al., 2016, 2018; Yan et al., 2017) or the secondary rim (Balbuena et al., 2007, 2013) in a monomeric CD derivative have been reported, allowing unprecedented control over the topological and supramolecular attributes. The later architecture is particularly appealing: an aromatic component attached to the wider entrance of the inner cavity can act as gatekeeper, guest selector and/or aggregation promoter, the impact in the recognition abilities depending on the conformational bias (González-Álvarez et al., 2011, 2013). Thus, “hinge-type” *o*-xylylene segments connecting the vicinal secondary *O*-2 and *O*-3 positions in the same glucopyranosyl unit (e.g., **1**) adopt a cap-like orientation that hinders inclusion and elicits instead the formation of supramolecular head-to-head (HH) dimers in which the individual constituents are closely packed (González-Álvarez et al., 2008; Mayordomo et al., 2013; Gallego-Yerga et al., 2014a). Differently, “clip-type” positional isomers in which the *o*-xylylene group intertwines the *O*-2^I^ and *O*-3^II^ positions in consecutive monosaccharide units (e.g., **2**) preferentially take semi-open arrangements due to the reduced flexibility at the cyclic benzylic area. As a result, dimeric species with higher interfacial distances that readily dissociate in the presence of a suitable CD cavity-fitting guest are formed, which can be exploited for the spatiotemporal control of the recognition properties (Neva et al., 2018). Interestingly, the supramolecular abilities were drastically influenced by the type of joint between the aromatic and the cyclooligosaccaride parts: replacing the *o*-xylylene into a *m*-xylylene moiety in the clip-type geometry (e.g., **3**) led to a fully open topology that abolished self-recognition and favored instead non-inclusion complexation of planar guests (Figure 1). It was anticipated that non-covalent interactions in this family of CD conjugates could be further tuned by modifying the nature of the aromatic clip, providing a flexible scheme to dynamically mold different self-assembling and host-guest fitting modes. The incorporation of naphthalene modules is particularly appealing at this respect: several relative orientations between the cyclooligosaccharide macroring and the aromatic surface can be preselected as a function of the naphthalene substitution pattern without altering the structure and inclusion capabilities of the CD cavity. We conceived that this concept could be implemented in the design of monomeric prototypes with the ability to form supramolecular dimers in which the CD cavities can be either interconnected, forming a single compartment, or separated by the aromatic walls. To probe this hypothesis, the new 1,8-disubstituted or 2,3-disubstituted naphthylene-equipped βCD isomers **4** and **5** (Figure 2) have been synthesized and their conformational, self-assembling and inclusion properties have been monitored by fluorescence spectroscopy, circular dichroism, NMR, MS, and computational techniques. The results underline the potential of strategies based on precise synthesis to drive aggregation of macromolecular entities and program their responsiveness to molecular recognition events, a critical aspect in the construction of functional devices.

## Experimental and Computational Methods

### Synthesis and Compound Characterization

**Figure 3:**
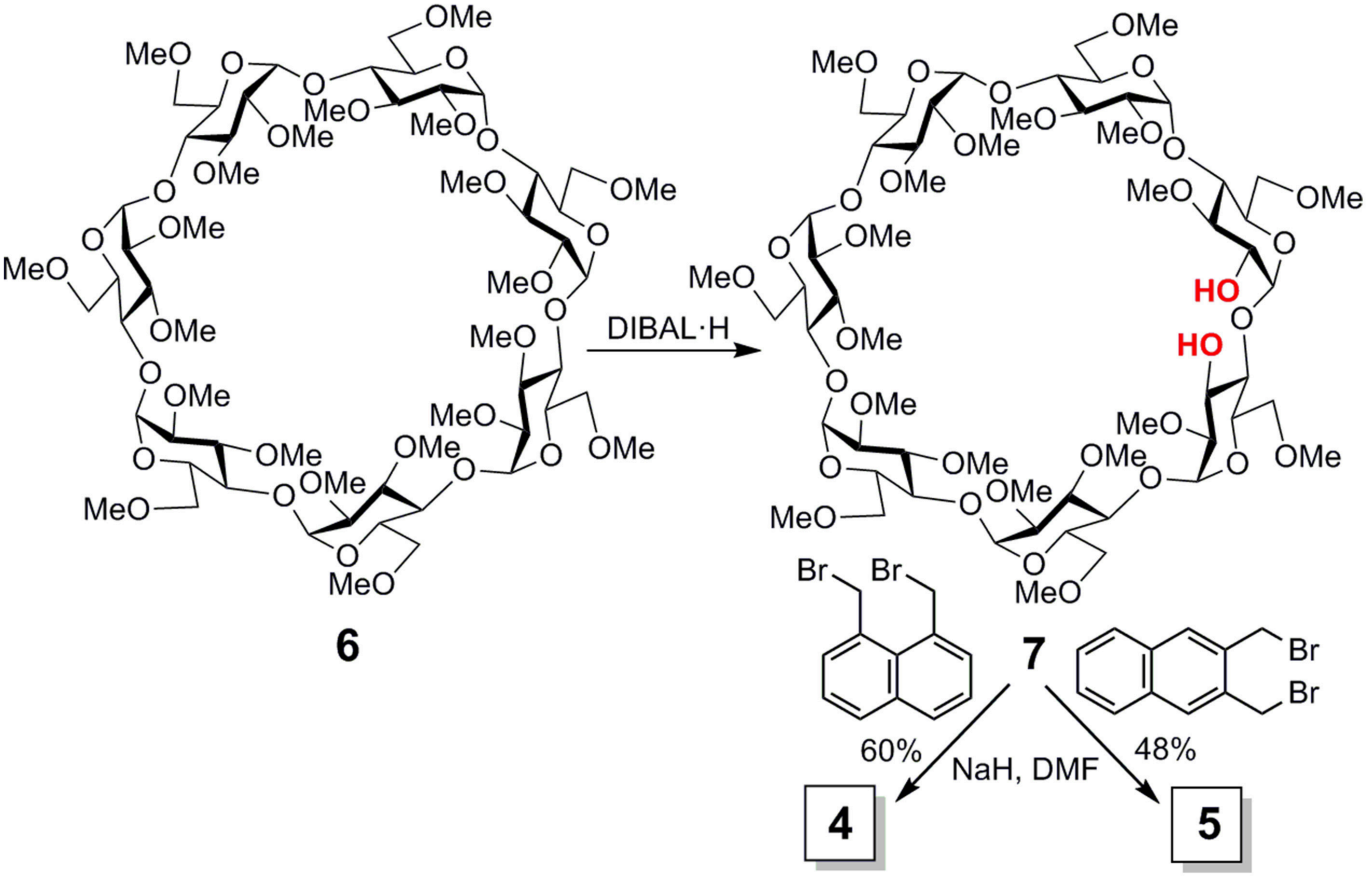
Synthesis of compounds **4** and **5**.

Reagents and solvents were purchased from commercial sources and used without further purification. ^1^H (^13^C) NMR spectra were recorded at 600, 500 and 400 (150.8, 125.7, and 100.6) MHz. 2D COSY, 1D and 2D TOCSY, NOESY, ROESY, HSQC, and HMBC experiments were used to assist assignments. Thin-layer chromatography (TLC) was carried out on aluminum sheets coated with Kieselgel 60 F245 (E. Merck), with visualization by UV light and by charring with 10% H_2_SO_4_. Flash column chromatography was carried out on silica gel (230–400 mesh). Elemental analyses were performed at the Institute for Chemical Research (Sevilla, Spain). The target cyclic ether conjugates **4** and **5** were readily obtained by alkylation of the diol precursor **7**, obtained from **6** by regioselective bis-demethylation with diisobutylaluminiun hydride (DIBAL·H) (Roizel et al., 2002; Xiao et al., 2010), with ether 1,8- or 2,3-bis(bromomethyl)naphthalene (Kemp et al., 1980) (Figure 3; see the Supplementary Data and Supplementary Figures S1–S16 for experimental details and full characterization data).

### Mass Spectrometry

The electrospray ionization-high resolution mass spectrometry (ESIHRMS) experiments were performed using a LTQ-Orbitrap XL ETD instrument from Thermo Scientific operated in positive ionization mode with a spray voltage at +3.6 kV. Samples were prepared at 20 μg·mL^−1^ in methanol/water (20:80 v/v). Once prepared, the fresh solutions were continuously infused at 3 μL·min^−1^ using a 250-μL syringe. The applied voltages were 45 and 120 V for the ion transfer capillary and the tube lens, respectively. The ion transfer capillary was held at 275°C. The resolution level was set to 30,000 (*m*/*z* = 400) for all studies, while the *m*/*z* range was set to *m*/*z* 200–2,000 in the profile mode and in the normal mass ranges. The spectra were analyzed using XCalibur 2.0.7 acquisition software (Thermo Scientific) without smoothing and background subtraction. The automatic gain control (AGC) allowed accumulation up to 2 × 10^5^ ions. The maximum injection time was set to 500 ms using 1 μ scan count.

### Fluorescence Spectroscopy

Steady-state fluorescence measurements were performed by using a high sensitivity spectrofluorimeter, the SLM 8100C Aminco, equipped with a cooled photomultiplier and a double (single) monochromator in the excitation (emission) path. Excitation and emission slit widths were selected at 8-nm for both channels. Polarizers were set at the magic angle conditions. The fluorescence decay measurements were achieved on a Time Correlated Single Photon Counting (TCSPC) FL900 Edinburgh Instruments Spectrometer. A NanoLed (Horiba), which emits short repetitive optical pulses at 296 nm, was used as a light source to excite the naphthyl groups. The system was equipped with two concave grating monochromators at both the excitation and emission paths and a red sensitive photomultiplier also immersed in a Peltier cooled housing. No polarizers were used. Data acquisition was carried out by using a multichannel time detector and a time window width of 200 ns with a total of 10,000 counts at the intensity maximum. The instrumental response function was regularly obtained by measuring the scattering of a Ludox solution. The cells housing temperature for both instruments were thermostatically controlled by using a Pt-100 probe with digital temperature processors. Right angle geometry and cylindrical quartz 2-mm inner path cells of 120 μL capacity were used for most of the experiments. Fluorescence intensities due to the inner effect were corrected (I_*corr*_) according to

(1) $${\text{I}}_{corr}={\text{I}}_{obs}\;10^{\left(\frac{{\text{A}}_{ex}+{\text{A}}_{em}}{2}\right)}$$

Where A_*ex*_ and A_*em*_ are the absorption at the wavelength of excitation and emission, respectively.

However, for some of the checking typical rectangular (square cross-section) quartz 1-cm path cells were used.

Decay intensity profiles were fitted to a sum of exponential decay functions as

(2) $$\text{I}(\text{t})\text{=}\sum_{\text{i=1}}^{\text{n}}{\text{A}}_{\text{i}}{\text{e}}^{-\text{t/}\tau_{\text{i}}}$$

by the iterative deconvolution method (O'Connor et al., 1979). The average lifetime of a multiple-exponential decay function was then defined as,

(3) $$\langle \tau \rangle =\frac{\sum_{\text{i=1}}^{\text{n}}A_{i}\tau_{i}^{2}}{\sum_{\text{i=1}}^{\text{n}}A_{i}\tau_{i}}$$

where A_i_ is the pre-exponential factor of the component with a lifetime τ_i_ of the multi-exponential function intensity decay.

The fractional contribution *f*_i_ of each decay time to the steady-state intensity, which represents the fraction of total fluorescence intensity *I* of the *i*-component at the wavelengths of observation, is given by

(4) $$f_{i}\;=\frac{{\text{A}}_{\text{i}}\tau_{i}}{\sum_{\text{i=1}}^{\text{n}}{\text{A}}_{\text{i}}\tau_{i}}=\frac{{\text{I}}_{\text{i}}}{\sum_{\text{i=1}}^{\text{n}}{\text{I}}_{\text{i}}}$$

and the intensity weighted average lifetime <τ> from a dilute solution of a pair of emitting species, 1 and 2, that do not interact during the excited state lifetime can be obtained as,

(5) $$\begin{array}{r} <\tau >={\text{f}}_{1}\tau_{1}+{\text{f}}_{2}\tau_{2} \end{array}$$

From the fluorescence depolarization measurements, the anisotropy *r* is typically defined as:

(6) $$r=({\text{I}}_{\text{VV}}-{\text{GI}}_{\text{VH}})/({\text{I}}_{\text{VV}}\text{+ }{\text{2GI}}_{\text{VH}})$$

where *I*_xy_ is the intensity of the emission that is measured when the excitation polarizer is in position x (V for vertical, H for horizontal), the emission polarizer is in position y, and the *G* factor (= *I*_HV_/*I*_HH_) corrects for any depolarization produced by the optical system (Lakowicz, 2008).

For a single isolated excited chromophore which is dynamically quenched by a quencher Q, the τ/τ_0_ ratio (with/without Q) is related with [Q] by the linear Stern-Volmer equation. For more complicated systems, the Stern-Volmer representations of <τ>/<τ_0_> are linear at the lowest [Q] region.

### Circular Dichroism

Circular dichroism spectra were obtained by using a JASCO-715 spectropolarimeter. Recorded spectra were the average of three scans taken at a speed of 50 nm min^−1^ with a time response of 0.125 s. The sensitivity and resolution were fixed at 20 mdeg and 0.5 nm, respectively. Measurements were performed in 1 cm or 0.1 cm path quartz cells at 25°C.

### Molecular Mechanics (MM) and Molecular Dynamics (MD) Simulations

All calculations were performed with Sybyl X2.0 *(SYBYL- X 2.0; Tripos Associates; ed. St. Louis, Missouri, USA, 2012*) and the Tripos force field (Clark et al., 1989). A relative permittivity ε = 3.5 (ε = 1) was used in the vacuum (in the presence of water). Charges for βCD derivatives were obtained by MOPAC (Frisch et al., 2004). The starting modified βCD derivatives were built with the macroring in the non-distorted form (ϕ = 0°, ψ = −3°, τ = 121.7° and side chain χ angles in the *trans* conformation) (Pozuelo et al., 1996) and the naphthyl substituent for **4** and **5** in the most probable conformations for the chain that links the naphthalene moiety to a glucopyranose unit of the βCD macroring. The selected conformations were those of the minima potential energies obtained by placement of the four torsional angles that describe the rotation around C(3)-O-CH_2_-C^ar^(1) and C(2)-O-CH_2_-C^ar^(8) ether bonds according to the procedures described elsewhere (González-Álvarez et al., 2011). Optimizations were carried out by the simplex algorithm, and the conjugate gradient was used as a termination method with gradients of 0.2 (0.5) kcal·mol^−1^Å^−1^ for the calculations carried out in vacuum (water) (Brunel et al., 1975; Press et al., 1988). Non-bonded cut-off distances were set at 12 Å. The Molecular Silverware algorithm (MS) and periodic boundary conditions (PBC) were used for system solvation when water was used as a solvent (Blanco, 1991).

The methods used for the conformational study of the monomeric **4** and **5** and for their dimer formation were similar to those described previously for **1** (González-Álvarez et al., 2008) and **2** or **3** (Neva et al., 2018). 2-ns MD simulations in the vacuum were performed at several temperatures ranging from 250 to 600 K on initial **4** and **5** conformations.

Emulation of dimerization processes were performed starting from the optimized conformations of **4** and **5**. A single head-to-head orientation (HH) for the CD-to-CD approaching, in agreement with experimental findings, was considered (see Supplementary Figure S30 for the coordinate systems describing dimerization). Critical analysis of the structures generated by scanning the θ [*O(4)-o-o'-O(4*′*)*] dihedral angle in the −180 to +180^o^ range (initially at 30° intervals and 10^o^ later) and the *y* coordinate (*oo*' distance) from 2 to 0.7 nm (0.05 nm intervals) in the presence of water (fixed the ε [*o-o'-O(4*′*)*] angle at 90^o^) followed by optimization (gradient 1.5 kcal·mol^−1^Å^−1^) provided the most favorable θ angle for approaching. Supplementary Figure S29 depicts an example for dimerization of **5**. Once θ was fixed, the dimerization was emulated by approaching along the *y* coordinate, in 0.05 nm steps, from 2 to 0.7 nm (Supplementary Figure S30). This was also done in the presence of water (MS and PBC). Every structure was optimized (1.5 kcal · mol^−1^Å^−1^) and saved for further analysis. Minima binding energy (MBE) structures for dimers were optimized once again (gradient 0.5 kcal · mol^−1^Å^−1^) and used as the starting conformations for the 1.0 ns MD simulations following the same strategy described earlier (González-Álvarez et al., 2008; Gonzalez-Álvarez et al., 2009b; González-Álvarez et al., 2011; Neva et al., 2018).

For computing complexation of **4-**dimer with sodium adamantane-1-carboxylate (AC), we started from the most stable HH MBE **4**-dimer structure. The anionic AC guest with the most favorable orientation (Supplementary Figure S41) was approached along the y coordinate from 2 to −2 nm (0.05 nm intervals) in the presence of water, in a way that is similar to the dimerization processes. Each structure generated and optimized in the presence of water was analyzed. In a similar manner, anionic AC complexation with **5** monomer (MBE structure) was computed.

### NMR Titration Experiments

Association constants (*K*_*a*_) for the inclusion complexes were determined in phosphate-buffered D_2_O (pD 7.4, 0.1 M) at 298 K by measuring the chemical shift variations in the ^1^H NMR spectra (500 MHz) of a solution of the CD derivatives **4** or **5** in the absence and in the presence of increasing amounts of AC. Typically, a stock solution of CD (ca. 0.5–1 mM) was prepared. A 500 μL aliquot of this solution was transferred to a NMR tube and the initial NMR spectrum was recorded. Then, a solution of AC (15–20 mM) was prepared in the stock CD solution (it is critically important to maintain a constant host concentration, pD and ionic strength throughout the titration experiment). Aliquots of the guest solution were sequentially added to the NMR tube and the corresponding spectrum was recorded after equilibration (Supplementary Figures S37, S38). Additions and measurements were continued until 90–100% complexation had been achieved. The chemical shifts of selected host resonances obtained at approximately ca. 15 different host-guest concentration ratios were plotted against the concentration of CD and used in an iterative least-squares fitting procedure using a 1:1 stoichiometry binding model (Bisson et al., 1998, 2000). Errors are estimated in ±15%.

## Results and Discussion

### Synthesis and Characterizations

Imposing distance restrictions in CDs through covalent bonds can lead to significant alterations in their primary structure, usually by provoking conformational changes in one or several of the monosaccharide building blocks, which may substantially perturb the inclusion capabilities (Fujita et al., 1988; Fukudome et al., 2007b; Álvarez-Dorta et al., 2015, 2016; Immel et al., 2001; Fukudome et al., 2007; León et al., 2018). Compounds **4** and **5** were purposely conceived to avoid this and keep the toroidal βCD cavity characteristic of βCD undistorted, so that any effect in their supramolecular properties can be ascribed to the presence of the naphthalene component, devoid from additional effects on the βCD macroring topology. According to the available X-ray data for permethylated βCD **6** (Caira et al., 2004), the through-space distance between the O-2^I^ and O-3^II^ positions in the cyclooligosaccharide backbone is 3.4 Å, which can be accommodated by benzylic positions in either 1,8- (for **4**) or 2,3-disubstituted (for **5**) naphthylene clips (separation distance <4.1 Å) without provoking any significant steric strain. Indeed, alkylation of the βCD diol precursor **6** with 1,8- or 2,3-bis(bromomethyl)naphthalene, conducted in dry N,N-dimethylformamide (DMF) in the presence of sodium hydride (NaH), afforded the target cyclic naphthylene ethers **4** and **5**, respectively, as single products, as seen from thin layer chromatography (TLC) and mass spectrometry (MS) monitoring of the crude reaction mixtures (Figure 3). No side-products resulting from cross-linking or single-point etherification reactions were detected, pointing to a fast ring-closure once the bis(bromomethyl)naphthalene tether first reacts at either O-2^I^ or O-3^II^. Analytically pure samples of **4** and **5**, were isolated in 56 and 48%, respectively, after column chromatography on silica gel using 100:1 → 20:1 EtOAc/EtOH as eluent.

The ^1^H and ^13^C NMR data for **4** and **5** confirmed the presence of the dual-bridged joint involving consecutive glucopyranosyl subunits, with all the pyranoid rings in the ground state ^4^*C*_1_ chair conformation, in agreement with full preservation of the basket-shaped architecture. Selective irradiation experiments (1D TOCSY, Supplementary Figures S7, S15 for **4** and **5** in the Supplementary Materials, respectively) featured ^3^*J*_H, H_ vicinal coupling constants in the 9–10 Hz range for the non-anomeric sugar protons (4.1–3.1 ppm), indicative of all-axial arrangements for the H-2, H-3, H-4, and H-5 protons about the six-membered pyranoid rings. The anomeric H-1 protons (5.32–5.14 ppm) exhibited coupling constant values in the 3.8–4.1 Hz, in agreement with the α-configuration. ROESY spectra further evidenced the expected intra-monosaccharide H-3—H-5 and inter-monosaccharide H-1—H-4 spatial correlations characteristic of undistorted cyclodextrin derivatives (Supplementary Figures S3, S10 for **4** and **5**, respectively, in the Supplementary Materials). In the case of **4**, the 1,8-dimethylnaphthylene clip (^1^H NMR resonances in the 8.0–7.5 ppm region) draws a 12-membered macrocycle with a five-carbon segment flattened region that imposes considerable rigidity, which is reflected in a broad dispersion of the benzylic proton resonances. The 2,3-dimethylnaphthylene clip in **5** (^1^H NMR resonances in the 7.9–7.5 ppm region), in which the flattened segment in the bridging area is reduced to four-carbon centers, is expected to keep substantially higher flexibility. Indeed, the corresponding proton NMR spectrum closely resembled the spectrum of the *o*-xylylenated βCD derivative **2** (Supplementary Figure S16).

### Self-Assembling

The 2,3-naphthylene-equipped βCD derivative **5**, but not the 1,8-naphthylene positional isomer **4**, exhibited a very apparent negative solubility coefficient in water: when heated, clear (25°C) solutions became immediately turbid, returning to the limpid original state upon cooling. A negative water solubility coefficient is a characteristic feature of methylated cyclodextrin derivatives bearing aromatic appendages and can be ascribed to reversible dimerization processes (Filippone et al., 2002): in the dimer state, the hydrophobic aromatic moiety is shielded from the bulk, facilitating solvation and preventing unspecific clusterization at low temperatures, whereas heating results in dimer disruption, uncovering of the aromatic ring and precipitation. The dissimilar behavior of **4** and **5** was, however, intriguing. In principle, it could be interpreted in terms of differences in the propensity of **4** and **5** to dimerize. Notwithstanding, the high resolution electrospray ionization (HRESI)-mass spectra of **4** and **5**, recorded from aqueous solutions, both exhibited prominent pseudomolecular peaks for the dimer species (Supplementary Figure S17). The data can be concealed assuming that the aromatic surface in the more rigid derivative **4** is similarly exposed to the solvent in the monomer and dimer entities, possibly acting as cavity extension module, whereas in **5**-dimer the naphthalene walls are probably sandwiched between the two βCD constituents, in agreement with our starting hypothesis.

**Figure 4:**
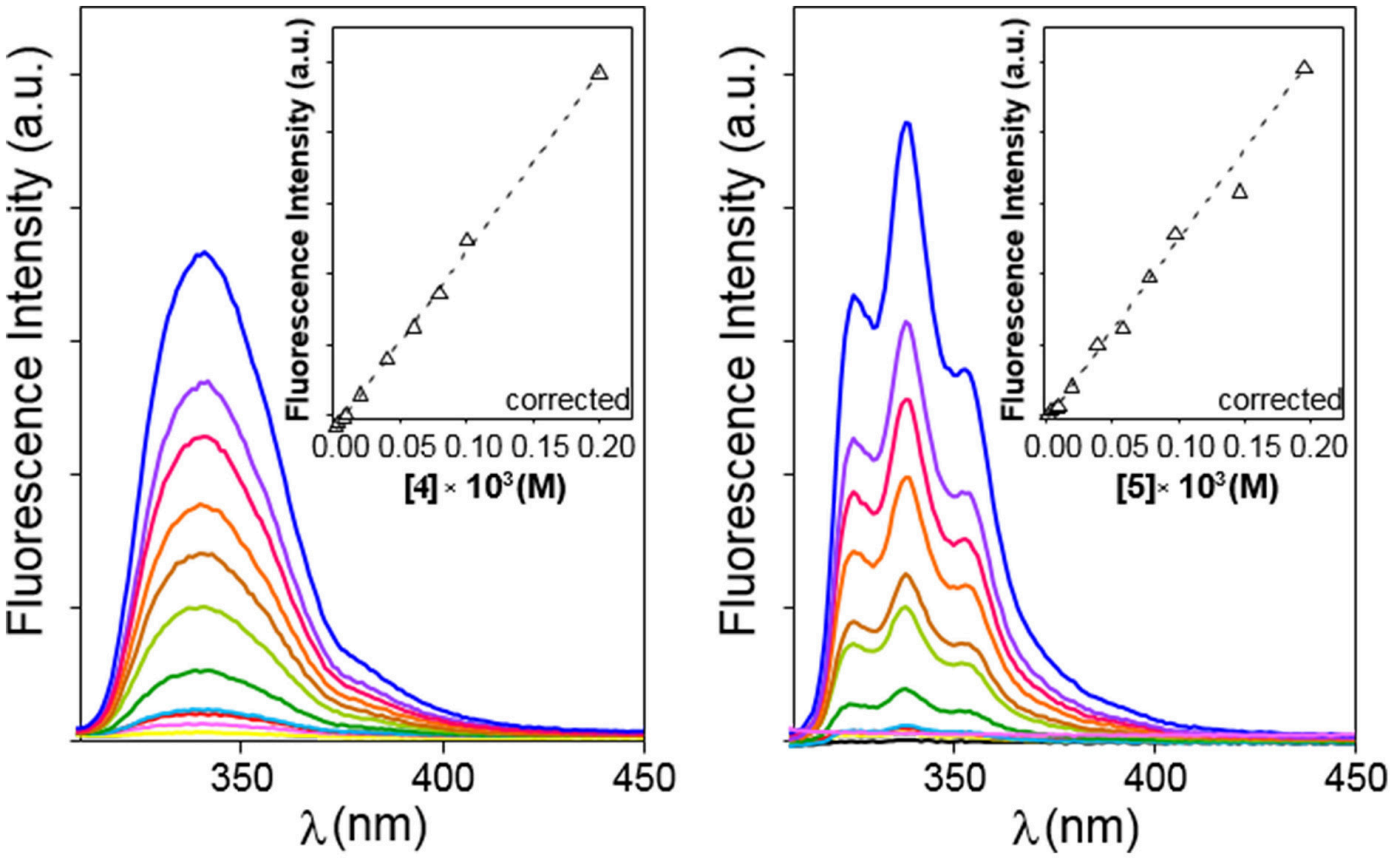
Emission spectra for **4 (Left)** and **5 (Right)** in water in the 0 to ~0.2 mM range of concentrations (yellow to blue solid lines) at 25°C. Variation of the corrected fluorescence intensity (Equation S1) with compound concentration measured at the intensity maxima are superimposed (dashed lines).

**Figure 5:**
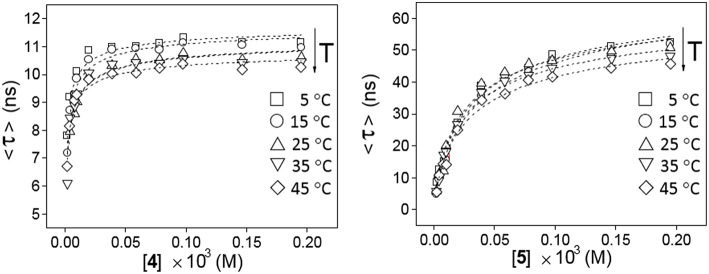
Variation of the weighted average lifetime <τ> with the concentration of **4 (Left)** or **5 (Right)** in water in the 5–45°C temperature range.

The possibility to drive the architecture of the CD dimer species by changing the type of joint to the naphthylene clip was further substantiated by fluorescence spectroscopy. Figure 4 shows the emission spectra for water solutions of **4** and **5** at increasing concentrations (from 0 to ~0.2 mM) at 25°C upon excitation of the naphthalene chromophore (295 nm). The spectrum for **4** exhibits a single band located at ~340 nm and a shoulder at ~380 nm, whereas the spectrum for **5** shows a double band with two maxima placed at ~330 and ~340 nm and a shoulder at ~350 nm. The low intensity shoulders located at longer wavelengths suggest the presence of intermolecular aromatic excimers, that is, the formation of short-lived dimeric complexes in an electronically excited state (Saigusa and Lim, 1996; Shirai et al., 2016). Conclusive confirmation of dimer formation was obtained from the analysis of the corresponding fluorescence decay profiles obtained from time-resolved fluorescence measurements, selecting the maximum of emission (340 nm in both βCD derivatives **4** and **5**). The data were fitted to three-exponential decay functions at any concentration and temperature: a short-lived component (<0.5 ns) ascribed to the inherent scattering of the cylindrical cuvettes (not taken into account in the analysis), a second intermediate component in the 2–9 ns (for **4**) or 3–12 ns (for **5**) range that was attributed to the free dissociated monomer and a third slowest component in the 10–15 ns (for **4**) or 20–60 ns (for **5**), whose contribution increased with concentration and decreased with temperature, which was attributed to the dimeric entity (**4**-dimer or **5**-dimer). In full agreement, the lifetime averages (<τ>) of the excited electronic states (obtained from Equation S3) increased with the concentration of **4** or **5** (Figure 5), a consequence of the increase of the fraction of the dimer species in the corresponding monomer/dimer equilibria.

Plots of the corrected fluorescence intensity (I_*corr*_, Equation 1 in the Experimental section), measured as the area under the emission spectra, against the concentration of **4** and **5** in water at 25°C (Figure 4 inserts) were linear in the whole range of concentrations used, meaning that the fluorescence quantum yield ϕ, i.e., the ratio of the number of photons emitted to the number of photons absorbed, does not change upon dimerization (ϕ_dimer_ ≅ ϕ_monomer;_ see Equation 8 in the Experimental section). In other words, the fluorescence intensity is not sensitive to the association process of **4** or **5**. Under this assumption, the dimerization isotherms fit the experimental data to Equation (9) (Experimental section). From them, the respective dimerization constants (*K*_D_), as well as the lifetimes for the monomeric (τ_0_ = τ_monomer_) and dimeric (τ_∞_ = τ_dimer_) forms of both dimethylnaphthylene-clipped βCD derivatives **4** and **5**, were derived. Table 1 collects the ensemble of data for the whole range of temperatures. Remarkably, the propensity to form dimers is about 2-to-3 orders of magnitude higher as compared with the previously reported *o*-xylylene clipped βCD derivative **2** (Neva et al., 2018). As expected from the shape of the isotherm plots in Figure 5, where a plateau is reached at much lower concentrations for **4** than for **5**, the *K*_D_ values for **4**-dimer formation (4.1–8.0 · 10^5^ M^−1^) were found to be above one order of magnitude higher as compared with the corresponding values for **5**-dimer formation (1.4–2.2 · 10^4^ M^−1^). On the other hand, the significantly larger average lifetimes at infinite concentration (ascribed to the dimer form) for **5** (67.5–79.8 ns) than for **4** (11.0–11.9 ns) point to considerable differences in the structure of the corresponding dimers, reinforcing the notion that the naphthylene moiety is probably better shielded from the solvent in **5**-dimer than in **4**-dimer.

**Table 1:** Dimerization constant (*K*_D_) values and lifetime of the electronic excited state for the monomer (τ_monomer_) and dimer (τ_dimer_) species obtained from analysis of decay profiles at different concentrations and temperatures for CDs **4** an **5**.

**Compound**	**Temperature (^**°**^C)**	**10^**−5**^*K*_**D**_ (M^**−1**^)**	**τ_**monomer**_ (ns)**	**τ_**dimer**_ (ns)**
4	5	8.0 ± 1.5	2.7 ± 0.4	11.9 ± 0.2
4	15	5.6 ± 0.9	2.8 ± 0.3	11.9 ± 0.2
4	25	4.1 ± 0.6	2.3 ± 0.3	11.6 ± 0.2
4	35	5.6 ± 1.3	2.0 ± 0.4	11.4 ± 0.2
4	45	6.8 ± 1.2	2.4 ± 0.3	11.0 ± 0.2
5	5	0.18 ± 0.02	3.2 ± 0.8	77.9 ± 3.1
5	15	0.14 ± 0.02	2.6 ± 0.8	79.8 ± 3.8
5	25	0.21 ± 0.05	2.4 ± 1.3	74.4 ± 4.6
5	35	0.22 ± 0.03	2.3 ± 0.8	69.7 ± 2.6
5	45	0.19 ± 0.03	2.6 ± 0.8	67.5 ± 3.0

**Figure 6:**
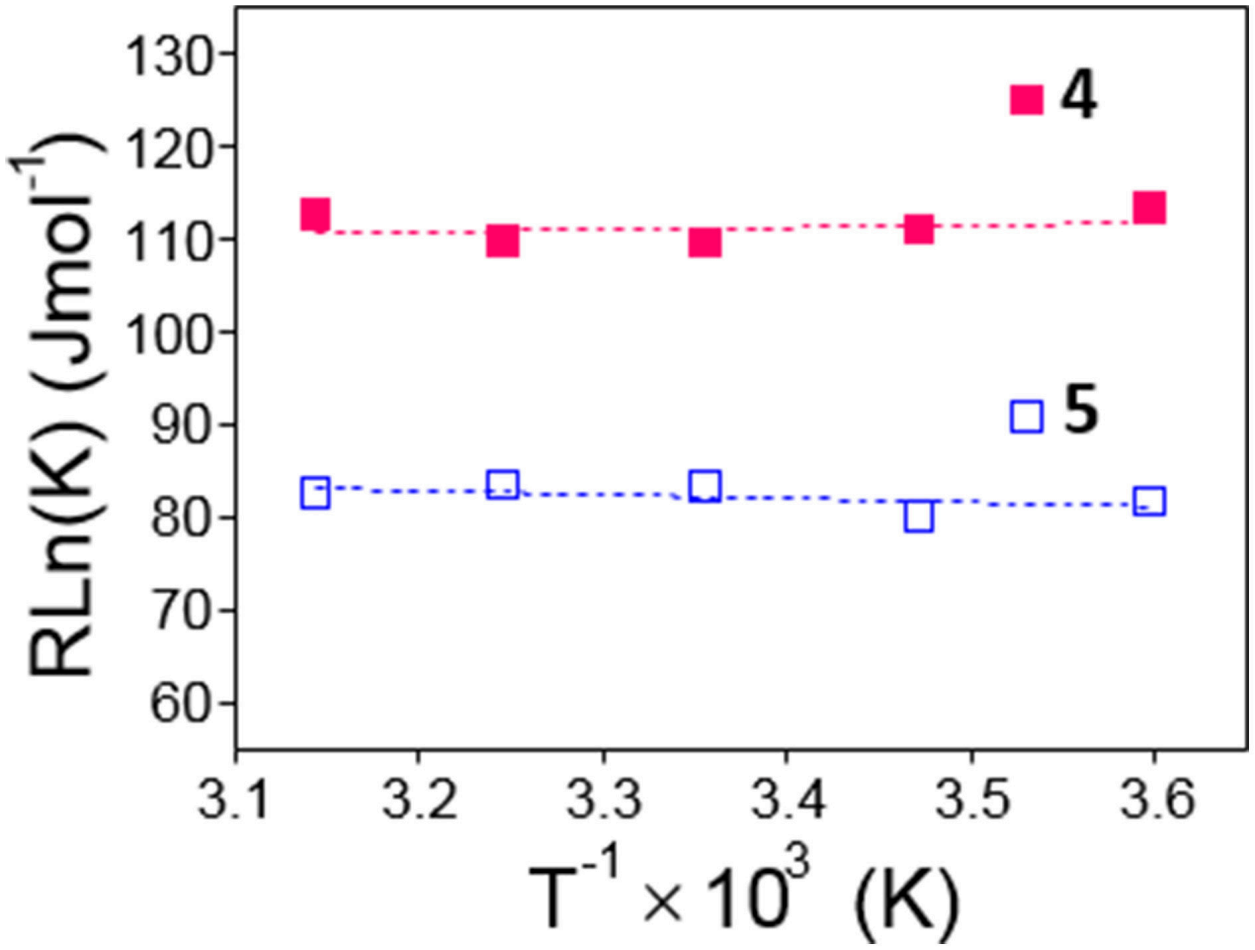
Van't Hoff linear representations for the dimerization processes of **4** and **5** obtained from the association constants collected in Table 1.

From the van't Hoff linear representations shown in Figure 6, the Δ*H* and Δ*S* thermodynamic parameters associated to the dimerization process were found to be Δ*H* = −2.5 ± 5.1 kJ·mol^−1^, Δ*S* = +102.7 ± 17.3 J·K^−1^mol^−1^ for **4** and Δ*H* = +4.5 ± 3.6 kJ·mol^−1^, Δ*S* = +97.3 ± 12.2 J·K^−1^mol^−1^ for **5**. Van der Waals or electrostatics favorable intermolecular interactions are usually characterized by relatively large Δ*H* < 0 values, whereas hydrophobic forces, which tend to reduce the hydrophobic surface of non-polar moieties, are instead accompanied by near zero or slightly positive Δ*H* values (Southall et al., 2002; Meyer et al., 2006; Kronberg, 2016). The experimental Δ*H* data are therefore consistent with a major role of the dimethylnaphthylene appended group in the dimerization process. The rather large and favorable Δ*S* values accompanying monomer-to-dimer transformation for **4** or **5** are also consistent with hydrophobic interactions as the driving force. They can be ascribed to a substantial loss of solvating shells during dimerization due to the large size of the naphthylene moiety, which overcomes the intrinsic entropy decrease due to the loss of degrees of freedom accompanying the association of monomeric entities. The preponderant role of hydrophobic forces was further confirmed by conducting fluorescent decay measurements for solutions of **4** and **5** in solvents of decreasing dielectric constant (water, water-methanol mixtures, methanol and ethanol; Supplementary Figures S18, S19): a steep decrease in <τ> values was observed, which correlates with an increase in the proportion of the shorter lifetime monomer component at the expenses of the dimer. For instance, whereas a 1.5 · 10^−4^ M solution of **4** in water at 25°C contains 90% of **4**-dimer, in ethanol the dimer proportion drops to 40%.

### Structure and Conformation

In order to obtain information on the topology of the 1,8- and 2,3-dimethylnaphthylene βCD conjugates **4** and **5** and on the changes that may occur in the location of the naphthyl chromophore upon dimer formation, fluorescence quenching experiments using diacetyl as the quencher (q) were next conducted. Measurements were carried out at 25°C in water for two different concentrations of **4** (0.02 and 0.2 mM, meaning **4**-dimer molar fractions of 0.69 and 0.88, respectively, for *K*_D_ = 4.1·10^5^ M^−1^; Table 1) or **5** (0.03 and 0.2 mM, meaning **5**-dimer molar fractions of 0.25 and 0.53, respectively, for *K*_D_ = 0.21·10^5^ M^−1^; Table 1). From the Stern–Volmer (<τ>_q=0_/< τ > vs. [q]) plots (Lakowicz, 2008) (Supplementary Figure S24), which were linear in the range of quencher concentrations used (0 to 8 mM), the Stern-Volmer constants (*K*_sv_) and bimolecular quenching constants (*k*_q_) were obtained (Table 2). For species bearing the same chromophore unit, *k*_q_ values provides a qualitative assessment of the differences in the accessibility to the quencher: the lower the *k*_q_ value, the less accessible is the chromophore. Changes in the concentration of **4** did not affect *k*_q_, suggesting that the 1,8-naphthylene group was similarly exposed in the monomer and in the dimer (*k*_q_ = 1.4 ·10^9^ M^−1^s^−1^). Differently, the *k*_q_ value for water solutions of **5** decrease by about 15% on going from the lower (*k*_q_ = 0.6 · 10^9^ M^−1^s^−1^) to the higher concentration (*k*_q_ = 0.5 · 10^9^ M^−1^s^−1^), indicating that the 2,3-naphthylene moiety is less accessible to the quencher in **5**-dimer as compared to the monomer form. The data could be interpreted as the shielding of the aromatic clip between the macrorings of two βCD units upon dimerization in the case of **5** but not in the case of **4**, which points out to substantial differences in the architecture of **4**-dimer and **5**-dimer. Additionally, the lower *k*_q_ values for **5** than for **4** whatever the concentration indicates that the 2,3-naphthylene moiety in **5** is less exposed to the quencher than the 1,8-naphthylene module in **4**, both in monomer and dimer form, which further advocates for strong differences in the orientation of the aromatic appendage relative to the cyclooligosaccharide scaffold.

**Table 2:** Stern-Volmer plot parameters for the quenching **4** and **5** by diacetyl of at different concentrations at 25°C.

**Compound**	**Concentration (mM)**	***X*_**dimer**_**	**<τ>_q = 0_ (ns)**	***K*_****SV****_ (M^**−1**^)**	***k*_****q****_ × 10^**−9**^ (M^**−1**^s^**−1**^)**
4	0.02	0.69	10.5 ± 0.1	15.0 ± 0.2	1.4 ± 0.1
4	0.2	0.88	10.3 ± 0.1	14.3 ± 0.6	1.4 ± 0.1
5	0.03	0.25	45.6 ± 0.1	29.7 ± 0.7	0.6 ± 0.3
5	0.2	0.53	49.5 ± 0.1	24.7 ± 0.5	0.5 ± 0.2

The presence of the naphthylene chromophore in **4** and **5** enables circular dichroism as a useful technique for conformational analyses: βCD is a chiral molecule; although it does not absorb above 200 nm, a chromophore appended to βCD (or included in the cavity) exhibits induced circular dichroism (ICD) (Superchio et al., 2004; Berova et al., 2007). Most interestingly, circular dichroism also provides precious information on intermolecular interactions and the structure of supramolecular assemblies: when two (or more) chromophores are positioned close in space and adopt a proper (chiral) mutual orientation, the interactions between their electronic transition dipoles result in large rotational strengths, often surpassing those connected with the perturbations on each chromophore exerted by the chiral non-chromophoric frame. The most significant case arises when chromophores with strong electric-dipole allowed transitions couple to each other (exciton coupling; EC) (Pescitelli et al., 2011).

**Figure 7:**
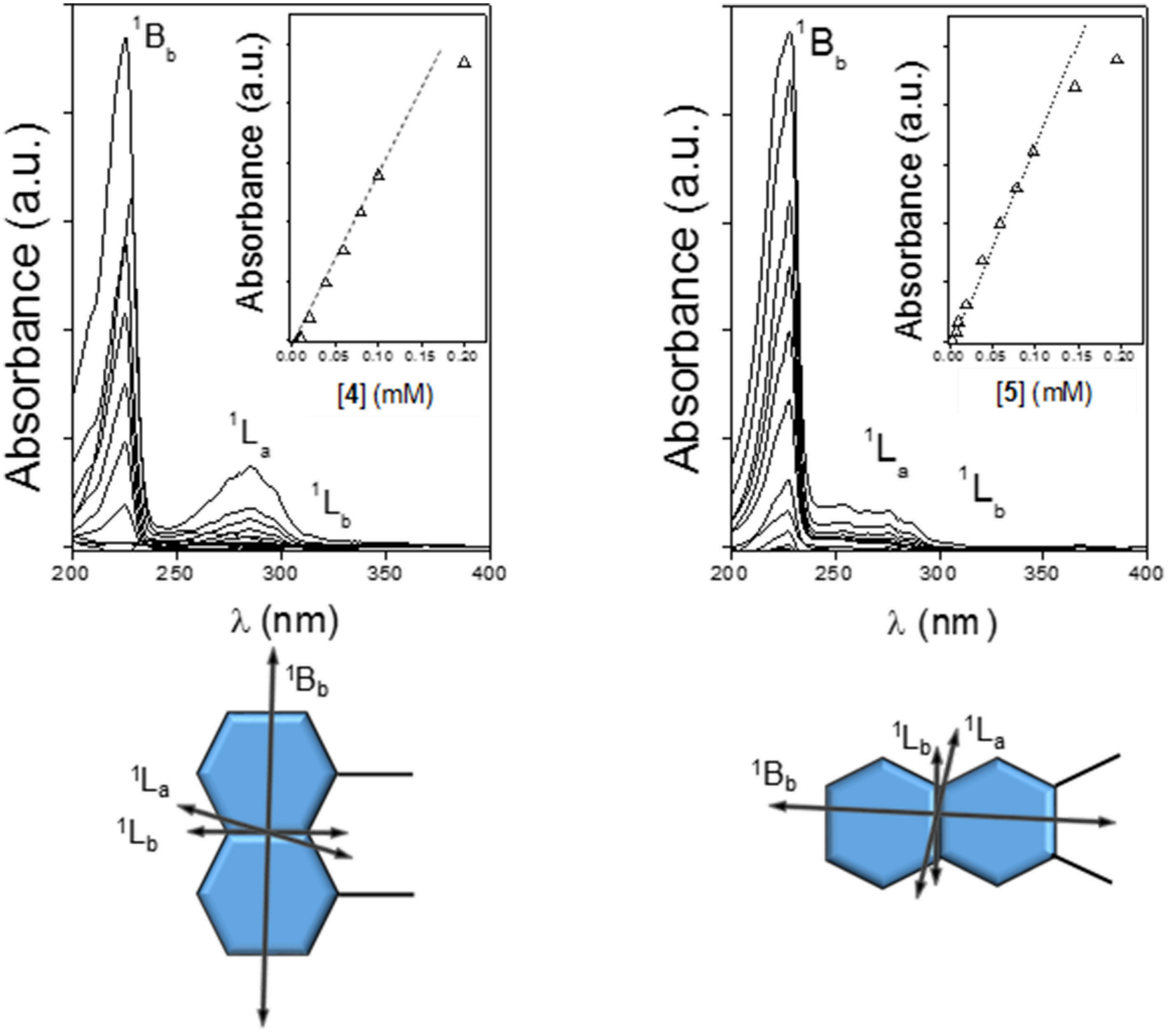
Absorption spectra for **4 (Top Left)** and **5 (Top Right)** in water at 25°C, with the corresponding absorbance vs. concentration plots (inserts) and directions of the electronic transition moments for the 1,8- **(Bottom Left)** and 2,3-naphthylene **(Bottom Right)** appended groups.

It has been established that the sign of ICD for a chromophore inside the βCD cavity is positive when the electron transition dipole moment is parallel to the axis of CD while the one perpendicular to the axis is negative (Harata and Hisashi, 1975; Shimizu et al., 1979, 1981, 1982). This rule has been extended to derive a general law that the sign of ICD is reversed when the guest moves from inside of the cavity to outside and the direction of the transition is fixed, and it changes at the angle between the transition moment and the CD axis 54.7° (Kodaka and Fukaya, 1989; Kodaka, 1991, 1993). The major UV transitions of the naphthyl group are ^1^B_b_ (~230 nm), ^1^L_a_ (~275 nm), and ^1^L_b_ (~320 nm), and they do not change substantially with substitution (Kodaka, 1998). In full agreement, the ordinary absorption spectra for **4** or **5** in water exhibit two intense main bands whose maxima are placed at ~220 or ~227 nm and ~285 or ~287 nm and a rather weak shoulder located at ~315 or ~339 nm, which were ascribed to the ^1^B_b_, ^1^L_a_, and ^1^L_b_ electronic transitions, respectively (Figure 7, top panels). Here we follow the broadly accepted Platt's notation, where the electronic state is designated by A, B, C, …or K, L, M, … in accordance with its total momentum number *Q* (or total ring quantum number) and the superscript “1” indicates a singlet state, whereas the subscripts “a” and “b” denote the location of the nodes of the wave function: with a and b, the nodes are indicated to be present on C–C bonds and on carbon atoms, respectively (Platt, 1949; Michl, 1978). The electron transition dipole moment of the short- and the high-wavelengths ^1^B_b_ and ^1^L_b_ transitions are nearly parallel and perpendicular, respectively, to the long axis of naphthalene, while that of the ^1^L_a_ transition is tilted about 10–40° from the short axis (Kodaka, 1998; González-Álvarez et al., 2009a) (Figure 7, bottom panels). In the next paragraphs, the assignment of the ICD bands for compounds **4** and **5** conforms to these literature reports.

**Figure 8:**
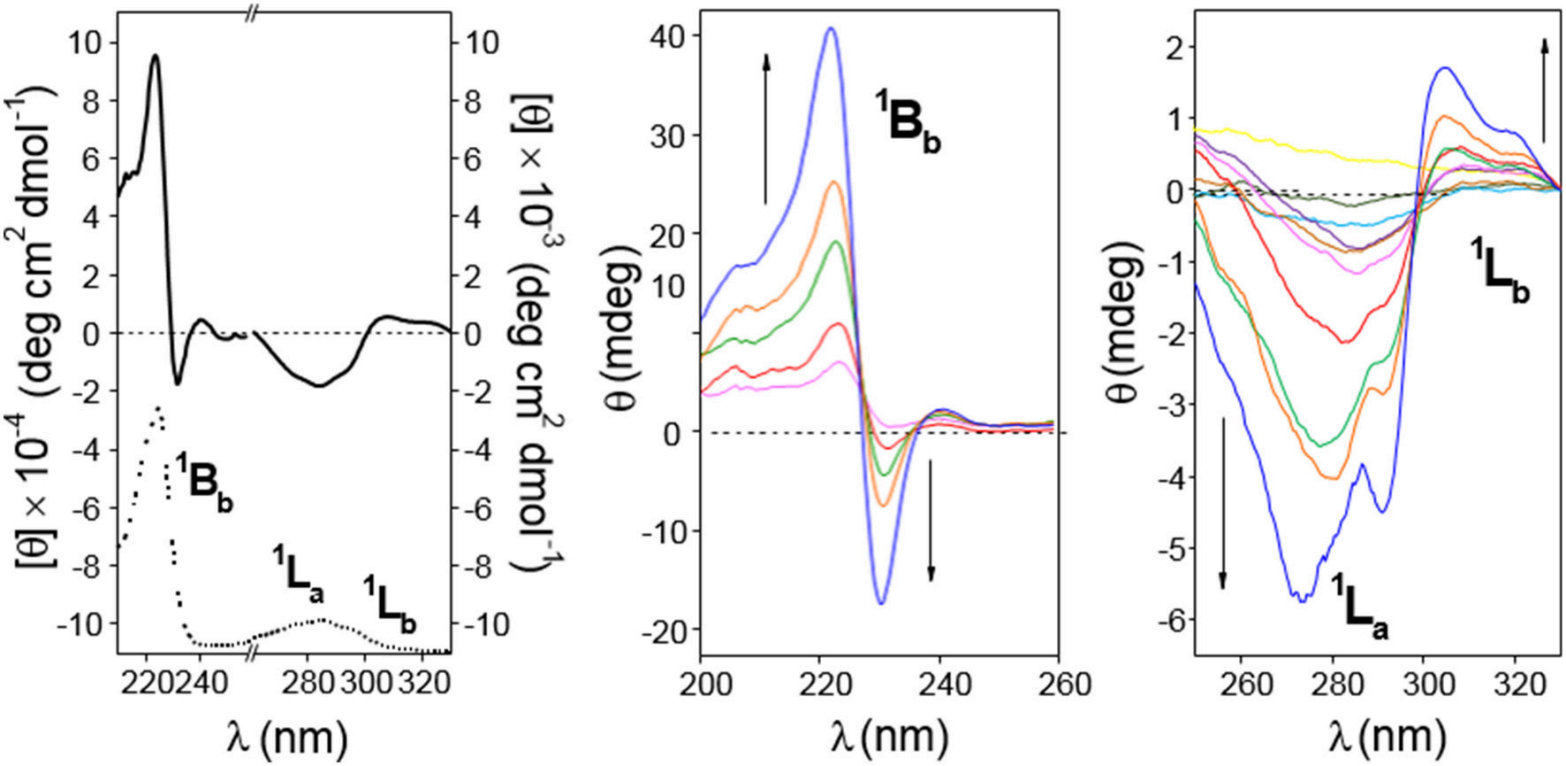
**(Left)** 210–330 nm range circular dichroism spectrum (solid line; as molar ellipticity [θ]), and absorption spectra (dotted line; arbitrary units) for **4** (0.11 mM in water). **(Middle** and **Right)** Circular dichroism spectra (as ellipticity; θ) for **4** in the 200–260 nm (from magenta to blue lines: 0.67, 1.12, 1.68 2.24, and 3.36 · 10^−4^ M in water; cell path 0.1 cm) and in the 260–330 nm range (from yellow to blue lines: 0.022 · 10^−4^ to 3.4 · 10^−4^ M in water; cell path 1 cm). All spectra were recorded at 25°C.

The circular dichroism spectrum (expressed as molar ellipticity; [θ]) of a 0.11 mM solution of **4** in water (**4**-dimer molar fraction 0.84; Figure 8, left panel) exhibits EC in the electronic ^1^B_b_ transition band, which represents a strong evidence for the presence of aggregates facing the naphthylene-bearing βCD secondary rims (head-to-head; HH dimers), likely stabilized through the mutual interaction between the aromatic groups. In addition, the ^1^L_a_ and ^1^L_b_ transitions provide negative and slightly positive bands, respectively. The intensities of all the three bands (expressed as ellipticity; θ, mdeg) in the ICD spectrum increased with the concentration of **4** (Figure 8, middle and right panels). At the lower concentrations used (0.67 · 10^−4^ and 0.022 · 10^−4^ M for the 0.1- and 1 cm path cell, respectively) the **4**-dimer molar fractions were ~0.81 and 0.37, respectively, which means that the ICD signals at the highest concentration were mostly due to the dimer species. Negative and slightly positive ^1^L_a_ and ^1^L_b_ bands then agree with the respective transition moments adopting a nearly parallel (for ^1^L_a_) and forming an angle slightly larger than 54.7° (for ^1^L_b_) with respect to the 7-fold macroring axis. These geometrical requirements could be fulfilled for a fully open conformation of the 1,8-dimethylnaphthylene-βCD conjugate where the naphthylene group, slightly rotated about his short axis, essentially prolongs the βCD walls.

**Figure 9:**
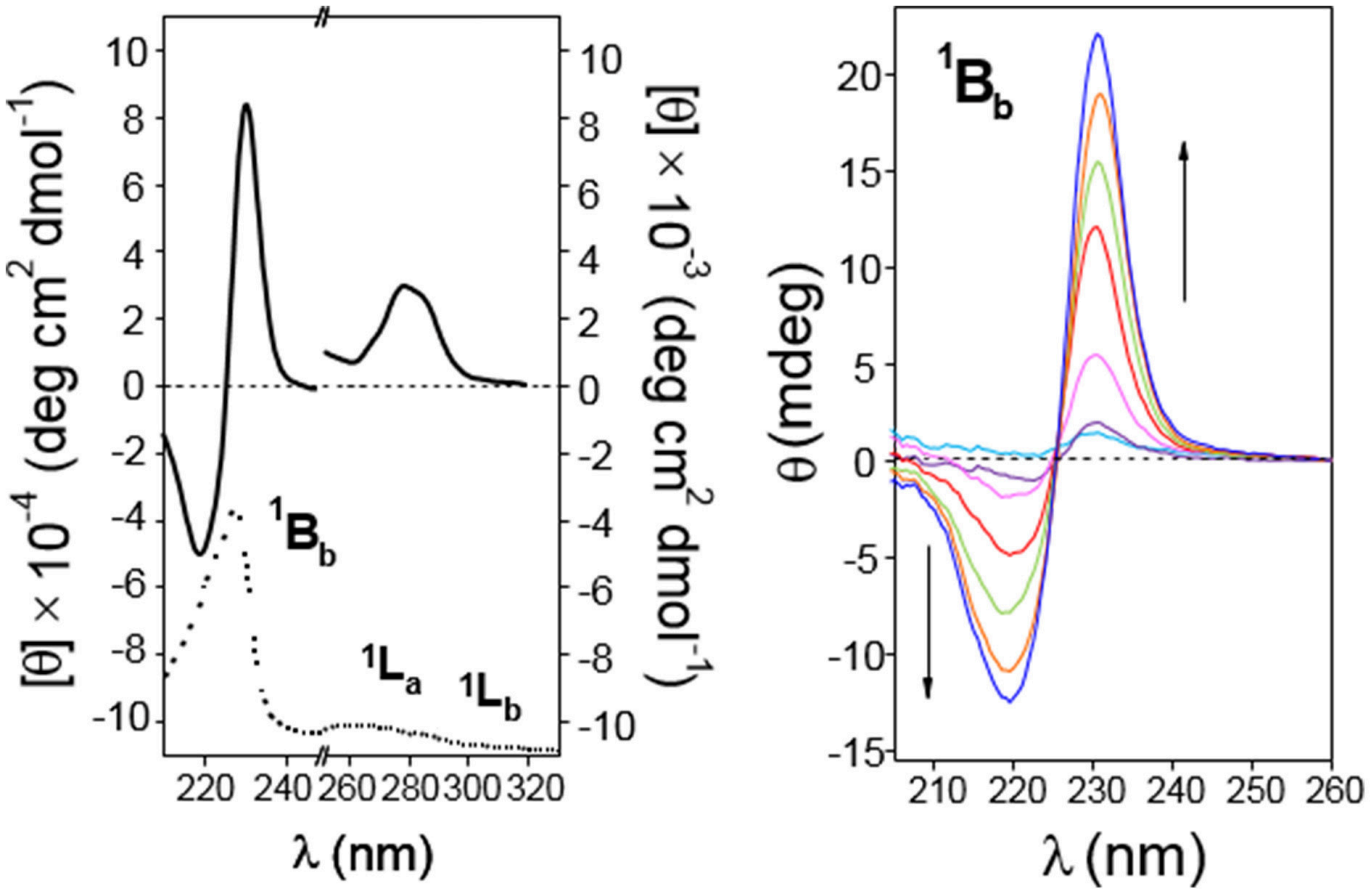
Circular dichroism spectra of aqueous solutions of CD **5**. **(Left)** Represents molar ellipticity ([θ], solid lines) and pure absorption (dotted lines) of a 0.30 mM solution in the 210–330 nm range. **(Right)** Represents the ellipticity (θ) at variable concentration (from light blue to blue lines: 0.17, 0.26, 0.54, 1.09, 1.63, and 2.73 · 10^−4^ M) in the 200–260 nm (path 0.1 cm). All spectra recorded at 25°C.

**Figure 10:**
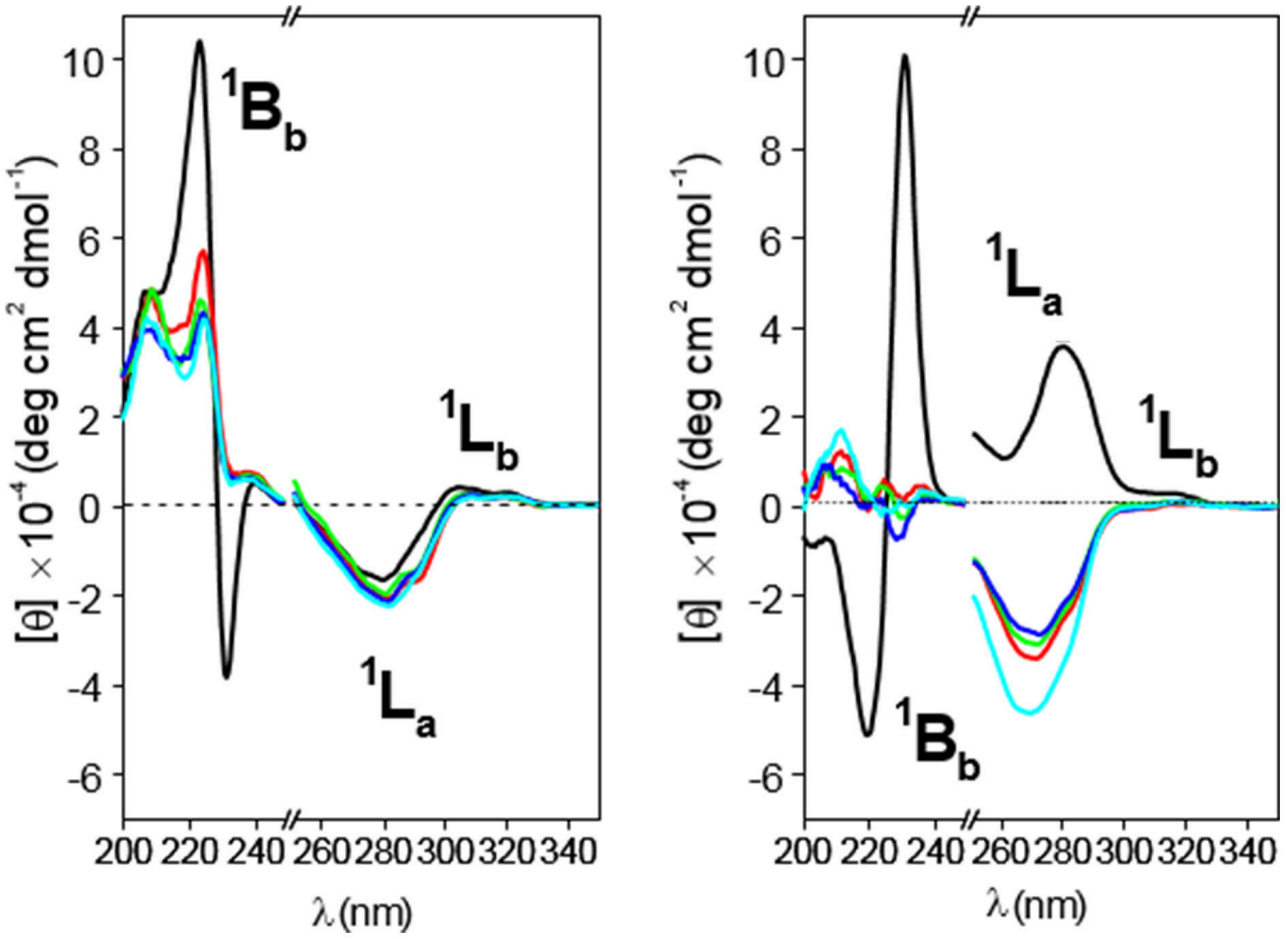
Circular dichroism spectra for **4 (Left)** and **5 (Right)** solutions in water (black line), 1:1 (v/v) water/methanol (red line), methanol (green line), ethanol (deep blue line), and propanol (light blue line) at 25°C. Concentrations were fixed at 0.19 and 0.20 mM for **4** and **5**, respectively.

The ICD spectra of a 0.3 mM solution of **5** in water (**5**-dimer molar fraction ~0.59; Figure 9, left panel) likewise exhibited EC in the ^1^B_b_ band, with peaks of similar intensity but opposite sign as compared with **4**, consistent with an HH dimer. This EC was still visible at lower concentrations down to 0.17 mM (**5**-dimer molar fraction ~0.51; Figure 10, right panel). The ^1^L_a_ and ^1^L_b_ transition bands were both positive and rather weak, especially the last one that was hardly visible. The ^1^L_a_ sign agrees with a naphthyl moiety placed outside the βCD cavity in a near perpendicular orientation relative to the macroring 7-fold axis, whereas the almost zero intensity of the ^1^L_b_ band is indicative of an angle of around 54.7° between the associated transition moment and the βCD main axis. This scenario would agree with a half-open conformation with a slightly long-axis rotated naphthylene group to provide the appropriate angles.

The occurrence of EC in both **4**-dimer and **5**-dimer, ascribable to HH species, had previously been detected in CD derivatives having a 1,8-naphthylene substituent attached at O-2^I^/O-3^I^ in the same glucopyranosyl residue (as the xylylene moiety in **1**, Figure 1). Only in the case of the γCD representative head-to-tail (HT) arrangements were found to predominate (González-Álvarez et al., 2013); for the αCD and βCD representatives the HH-dimer was the structure that better fitted most of the experimental and computational findings, as in the present work (González-Álvarez et al., 2011, 2013).

Circular dichroism spectra of **4** and **5** in solvents with decreasing polarity (water, 50:50 methanol/water, ethanol, propanol; Figure 10) led to the disappearance of the dual EC signal, which becomes a positive ^1^B_b_ band, in agreement with HH-dimer dissociation. This is an expected consequence of the weakening of hydrophobic attractive interactions and represents an additional evidence of the hydrophobic effect-driven nature of the dimerization process. Decreasing the medium polarity also makes the ^1^L_a_ band for **4** slightly more negative, whereas the ^1^L_b_ band still remains weakly positive (Figure 10, left panel). Taken together, positive ^1^B_b_ and negative ^1^L_a_ bands agree with a conformation for the monomer form of **4** where the naphthyl group is located outside the cavity with the ^1^B_b_ and ^1^L_a_ transitions moments nearly perpendicular and parallel, respectively, with respect to the βCD main axis, which essentially matches the situation encountered in **4**-dimer. It can be concluded that self-assembly of **4** into dimers proceeds without any significant conformational change in the constitutive monomers. Sharply differently, dimer dissociation of compound **5** upon decreasing solvent polarity entails sign inversion of the ^1^L_a_ band from positive to negative, the ^1^L_b_ band remaining almost negligible (Figure 10, right panel). This trend points to a conformational change involving a twist of the 2,3-dimethylnaphthylene clip when going from the dimer to the monomer state and vice versa: in the monomer form the aromatic clip portion orients the ^1^L_a_ transition moment nearly parallel to the macroring axis while keeping a half-open disposition with respect to the βCD scaffold. Dimerization of **5** in water implies therefore a fitting process characterized by rotations about the benzyl and ether-type bonds to optimally dispose the naphthalene surfaces. Variable temperature 1H NMR spectra of 4 and 5 (MeOD) as well as 1H NMR spectra recorded in solvents with varying polarity (MeOD and 4:1 MeOD-D2O) further supported these notions (Supplementary Figures S20–S23).

**Figure 11:**
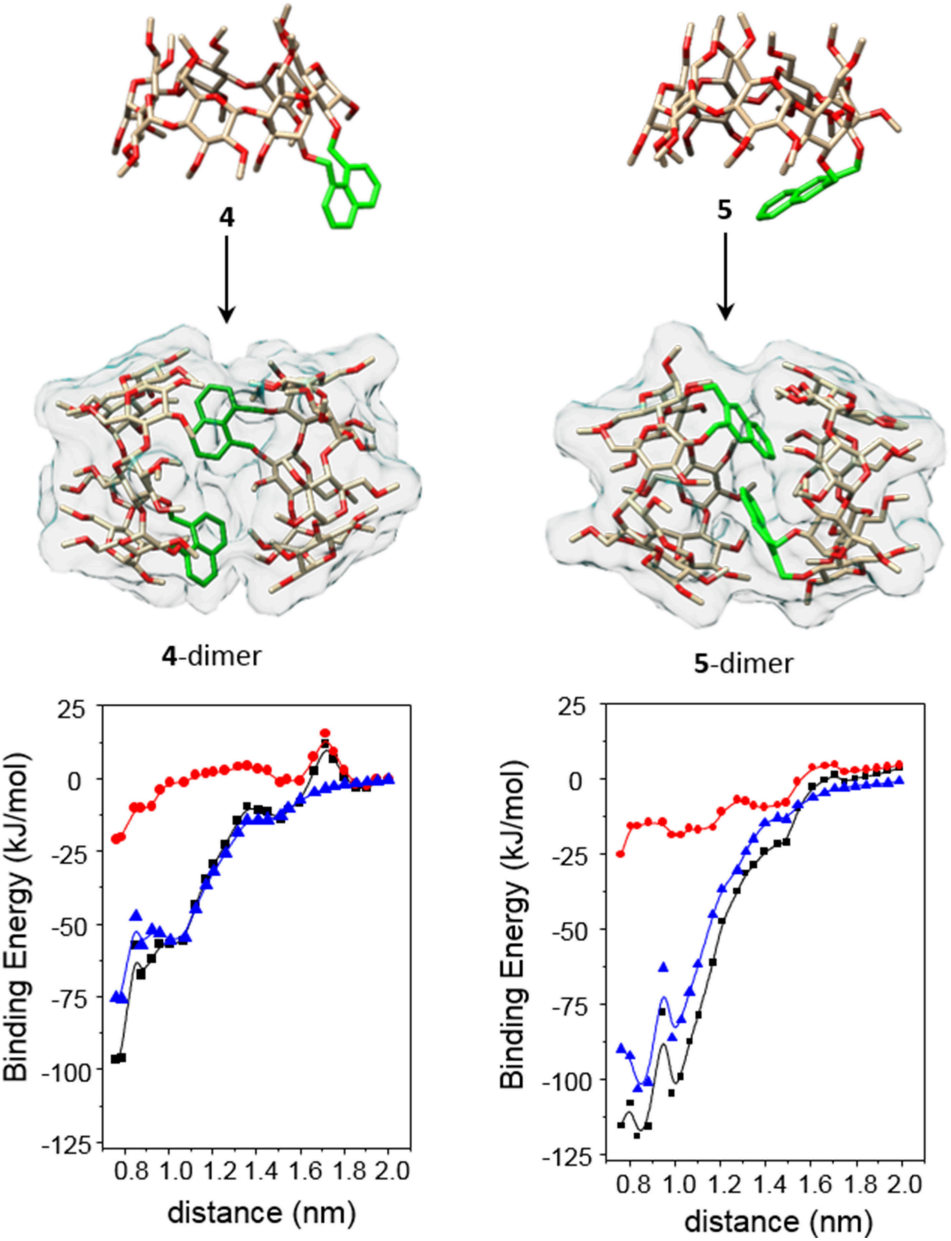
The **(Top** and **Middle)** represent 3D-views of the optimized (MM) structures of **4** and **5** (oxygen in red, carbon in tan; H atoms have been removed and the aromatic ring is highlighted in green for the sake of clarity) and minima binding energy (MBE) structures of the corresponding HH-dimers. The **(Bottom)** contains the changes of the total interaction energies (black) as well as the electrostatics (red) and van der Waals (blue) contributions, obtained after step by step approaching along the y axis defined by the center of mass of the glycosidic oxygens (see the Experimental section and the Supplementary Materials for details).

Molecular mechanics (MM) and molecular dynamics (MD) simulations for **4** and **5** reproduced the topological features inferred from the ICD experimental data (see Experimental section for a detailed description of the computational protocols). Thus, the probability distributions for the torsional angles, obtained from the analysis of the 2-ns MD trajectories in the vacuum at different temperatures (using the most stable structures calculated by MM as the starting conditions; Supplementary Figure S25), predominantly afforded near *fully-open* and *half-open* conformations for the monomeric forms of **4** and **5**, respectively (Figure 11, top panel). As expected, the ϕ_i_ and ψ_i_ angles for the bonds describing rotation about the bonds involving the glycosidic oxygens, C-1^i^–O and O— C-4^i+1^ (*i* = 1–7), adopted values typical for skewing states (0° ± 60°) from *trans* conformations in an undistorted βCD framework. Nevertheless, some ψ_i_ torsional angles could occasionally visit a *cis* state (Supplementary Figures S26, S27). The distributions of the four torsional angles χ describing the rotation around the bonds that link the appended aromatic moieties to the macroring (see Supplementary Figures S26, S27) hardly changed from their initial positions, indicating that the orientation of the naphthylene group remains confined in a relatively limited conformational space. The distributions of the distances between the center of mass of the naphthylene moiety and the βCD macroring for **4** and **5** (Supplementary Figure S28) at increasing temperatures further evidenced a significantly larger flapping amplitude for the latter, in agreement with the higher flexibility of the 2,3- vs. the 1,8-dimethylnaphthylene clip. MM data were also consistent with the preferred HH approaching of **4** or **5** upon self-assembly, yielding minimum binding energy (MBE) dimer aggregates featuring naphthylene—naphthylene distances fulfilling the requirements for the exciton coupling observed in the ICD spectra in water (Figure 11, middle panel). Van der Waals attractive interactions appeared as the most important contribution to the binding energy in both cases (Figure 11, bottom panel). The MBE structures were optimized again and used as starting structures for MD simulations. The corresponding 1-ns trajectories (Supplementary Figures S31, S32) next confirmed the stability of the dimer species. Most interestingly, the data support the initially theorized differences in the inner space topology of the assemblies: **4**-dimer exhibits an extended tubular cavity in which the 1,8-dimethylnaphthylene moieties behave as cavity extension modules whereas in **5**-dimer the 2,3-dimethylnaphthylene surfaces mutually act as partition elements between the two βCD cavities, resembling the situation previously encountered for the homologous *o*-xylylene derivative **2** (Neva et al., 2018).

### Inclusion Properties and the Impact in Dimer Stability

**Figure 12:**
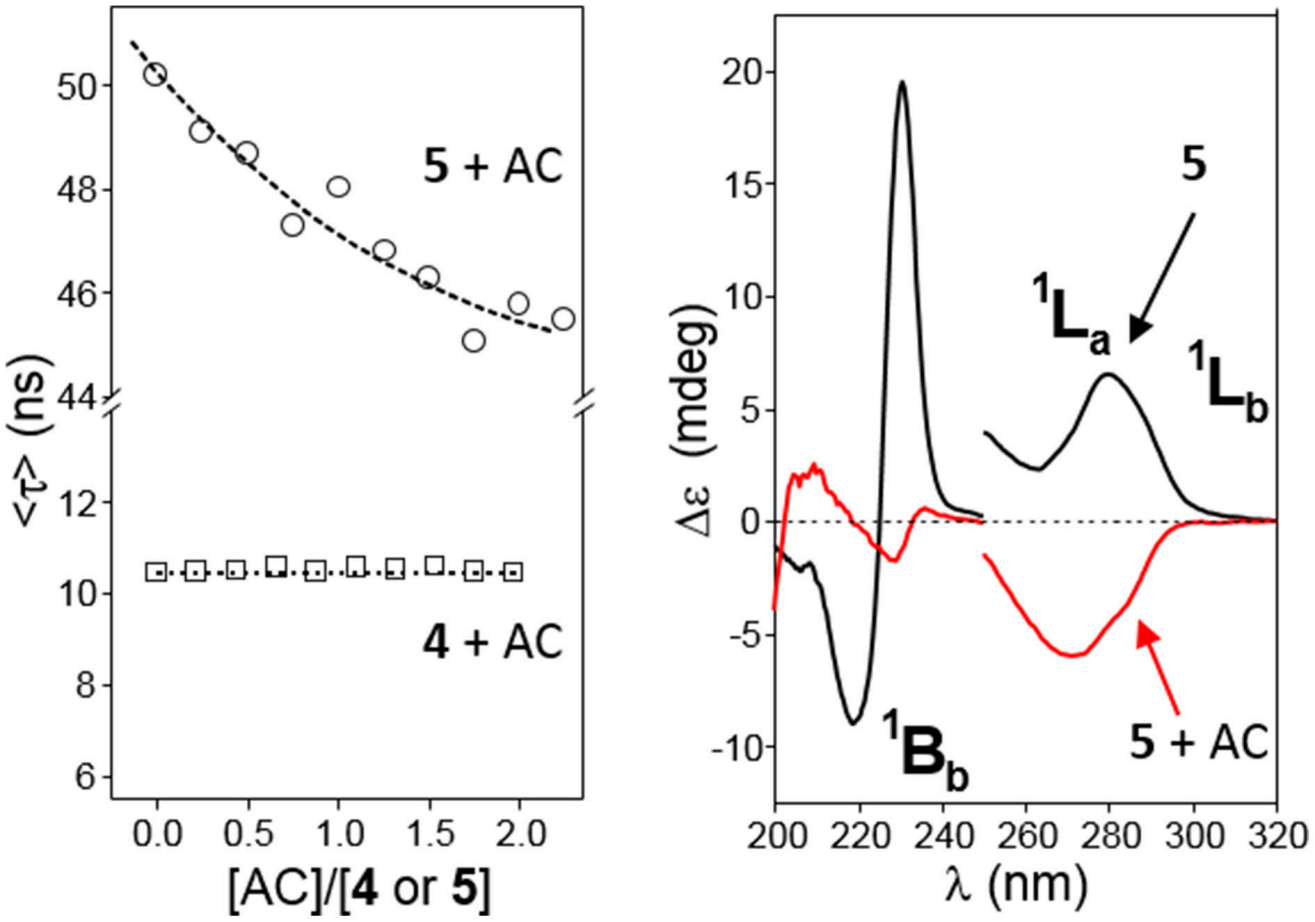
**(Left)** Plots of the average lifetime (<τ>) variations for water solutions of **4** (□) or **5** (◦, 1.36 · 10^−4^ M) at increasing AC concentrations at 25°C. **(Right)** Circular dichroism spectra of **5** (0.15 mM in water) in the absence (black line) and in the presence (red line) of a large excess of AC ([AC]/[**5**] = 86) at 25°C.

The revealed conformational differences between **4** and **5**, both in the monomer and dimer states, let presume that their abilities to form inclusion complexes in water solution will quite differ. It is interesting to speculate that a guest molecule fitting in the βCD cavity will probably have a stronger impact in the orientation of the relatively flexible 2,3-naphthylene segment in **5** as compared to the more rigid 1,8-naphthylene appendage in **4**, which at its turn would affect the corresponding monomer/dimer equilibrium. The close relationship between conformational properties and dimer stability offers then an opportunity for the spatiotemporal control of the aggregation/dissociation process through host-guest supramolecular chemistry. As a preliminary indication, the HRESI-mass spectrum of a water solution of **4** containing an excess of adamantane-1-carboxylate (AC), a preferred host for βCD derivatives that provides large association constants (Aoyagi et al., 1997; Kuwabara et al., 1999; Liu et al., 2004; Song et al., 2005), showed a relatively intense pseudomolecular peak at *m*/*z* 3331.5490, compatible with the AC guest trapped in the intact **4**-dimer, whereas the 1:1 **5**:AC complex (*m*/*z* 1777.8173) was preferred in the case of **5**, supporting that AC inclusion in the βCD cavity results in **5**-dimer disruption (Supplementary Figure S33). Fluorescence spectroscopy and circular dichroism further reinforced this notion. Thus, sequential addition of AC aliquots to a water solution of **4** (AC:**4** molar ratio from 0 to ~2) did not provoke significant changes in the average lifetime of the electronic excited states <τ> (Figure 12, left panel), whereas a similar experiment with **5** led to an important decrease in <τ>, meaning an increase in the proportion of the monomer (shorter lifetime) at the expenses of the dimer form. Analogously, the intensity of the ^1^B_b_, ^1^L_a_, and ^1^L_b_ bands in the corresponding ICD spectra of **4** were essentially unaltered in the presence of AC, whereas in the case of **5** monotonically decreased with AC concentration (Supplementary Figures S34, S35). Notably, adding a large excess of AC to **5** in water additionally triggered a drastic change in the sign of the ^1^L_a_ band from positive to negative, matching the effect observed upon decreasing the solvent polarity (Figure 12, right panel, and Supplementary Figure S36). This result implies that AC inclusion in the cavity of **5** leads to a reorientation of the 2,3-dimethylnaphthylene clip to meet the disposition encountered in the monomer, strongly supporting a guest-promoted conformational switch mechanism at the origin of the AC-induced dimer dissociation.

^1^H NMR titration experiments in buffered aqueous media at 25°C confirmed the formation of supramolecular complexes between AC and **4** or **5**. In agreement with fluorescence and ICD data, the chemical shift variations of selected proton resonances of **4** (0.5 mM, meaning a **4**-dimer molar fraction > 90%) induced by increasing proportions of AC afforded binding isotherms that fitted well to a 1:1 host:guest binding model between **4**-dimer and the adamantane carboxylate guest, i.e., a 2:1 stoichiometry based on **4**-monomer (Supplementary Figure S37). Least square fitting yielded an apparent association constant *K*_a_ = 1.3 ± 0.2 · 10^4^ M^−1^ for the equilibrium **4**-dimer + AC ⇆ **4**-dimer:AC. In the case of compound **5**, a similar analysis returned an apparent *K*_a_ = 1.1 ± 0.4 · 10^3^ M^−1^ for a 1:1 complex between the monomer form of **5** and the AC guest, although this value is probably underestimated given that formation of the **5**:AC inclusion complex requires **5**-dimer disassembly (Supplementary Figure S38). For comparison, the *K*_a_ value for the 1:1 inclusion complexes of AC with permethylated βCD, which does not form dimers in solution, or with the *o*-xylylene derivative **2**, which forms an HH-dimer in water solution that dissociates upon inclusion complex formation, were 1262 ± 130 M^−1^ and 5489 ± 1353 M^−1^, respectively (Neva et al., 2018).

**Figure 13:**
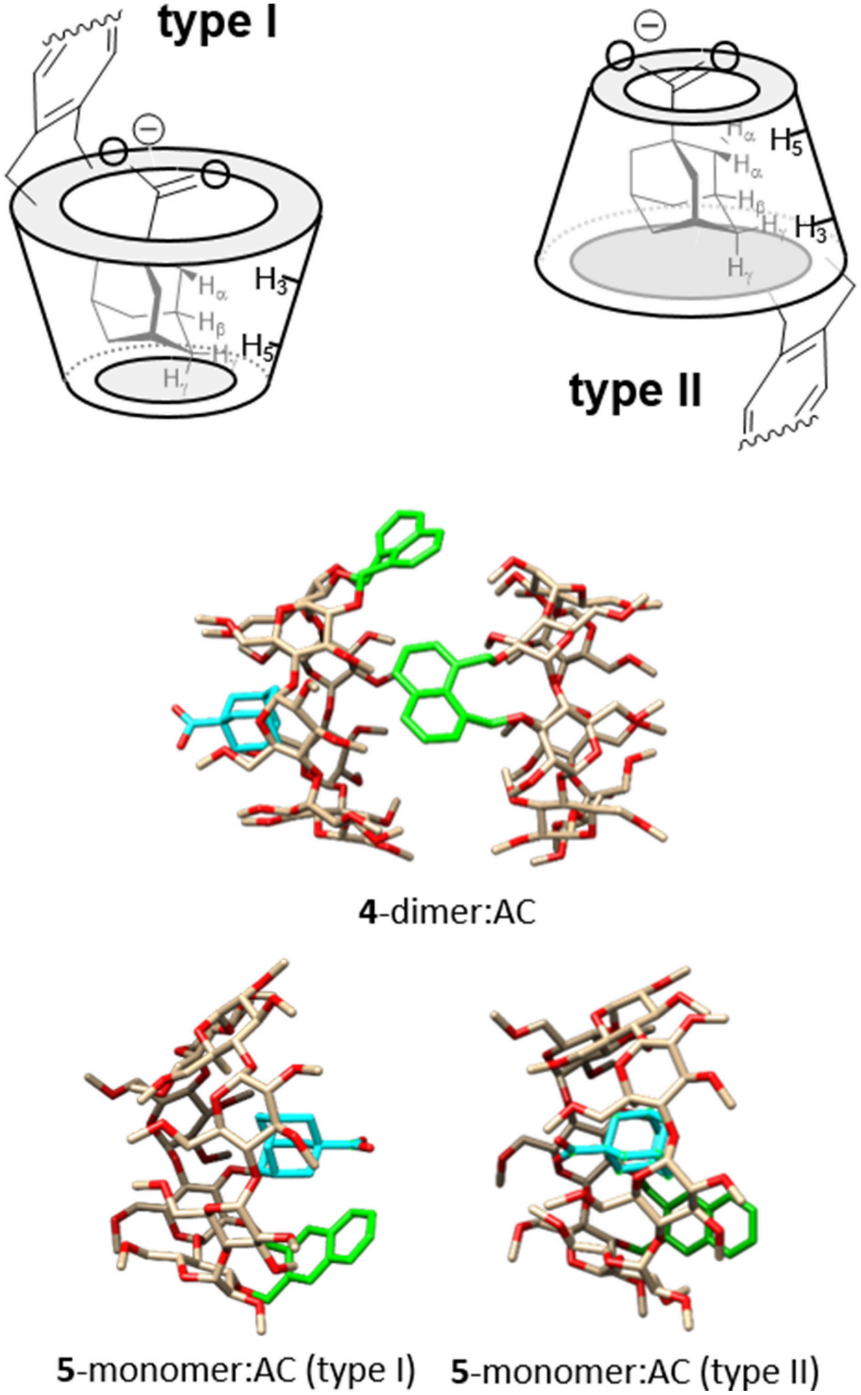
**(Top)** Schematic representation of type I and type II complexes between AC and βCD derivatives bearing aromatic clips, with indication of the expected diagnostic NOE contacts. **(Middle)** MBE structure of the ternary complex formed by **4**-dimer and AC. **(Bottom)** MBE structures of the type I (left) and type II (right) **5**-dimer:AC complexes obtained by approaching the AC guest to the CD host by the primary rim, to give a type I complex (left), or by the secondary rim, to give a type II complex (right). In the CD partners, oxygens are colored in red, carbons in tan; H atoms have been removed and the aromatic ring is highlighted in green; in the AC partner, the carbon atoms are highlighted in cyan for the sake of clarity.

Despite of the huge amount of information on the complexes between βCD and adamantane derivatives accumulated over more than 50 years, the orientation of the guest in the cyclooligosaccharide cavity continues to be a subject of debate. Negatively charged hydrophilic substituents, such as the carboxylate group in AC, are not included in the hydrophobic receptacle, but it is unclear whether they protrude from the wider (type I) or the narrower rim (type II complex) (Rüdiger et al., 1996; Gómez-Biagi et al., 2008; Krishnan et al., 2012). A recent study unequivocally demonstrates that both types of complexes coexist in approximately equal amounts in the case of the inclusion complex between AC and per-*O*-methylated βCD (Schönbeck, 2018), but the preferred guest orientation may well-depend on the modification of the βCD platform. A comparative analysis of NOE (or ROE) contacts between the α, β and γ protons in AC and the inside located H-3 and H-5 CD protons provides an indication on this issue (Figure 13, top panel). In our case, the asymmetric nature of the macroring in both **4** and **5** and the extensive signal overlapping precludes a precise quantification. Yet, a qualitative assessment of the NOESY spectra for the corresponding AC complexes was consistent with a largely predominant type II arrangement for the 1,8-dimethylnaphthylene derivative **4** (strong H-5/H-α and H-3/H-γ contacts, Supplementary Figure S39) and the presence of a much higher proportion of type I complex in the case of the 2,3-dimethylnaphthylene positional isomer **5** (H-3/H-α and H-5/H-γ spatial correlations were additionally present). In principle, access to the βCD cavity through the secondary rim is prevented in the dimers, guiding AC entrance through the primary face to give a type II orientation. In the case of **4**-dimer, the resulting ternary species remains stable (Figure 13, middle panel). In contrast, AC inclusion in **5**-dimer probably weakens the intermolecular naphthalene-naphthalene interactions by affecting the orientation of the aromatic clip (note that NOE contacts between aromatic and adamantane protons are also detected; Supplementary Figure S40), leading to dissociation into a 1:1 **5**-monomer:AC complex and a free host molecule. Inclusion through the secondary face becomes thus feasible, enabling equilibration between type I and type II **5**:AC complexes (Figure 13, bottom panel).

## Conclusion

We successfully synthesized two naphthylene-clipped β-cyclodextrin regioisomers with the ability to self-assemble into head-to-head supramolecular dimers in water solution, the aromatic appendage being either 1,8- (**4**) or 2,3-doubly-linked (**5**) to consecutive *O*-2^I^ and *O*-3^II^ positions in the secondary rim of the CD core through benzyl ether-type bridges. Such structural alteration translated into significant differences in the topological properties and stability of the dimer species as supported by MS, NMR, fluorescence and circular dichroism. Thus, **4**-dimer adopts a very stable tubular-like arrangement in which the aromatic walls, in an open conformation, essentially extent the CD secondary faces of the monomer components leaving the primary rims exposed, whilst in **5**-dimer the naphthalene platforms enjoy higher flexibility and take quasi-capped dispositions, acting as folding screens that separate the individual CD units. These conformational and architectural dissimilarities further condition the inclusion properties: **4**-dimer can host the adamantane carboxylate guest to form a **4**-dimer:AC stable ternary species whereas the same AC guest triggers **5**-dimer disruption. Interestingly, the synthetic approach is compatible with the incorporation of functional elements at the primary positions of the cyclooligosaccharide, e.g., cationic centers to modulate the reactivity of guests confined in the CD cavity (Wei et al., 2018; Yi et al., 2018) or to mediate nucleic acid condensation (Aranda et al., 2013; Martínez et al., 2013; Jiménez Blanco et al., 2016; Martínez-Negro et al., 2017; Przybylski et al., 2018). The possibility of implementing different supramolecular organization levels (e.g., clusterization and/or inclusion and/or spatiotemporal controlled dissociation) with aromatic clips offers then a valuable tool to program the behavior of monodisperse CD-based devices in biological environments for (bio)molecular encapsulation and controlled release. It is worth mentioning that, although AC was just a model guest in this study, the adamantane moiety is present in many drugs (Wanka et al., 2013; de la Mata et al., 2015; Spilovska et al., 2016) and delivery systems (Rodríguez-Lavado et al., 2014; Štimac et al., 2017) that could be used in combination with customized analogs of **4** or **5** for biomedically oriented applications. Work in this direction is currently sought in our laboratories.

## Author Contributions

All authors listed have made a substantial, direct and intellectual contribution to the work, and approved it for publication.

### Conflict of Interest Statement

The authors declare that the research was conducted in the absence of any commercial or financial relationships that could be construed as a potential conflict of interest.
