# Supplementary material for: Dynamic Control of the Self-Assembling Properties of Cyclodextrins by the Interplay of Aromatic and Host-Guest Interactions
Source: Front Chem. 2019 Feb 25;7:72. doi: 10.3389/fchem.2019.00072 (PMC6401617; doi:10.3389/fchem.2019.00072)
Supplement: Supplementary file 1 [file Data_Sheet_1.pdf]

Supplementary Material

Dynamic Control of the Self-Assembling Properties of Cyclodextrins by the Interplay of Aromatic and Host-Guest Interactions

Tania Neva[1†], Thais Carmona[2†], Juan M. Benito[1], Cédric Przybylski,[3] Carmen Ortiz Mellet[4]*, Francisco Mendicuti[2]* and José M. García Fernández[1]*

*** Correspondence:**

Carmen Ortiz Mellet: mellet@us.es

Francisco Mendicuti: francisco.mendicuti@uah.es

José M. García Fernández: jogarcia@iiq.csic.es

1 Supplementary Data: Synthesis and Compound Characterization

2^{I},3^{II}-*O*-(1,8-Dimethylnaphthalene)-$2^{II\text{-}VII}$,$3^{I,III\text{-}VII}$,$6^{I\text{-}VII}$-nonadeca-*O*-methyl-cyclomaltoheptaose (**4**). A solution of freshly lyophilized **7** (0.39 g, 0.27 mmol) in dry DMF (6 mL) under N_2 was added to a suspension of NaH (40 mg, 1.66 mmol, 3 eq) in DMF (4 mL) under N_2 and the mixture was stirred at RT for 30 min. Then, a solution of 1,8-dibromomethyl-naphthalene (131 mg, 0.42 mmol, 1.5 eq) in dry DMF (2 mL) was added and the reaction mixture was stirred under N_2 atmosphere at RT for 12 h. Then, the reaction was quenched by addition of 1 m HCl (1.5 mL), concentrated to dryness. The resulting residue was taken in DCM (10 mL) and washed with water (10 mL). The organic layer was separated and dried with Na_2SO_4, the solvent was removed under reduced pressure and the resulting syrup was purified by column chromatography using 100:1 → 20:1 EtOAc/EtOH as eluent. Yield 234 mg (56%). R_f 0.22 (20:1 EtOAc/EtOH). $[\alpha]_D$ + 129.5 (*c* 1.0, $CHCl_3$). ^{1}H NMR (600 MHz, MeOD, Figure S1): δ 7.94 (2 dd, 2 H, $^3J_{H,H}$ 8.1 Hz, $^4J_{H,H}$ 1.1 Hz, $Naphth_{H\text{-}4,5}$), 7.67 (dd, 1 H, $^3J_{H,H}$ 7.2 Hz, $Naphth_{H\text{-}7}$), 7.63 (dd, 1 H, $^3J_{H,H}$ 7.1 Hz, $Naphth_{H\text{-}2}$), 7.50 (dd, 1 H, $Naphth_{H\text{-}6}$), 7.49 (dd, 1 H, $Naphth_{H\text{-}3}$), 6.45, 4.63 (2d, 2 H, $J_{H,H}$ 9.1 Hz, CH_2-$O3^{II}$), 5.84, 4.53 (2d, 2 H, $J_{H,H}$ 9.3 Hz, CH_2-$O2^{I}$), 5.36 (d, 1 H, $J_{1,2}$ 4.1 Hz, H-1^{I}), 5.22, 5.19, 5.18, 5.17 (5 d, 5 H, $J_{1,2}$ 3.8-4.1 Hz, H-$1^{III\text{-}VII}$), 5.17 (d, 1 H, $J_{1,2}$ 3.7 Hz, H-1^{II}), 4.03 (dd, 1 H, $J_{5,6a}$ 4.5 Hz, $J_{6a,6b}$ 10.7 Hz, H-$6a^{II}$), 3.96 (ddd, $J_{4,5}$ 9.6 Hz, $J_{5,6b}$ 1.2 Hz, H-5^{II}), 3.93-3.83 (m, 10 H, H-$5^{III\text{-}VII}$, H-$6a^{III\text{-}VII}$), 3.90 (m, 2 H, H-5^{I}, H-$6a^{I}$), 3.90 (t, 1 H, $J_{2,3}$ = $J_{3,4}$ 9.6 Hz, H-3^{II}), 3.85 (t, 1 H, H-4^{II}), 3.76 (dd, 1 H, H-$6b^{II}$), 3.75 (dd, 1 H, $J_{2,3}$ 9.4 Hz, H-2^{I}), 3.74, 3.68, 3.67, 3.65, 3.63 (5 s, 15 H, $O3^{III\text{-}VII}$-Me), 3.71 (bd, 1 H, $J_{6a,6b}$ 9.6 Hz, H-$6b^{I}$), 3.63 (m, 2 H, H-3^{I}, H-4^{I}), 3.57 (s, 3 H, $O2^{II}$-Me), 3.66-3.50 (m, 10 H, H-$3^{III\text{-}VII}$, H-$4^{III\text{-}VII}$), 3.56, 3.54, 3.53, 3.52, 3.49 (5 s, 15 H, $O2^{III,VII}$-Me), 3.48 (s, 3 H, $O3^{I}$-Me), 3.43 (s, 3 H, $O6^{II}$-Me), 3.41-3.40 (6 s, 18 H, $O6^{I,III\text{-}VII}$-Me), 3.21 (dd, 1 H, H-2^{II}), 3.22, 3.20-3.15 (5 dd, 5 H, H-$2^{III\text{-}VII}$). ^{13}C NMR (100.6 MHz, MeOD, Figure S7): δ 135.6 ($Naphth_{C\text{-}8}$), 133.8, 133.4, 133.3, 132.9 ($Naphth_{C\text{-}1,2,7,9,10}$), 132.8, 130.4 ($Naphth_{C\text{-}4,5}$), 124.9, 124.8 ($Naphth_{C\text{-}3,6}$), 100.5 (C-$1^{I}$), 98.4 (C-$1^{II}$), 98.4, 98.0-97.9 (C-$1^{III\text{-}VII}$), 83.8 (C-$4^{II}$), 82.2-81.7 (C-$2^{III\text{-}VII}$, C-$4^{I,III\text{-}VII}$), 81.1 (C-$3^{II}$), 81.0 (C-$2^{II}$), 80.1 (C-$2^{I}$), 79.6 (C-$3^{I}$), 79.2-79.0 (C-$3^{III\text{-}VII}$), 77.0 (Naphth*C*$H_2$-$O3^{II}$), 71.6-71.4 (C-$6^{I,III\text{-}VII}$), 71.4 (C-$6^{II}$), 71.1 Naphth*C*$H_2$-$O2^{I}$), 71.3, 71.0-70.8 (C-$5^{III\text{-}VII}$), 71.0 (C-$5^{II}$), 70.7 (C-$5^{I}$), 60.7 ($O3^{I}$-Me), 60.4-60.3 ($O3^{III\text{-}VII}$-Me), 58.6 ($O2^{II}$-

Me), 58.0-57.6 (O2$^{III-VII}$-Me, O6^{I-VII}-Me). ESI-MS *m/z* 1575.8 [M + Na]$^{+}$, 799.3 [M + 2Na]$^{2+}$. Elem. Anal. Calcd for $C_{73}H_{116}O_{35}$: C, 56.43; H, 7.53; found C, 56.53; H, 7.62.

2^{I},3^{II}-*O*-(2,3-Dimethylnaphthalene)-2^{II-VII},3$^{I,III-VII}$,6^{I-VII}-nonadeca-*O*-methyl-cyclomaltoheptaose (**5**). A solution of freshly lyophilized **7** (0.38 g, 0.27 mmol) in dry DMF (6 mL) under N_2 was added to a suspension of NaH (39 mg, 1.6 mmol, 3 eq) in dry DMF (4 mL) under N_2 and the mixture was stirred at RT for 30 min. Then, a solution of 2,3-dibromomethyl-naphthalene (127 mg, 0.40 mmol, 1.5 eq) in dry DMF (2 mL) was added and the reaction mixture was stirred under N_2 atmosphere at RT for 12 h. Then, the reaction was quenched by addition of 1M HCl (1.5 mL), concentrated to dryness. The resulting residue was taken in DCM (10 mL) and washed with water (10 mL). The organic layer was separated and dried with Na_2SO_4, the solvent was removed under reduced pressure and the resulting syrup was purified by column chromatography using 100:1→20:1 EtOAc/EtOH as eluent. Yield 200 mg (48%). R_f 0.24 (20:1 EtOAc/EtOH). $[\alpha]_D$ + 10.9 (*c* 1.0, $CHCl_3$). ^{1}H NMR (600 MHz, MeOD, Figure S9): δ 7.87 (m, 2 H, Naphth$_{H-5,8}$), 7.79 (s, 1 H, Naphth$_{H-1}$), 7.78 (s, 1 H, Naphth$_{H-4}$), 7.51 (m, 2 H, Naphth$_{H-6,7}$), 5.44, 4.90 (2d, 2 H, $^{2}J_{H,H}$ 9.0 Hz, CH_2-O3^{II}), 5.32, 4.64 (2d, 2 H, $^{2}J_{H,H}$ 10.0 Hz, CH_2-O2^{I}), 5.32 (d, 1 H, $J_{1,2}$ 4.0 Hz, H-1^{I}), 5.19, 5.17, 5.15 (4 d, 4 H, $J_{1,2}$ 3.5-3.7 Hz, H-1$^{III-VII}$), 5.14 (d, 1 H, $J_{1,2}$ 3.5 Hz, H-1^{II}), 3.99-3.82 (m, 10 H, H-5$^{III-VII}$, H-6a$^{III-VII}$), 3.94 (ddd, $J_{4,5}$ 9.9 Hz, $J_{5,6a}$ 4.8 Hz, $J_{5,6b}$ 1.7 Hz, H-5^{II}), 3.92 (dd, $J_{6a,6b}$ 10.7 Hz, 1 H, $J_{5,6a}$ 4.5 Hz, H-6a^{I}), 3.83 (ddd, 1 H, $J_{4,5}$ 8.8 Hz, $J_{5,6b}$ 1.3 Hz, H-5^{I}), 3.83 (t, 1 H, $J_{2,3}$ 9.6 Hz, $J_{3,4}$ 8.6 Hz, H-3^{II}), 3.77 (dd, 1 H, $J_{6a,6b}$ 10.8 Hz, H-6a^{II}), 3.73, 3.68, 3.65, 3.64 (5 s, 15 H, O3$^{III-VII}$-Me), 3.72 (dd, 1 H, H-6b^{II}), 3.68-3.51 (m, 10 H, H-3$^{III-VII}$, H-4$^{III-VII}$), 3.67 (dd, 1 H, H-6b^{I}), 3.61 (dd, 1 H, $J_{3,4}$ 9.7 Hz, H-4^{I}), 3.60 (dd, 1 H, H-4^{II}), 3.59, 3.58, 3.56, 3.54, 3.52-3.51 (7 s, 21 H, O2^{II-VII}-Me, O3^{I}-Me), 3.53 (t, 1 H, $J_{2,3}$ 9.7 Hz, H-3^{I}), 3.48 (dd, 1 H, H-2^{I}), 3.41, 3.40, 3.39 (7 s, 21 H, O6^{I-VII}-Me), 3.23 (dd, 1 H, H-2^{II}), 3.20-3.15 (5 dd, 5 H, H-2$^{III-VII}$). ^{13}C NMR (100.6 MHz, MeOD, Figure S15): δ 135.0, 134.9 (Naphth$_{C-2,3}$), 133.0, 133.1 (Naphth$_{C-9,10}$), 129.5 (Naphth$_{C-4}$), 128.7 (Naphth$_{C-1}$), 127.2 (Naphth$_{C-5,8}$), 126.1, 126.0 (Naphth$_{C-6,7}$), 99.5 (C-1^{I}), 98.7, 98.4, 98.1-97.9 (C-1^{II-VII}), 82.2-81.7 (C-2^{II-VII}, C-4^{I-VII}), 80.9 (C-3^{II}), 80.5, 80.0, 79.7, 79.3, 79.2, 78.6 (C-3$^{I,III-VII}$), 79.1 (C-2^{I}), 75.7 (Naphth*C*H_2-O3^{II}), 71.5-70.6 (Naphth*C*H_2-O2^{I}, C-5^{I-VII}, C-6^{I-VII}), 60.9-60.3 (O3$^{I,III-VII}$-Me), 58.0-57.5 (O2^{II-VII}-Me, O6^{I-VII}-Me). ESI-MS *m/z* 1575.8 [M + Na]$^{+}$, 799.3 [M + 2Na]$^{2+}$. Elem. Anal. Calcd for $C_{73}H_{116}O_{35}$: C, 56.43; H, 7.53; found C, 56.12; H, 7.26.

2 Supplementary Figures and Tables

For more information on Supplementary Material and for details on the different file types accepted, please see here. Figures, tables, and images will be published under a Creative Commons CC-BY licence and permission must be obtained for use of copyrighted material from other sources (including re-published/adapted/modified/partial figures and images from the internet). It is the responsibility of the authors to acquire the licenses, to follow any citation instructions requested by third-party rights holders, and cover any supplementary charges.

2.1 Supplementary Figures

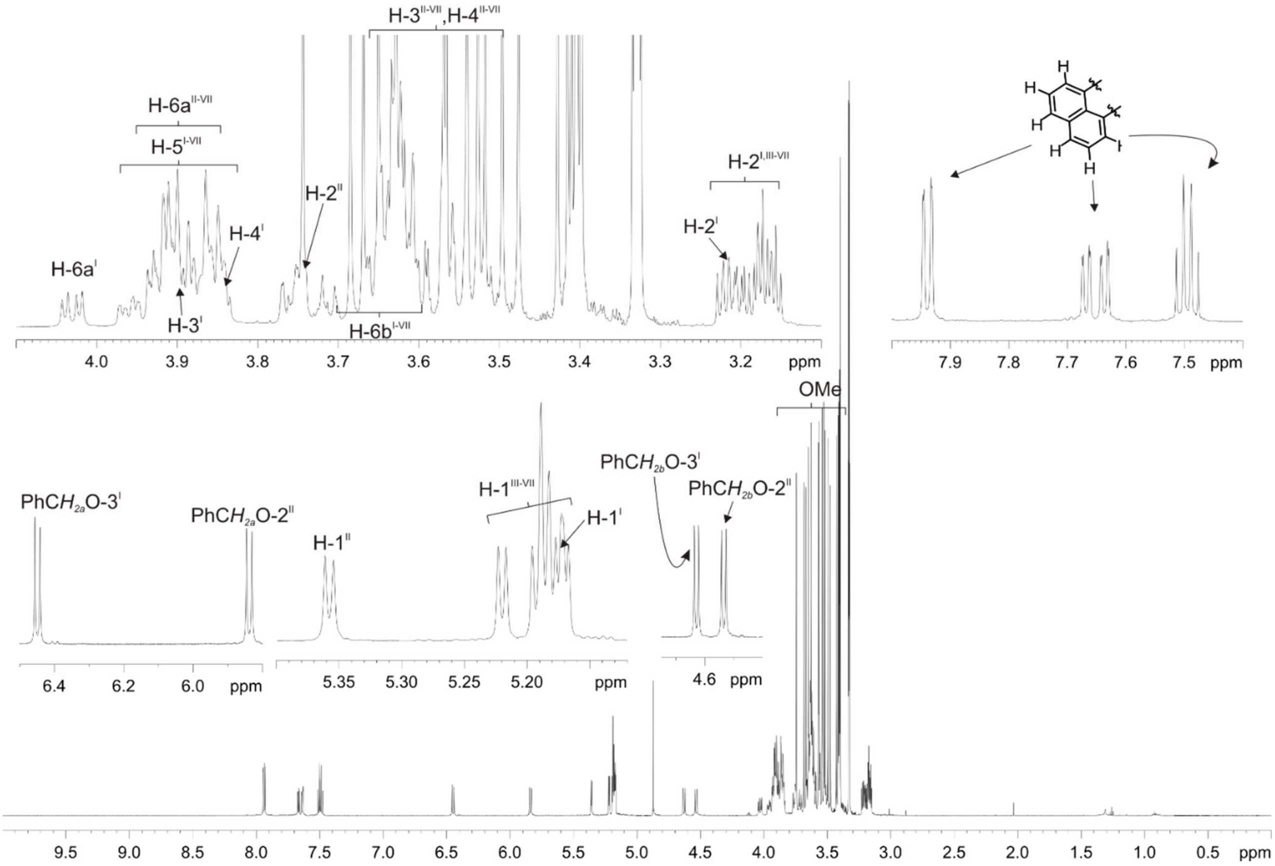

Supplementary Figure S1. ^{1}H NMR (600 MHz, MeOD) spectrum of compound **4** (3 mM).

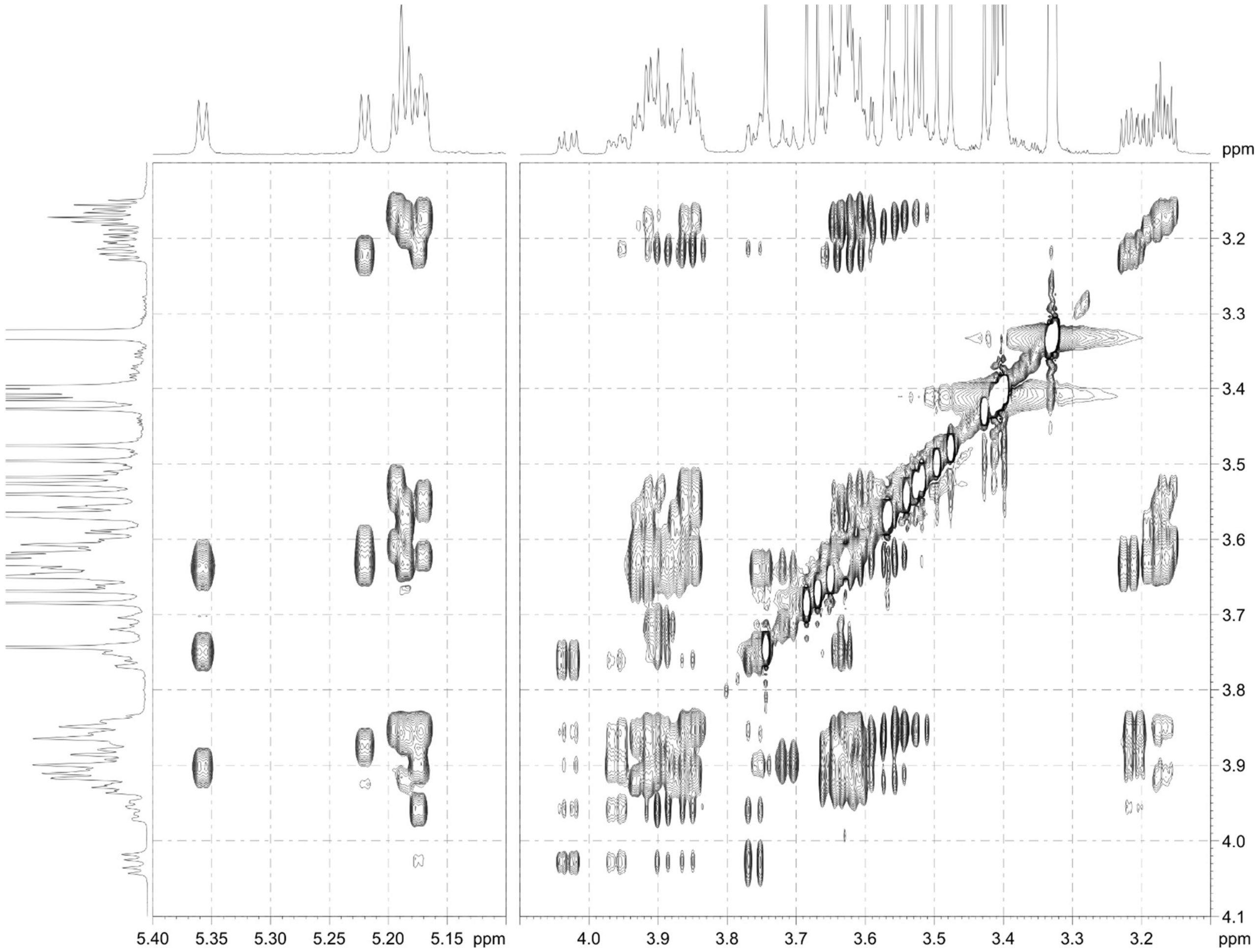

Supplementary Figure S2. Selected regions of the 2D TOCSY (600 MHz, MeOD) spectrum of compound **4** (3 mM).

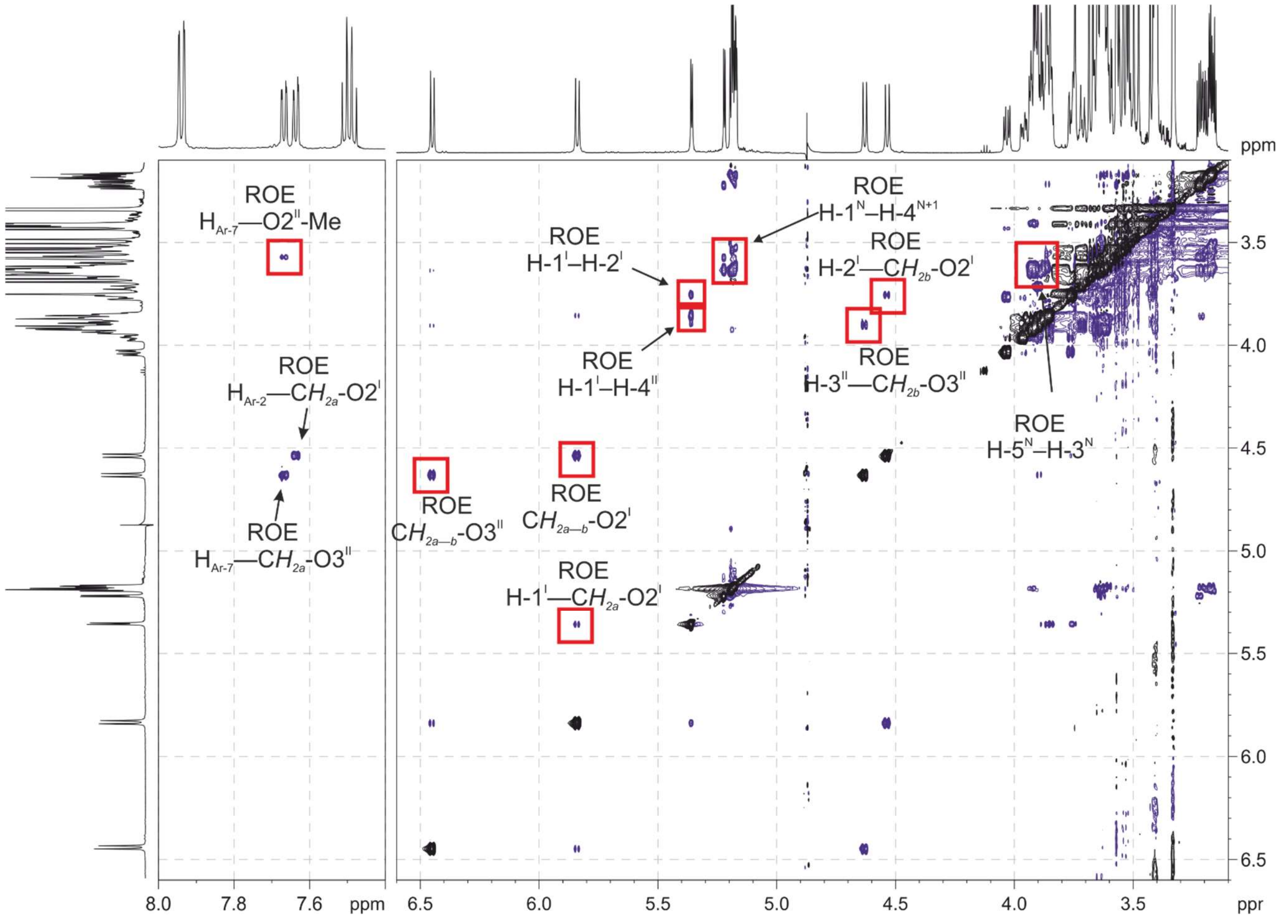

Supplementary Figure S3. Selected regions of the 2D ROESY (600 MHz, MeOD) spectrum of compound **4** (3 mM).

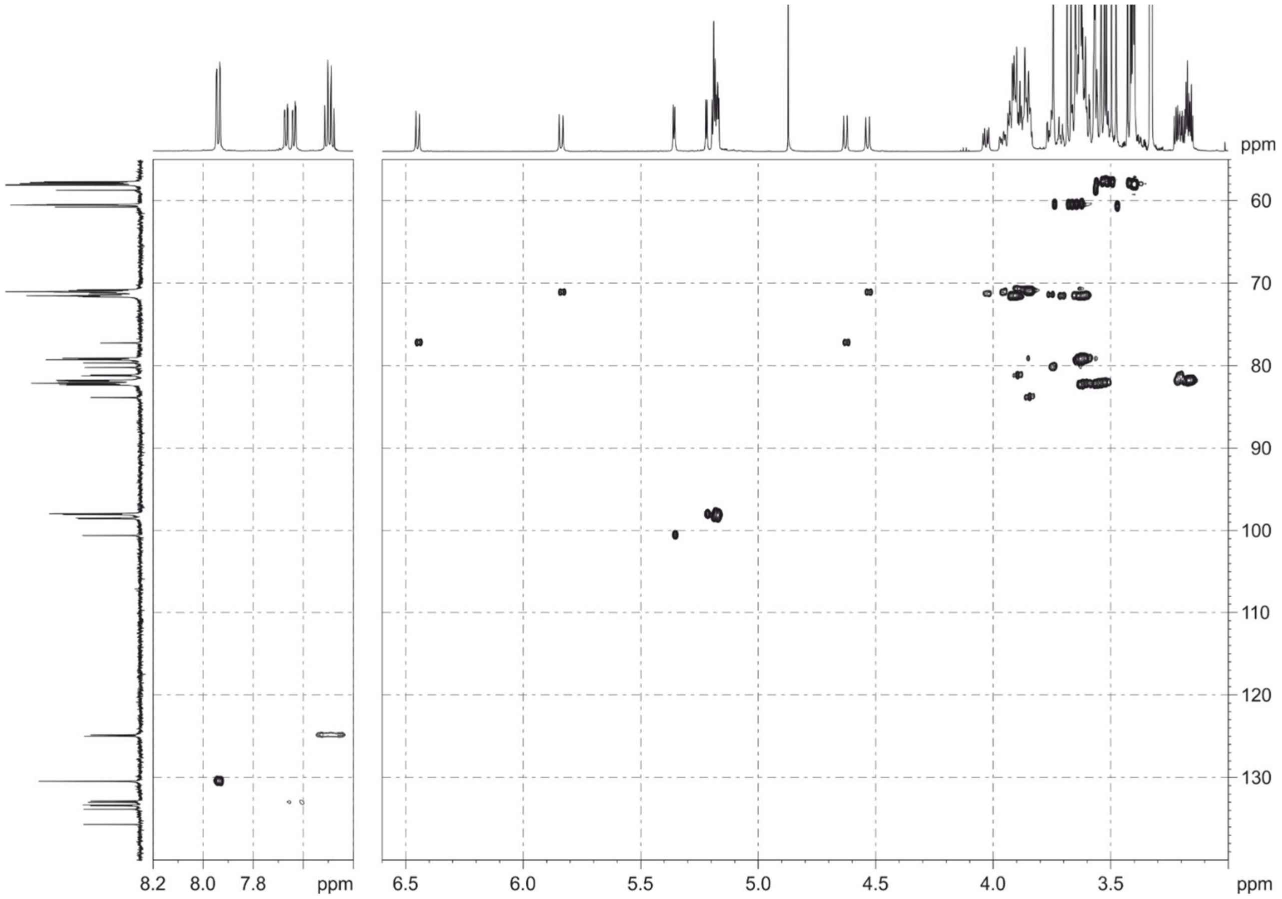

Supplementary Figure S4. HSQC (600 MHz, MeOD) spectrum of compound **4** (3 mM).

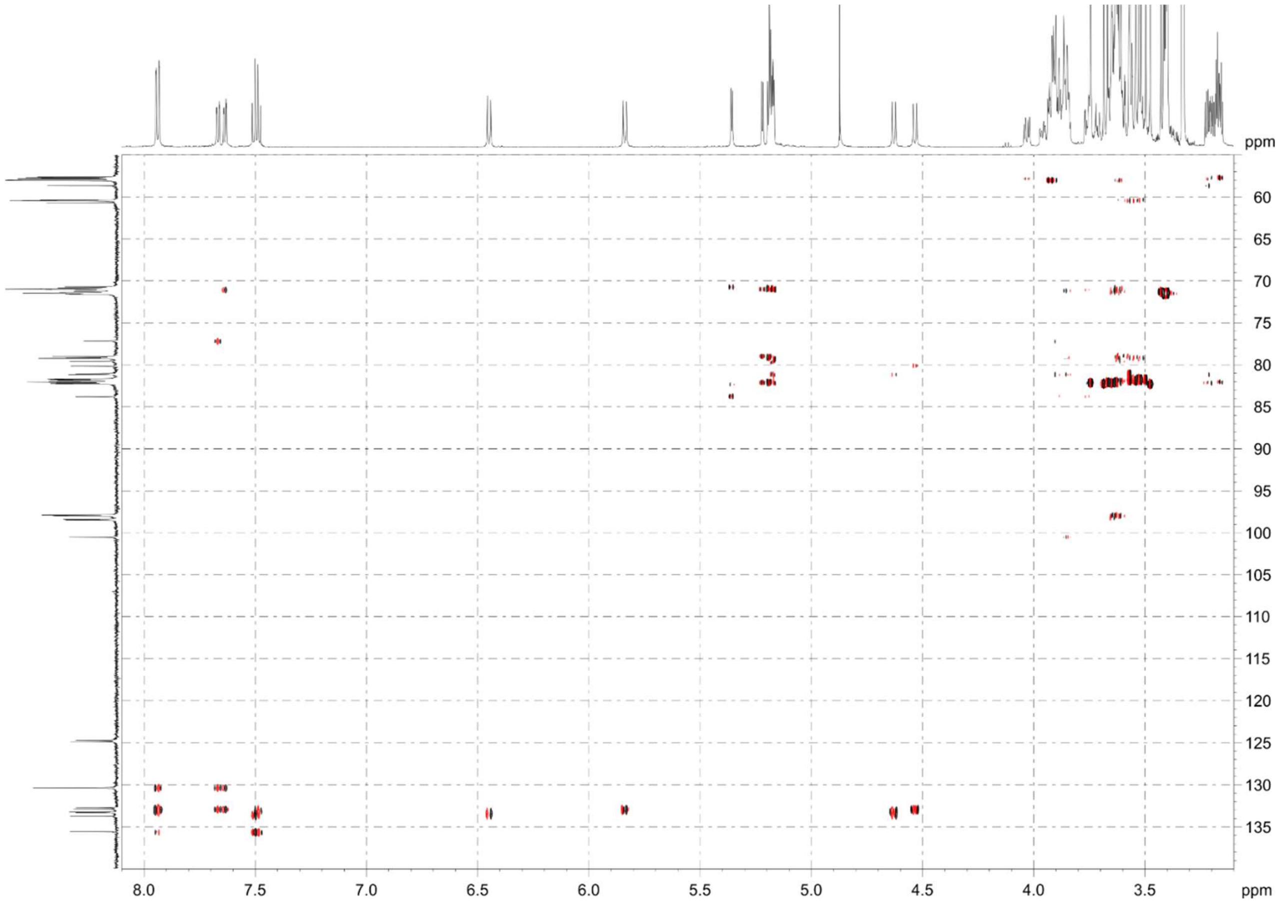

Supplementary Figure S5. HMBC (600 MHz, MeOD) spectrum of compound **4** (3 mM).

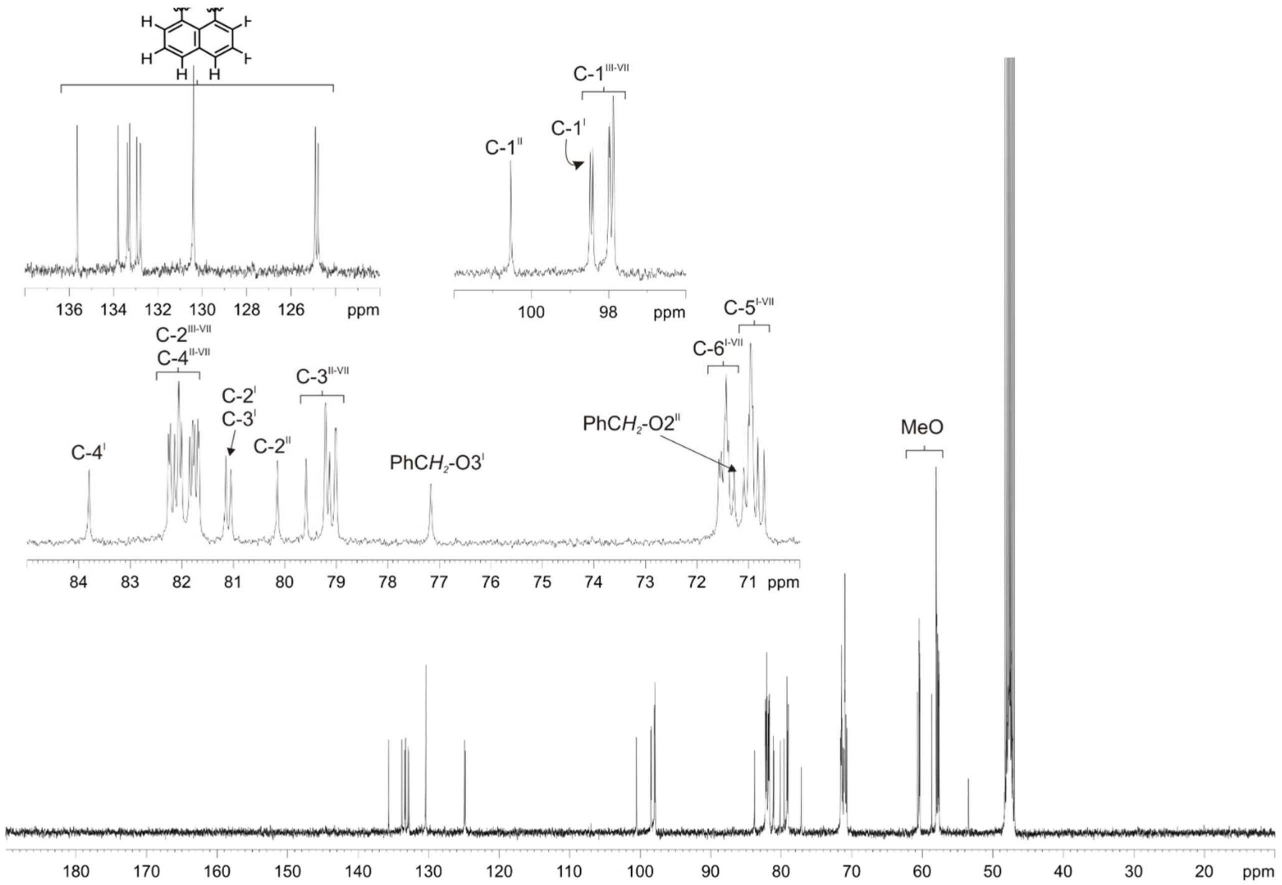

Supplementary Figure S6. ^{13}C NMR (100.6 MHz, MeOD) spectrum of compound **4** (3 mM).

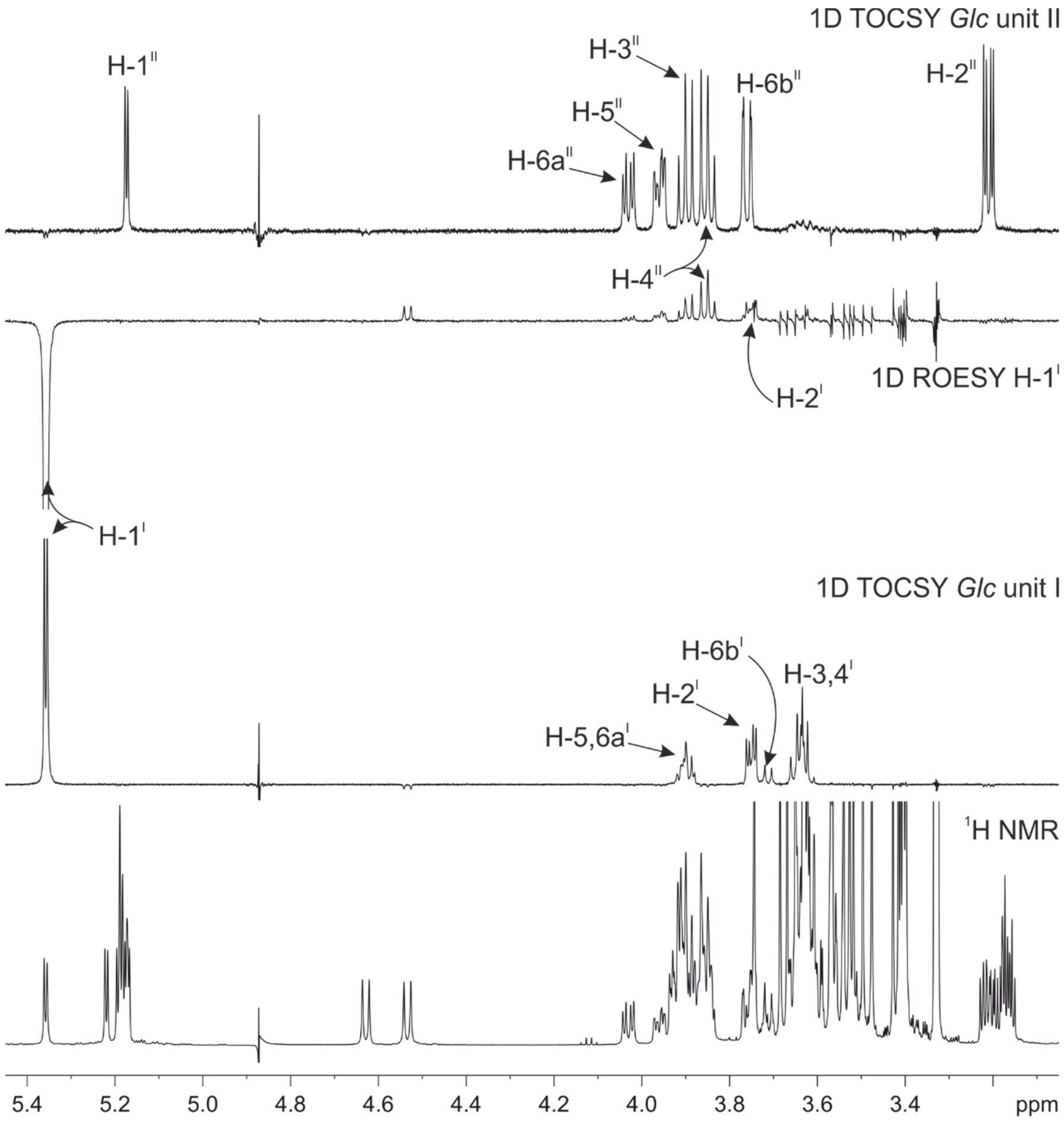

Supplementary Figure S7. Stacked ^{1}H, 1D TOCSY of glucose units I and II, and inter-glucoside I-II 1D ROESY (600 MHz, MeOD) spectra of compound **4** (3 mM).

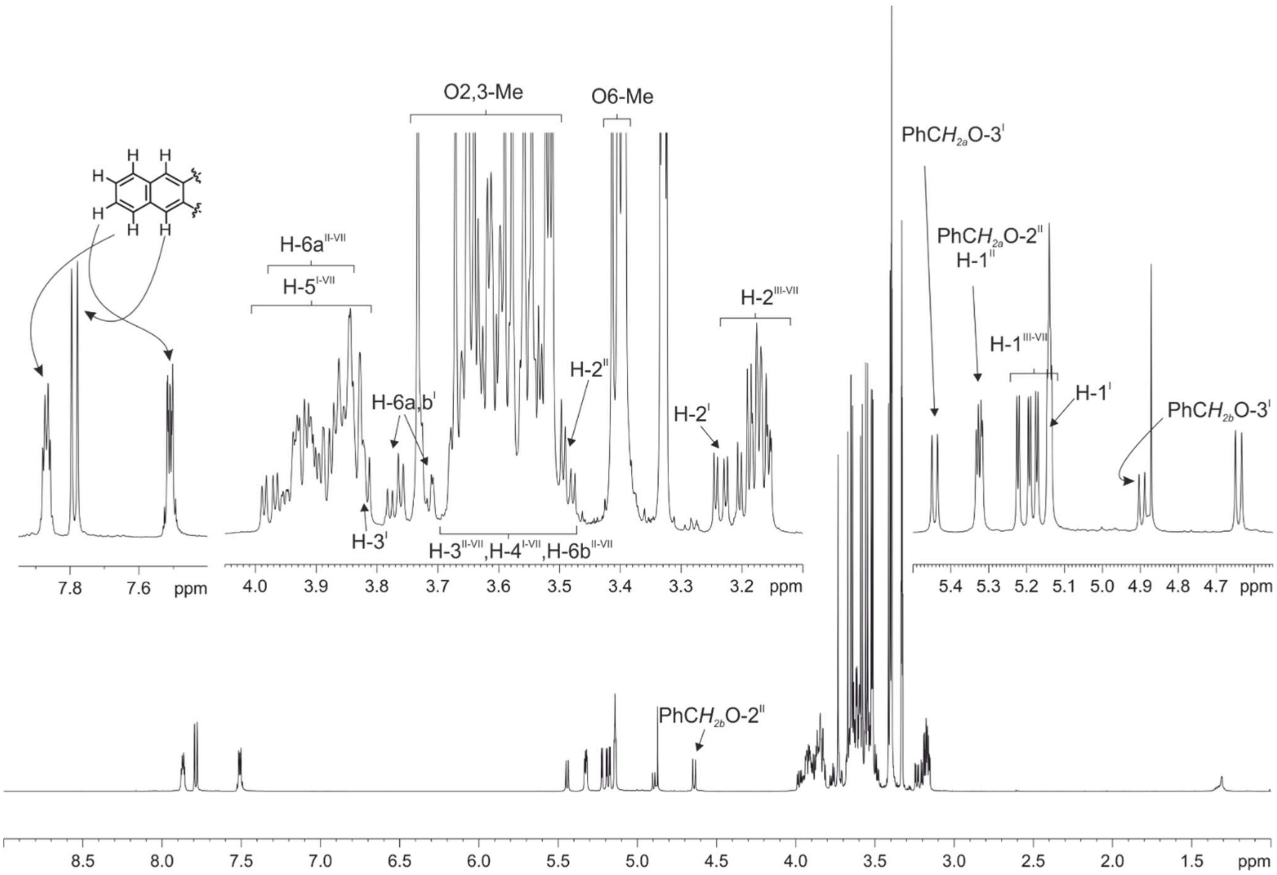

Supplementary Figure S8. ^{1}H NMR (600 MHz, MeOD) spectrum of compound **5** (3 mM).

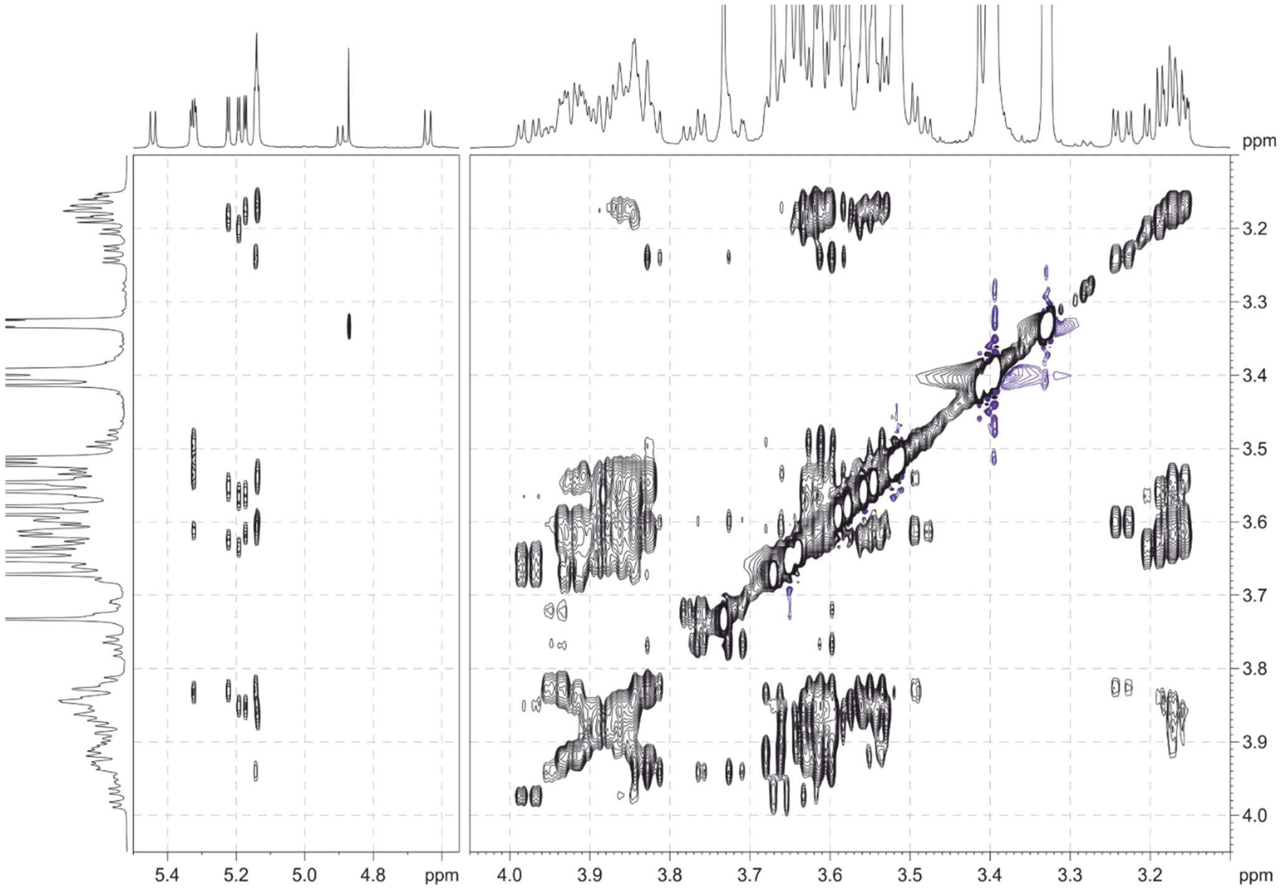

Supplementary Figure S9. 2D TOCSY NMR (600 MHz, MeOD) spectrum of compound **5** (3 mM).

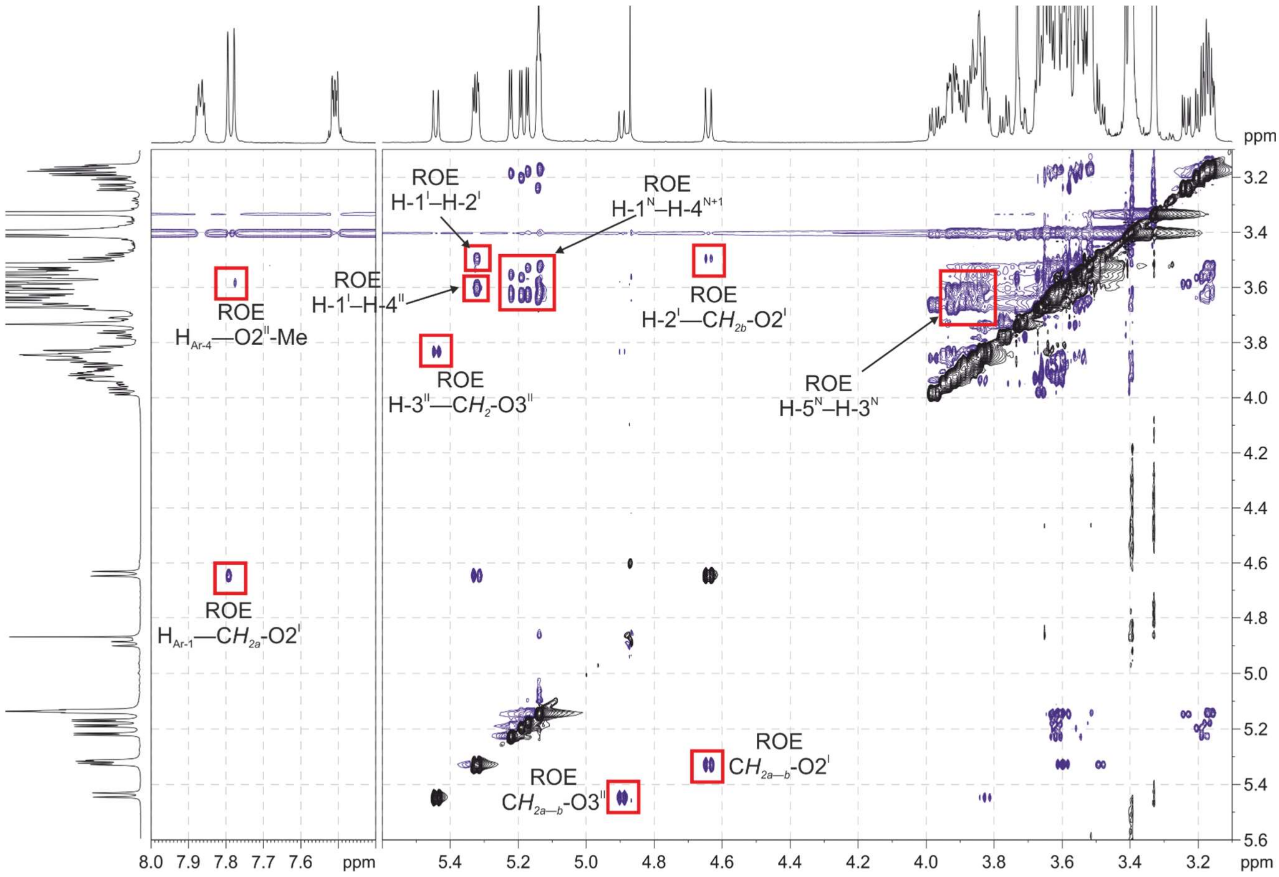

Supplementary Figure S10. 2D ROESY (600 MHz, MeOD) spectrum of compound **5** (3 mM).

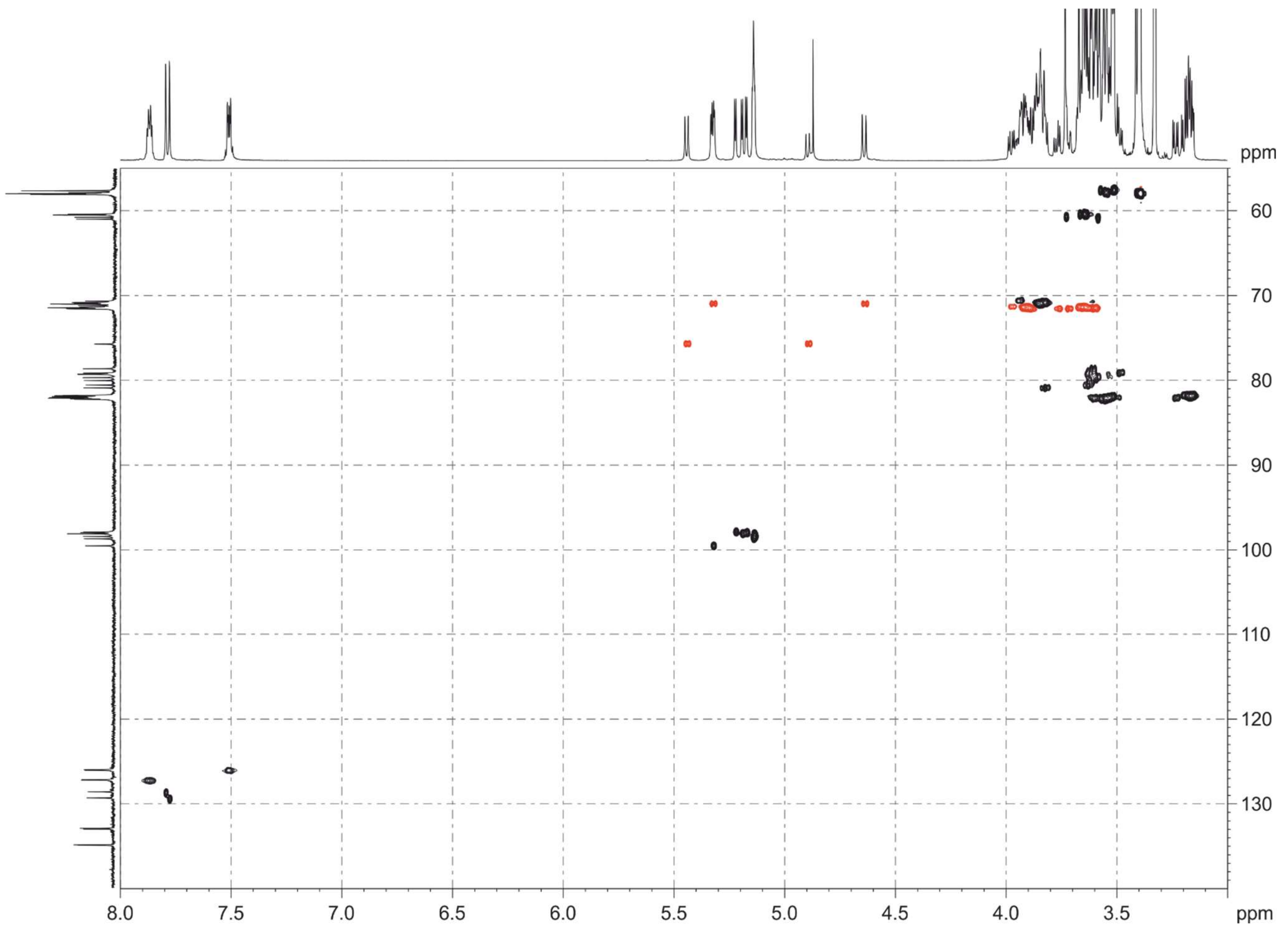

Supplementary Figure S11. HSQC (600 MHz, MeOD) spectrum of compound **5** (3 mm).

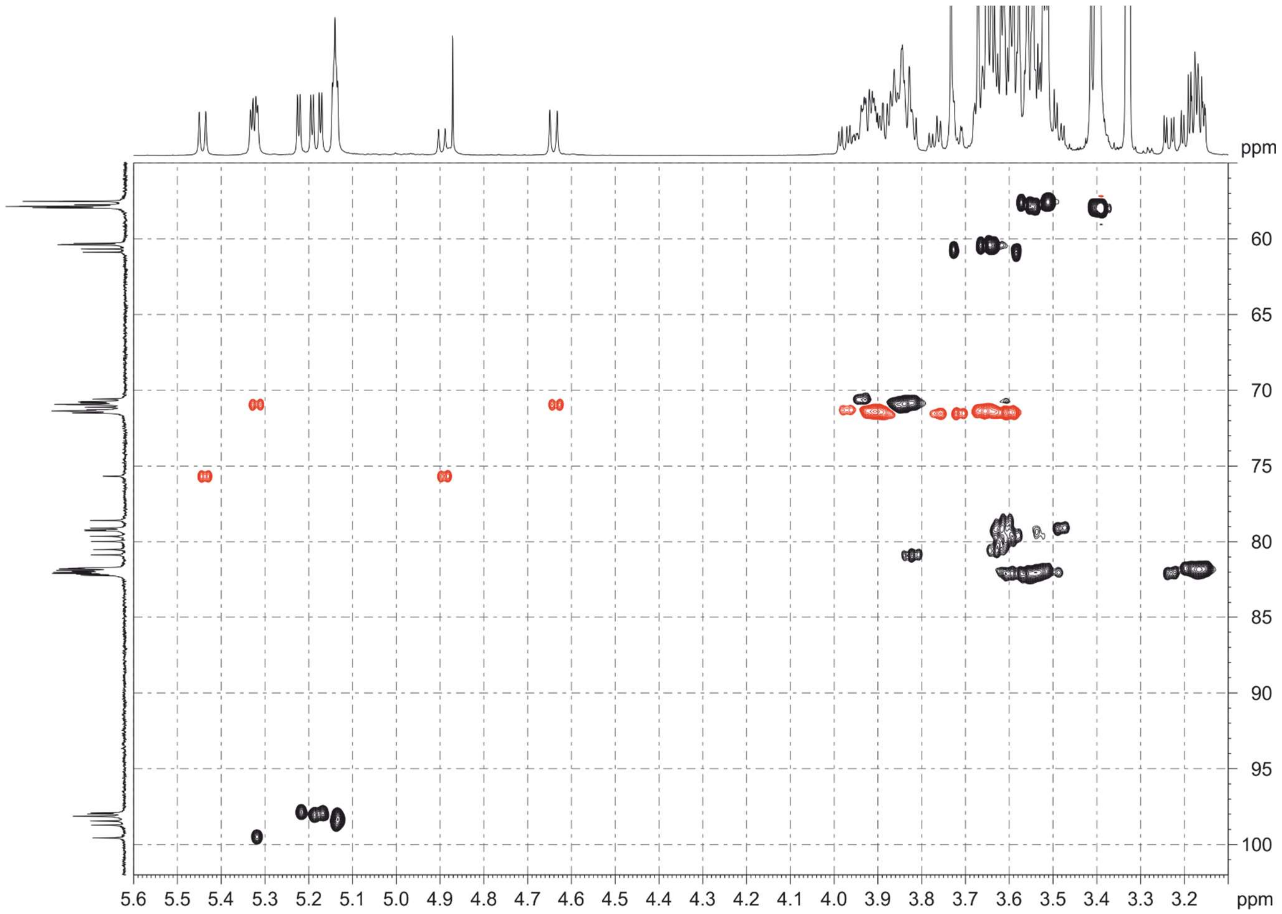

Supplementary Figure S12. Selected region of the HSQC (600 MHz, MeOD) spectrum of compound **5** (3 mM).

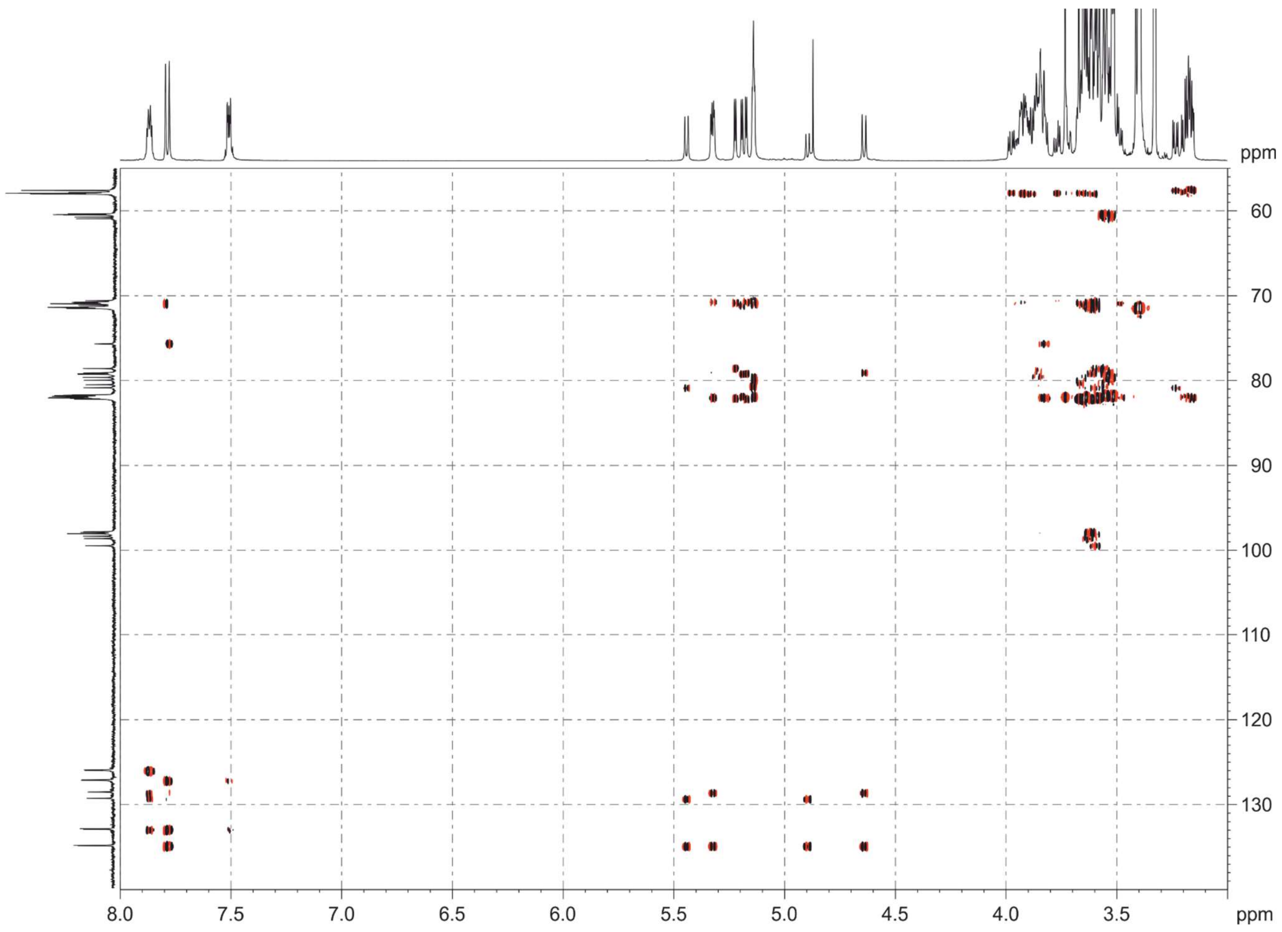

Supplementary Figure S13. HMBC (600 MHz, MeOD) spectrum of compound **5** (3 mm).

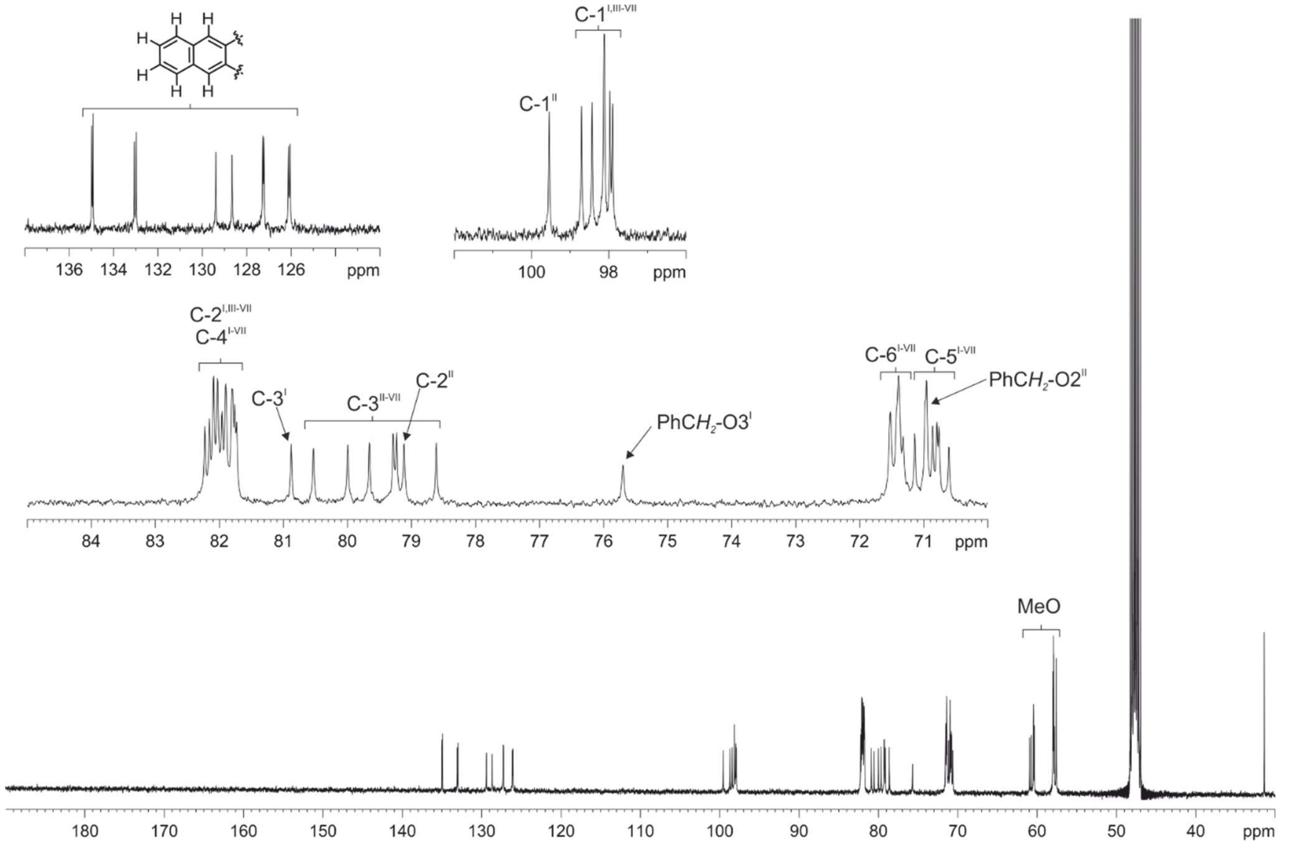

Supplementary Figure S14. ^{13}C NMR (100.6 MHz, MeOD) spectrum of compound **5** (3 mM).

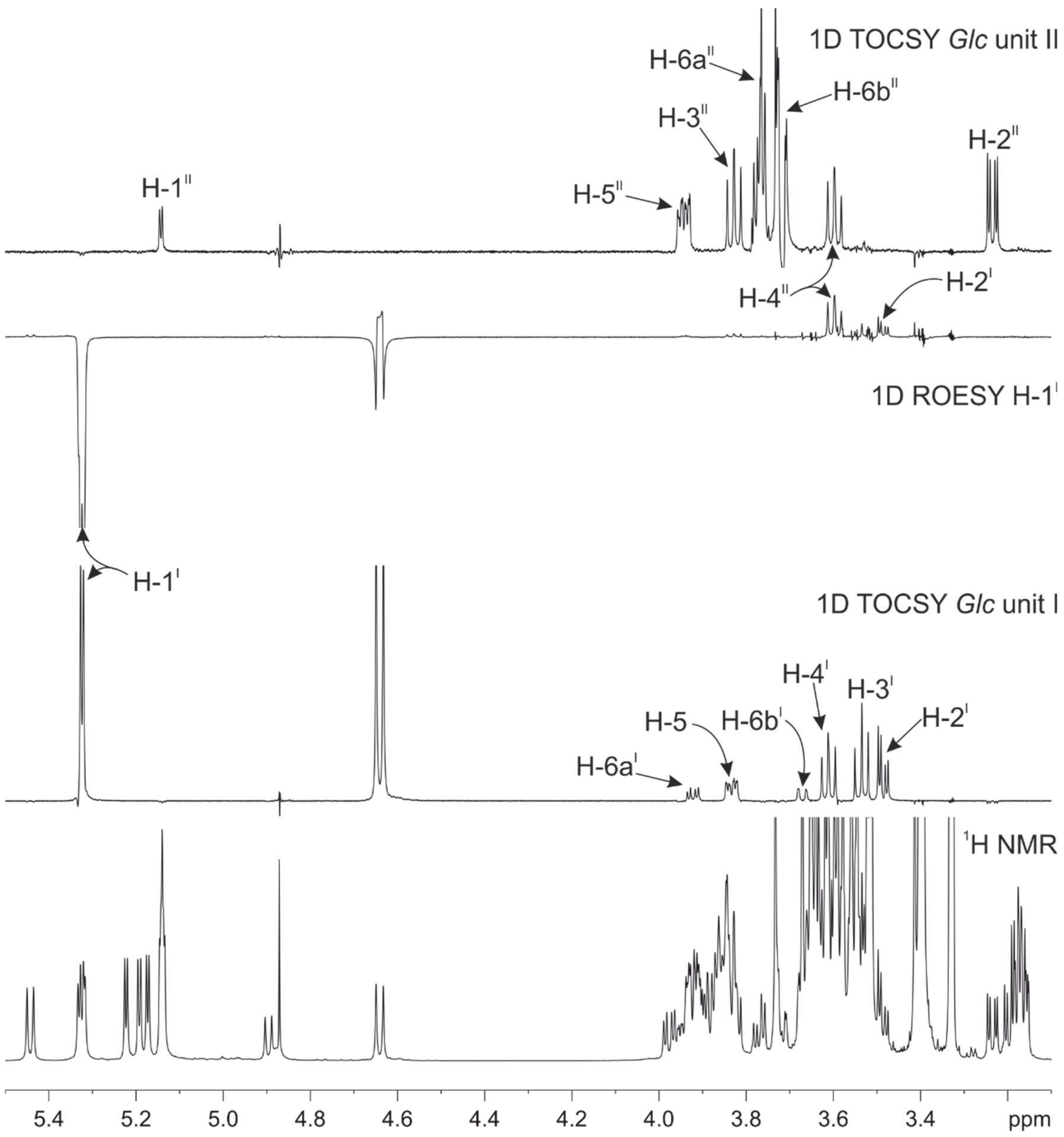

Supplementary Figure S15. Stacked ^{1}H, 1D TOCSY of glucose units I and II, and inter-glucoside I-II 1D ROESY (600 MHz, MeOD) spectra of compound **5** (3 mm).

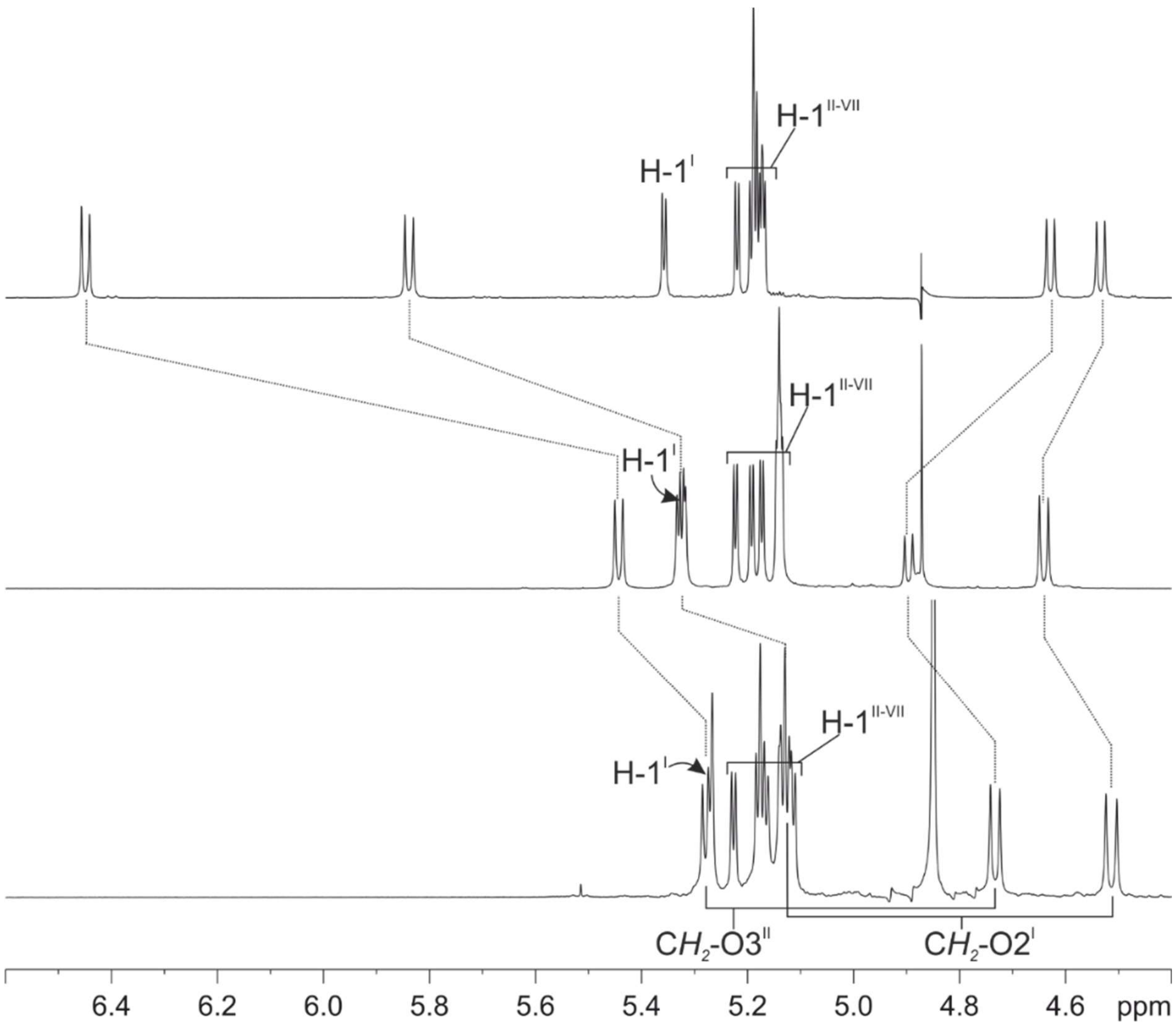

Supplementary Figure S16. Selected region (anomeric and benzylic proton resonances) of the ^{1}H NMR (600 MHz, MeOD) spectra of CD derivatives **4** (top) and **5** (middle) (3 mM) as compared to **2** (bottom).

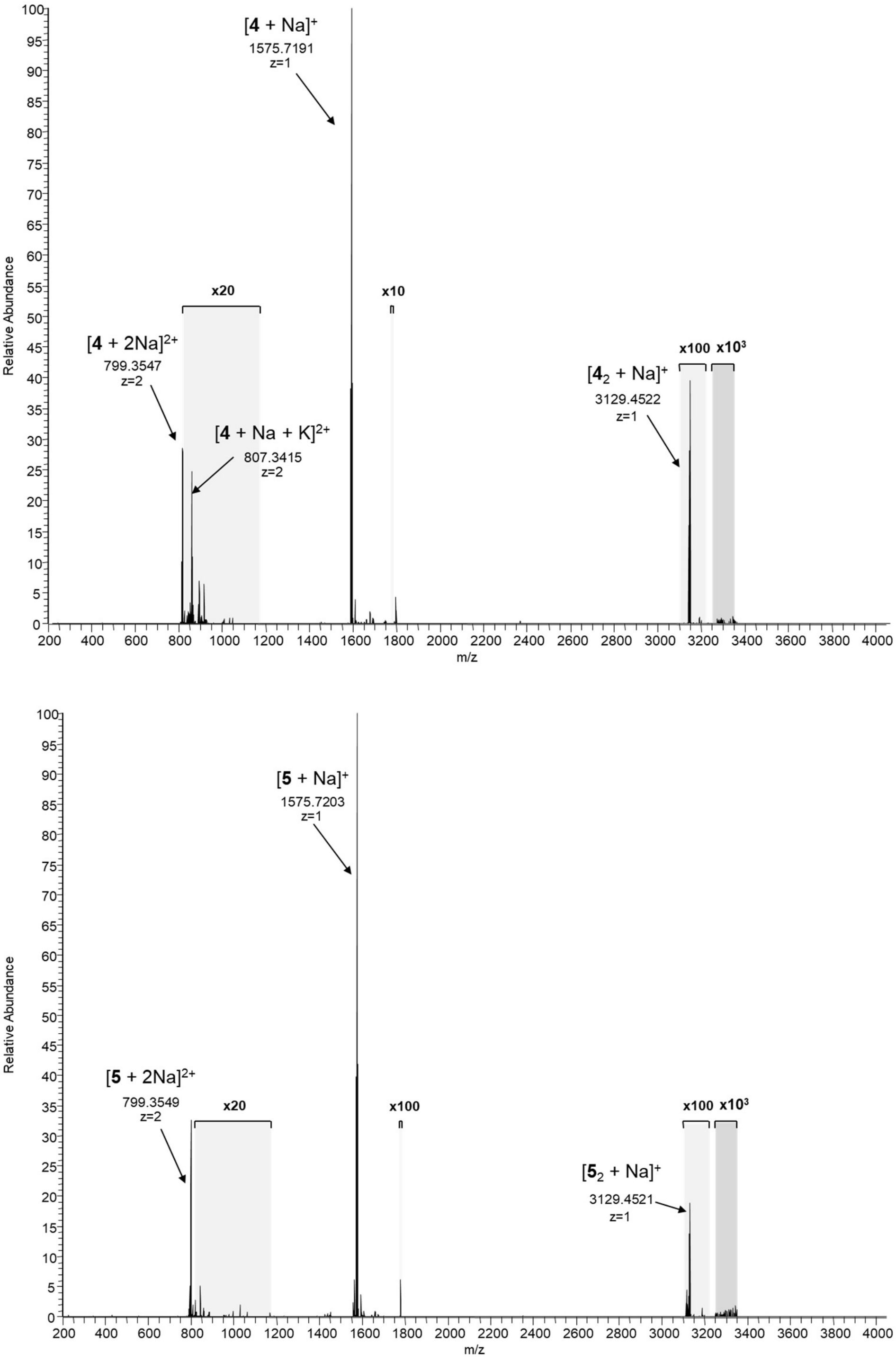

Supplementary Figure S17. HRESI mass spectra of **4** (top) and **5** (bottom) in 80:20 water-methanol. Together with the Na^+ salt of the monomeric CDs, the non-covalent dimers and doubly-charged monomers ([**4** + 2Na]$^{2+}$ and [**5** + 2Na]$^{2+}$) are highlighted.

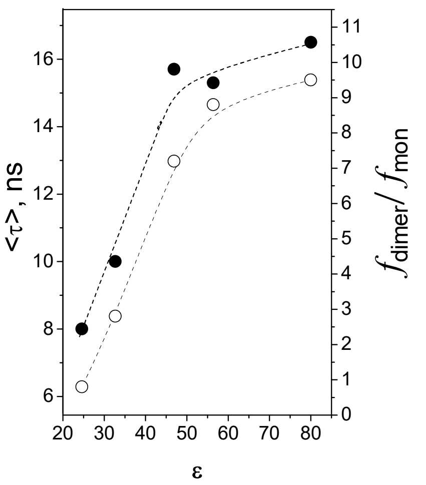

Supplementary Figure S18. Plots of the lifetime averages, <τ> (●) and the fractional dimer-to-monomer contribution ratios, f_{dimer}/f_{mon} (○) versus medium polarity (ε) from fluorescence decay measurements performed on solutions of **4** in water, methanol/water (50:50 and 30:70v/v), methanol and ethanol at 25 °C. [**4**] ≈ 1.5×10^{-4} M^{-1} in all experiments (dimer molar fraction ~0.86 in water at 25°C).

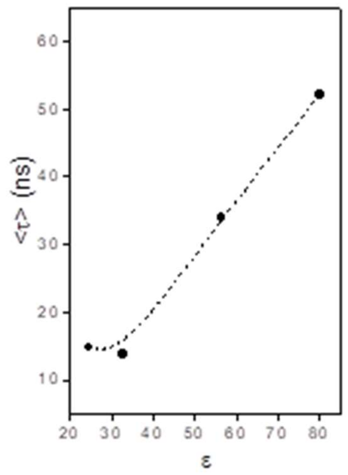

Supplementary Figure S19. Plots of lifetime averages, <τ> (●) versus medium polarity, (ε) from fluorescence decay measurements performed on solutions of **5** in water, methanol/water (50:50 v/v), methanol and ethanol at 25 ^oC. [**5**] ≈ 2.0×10^{-4} M^{-1} in all experiments (dimer molar fraction ~0.53 in water at 25 ^oC).

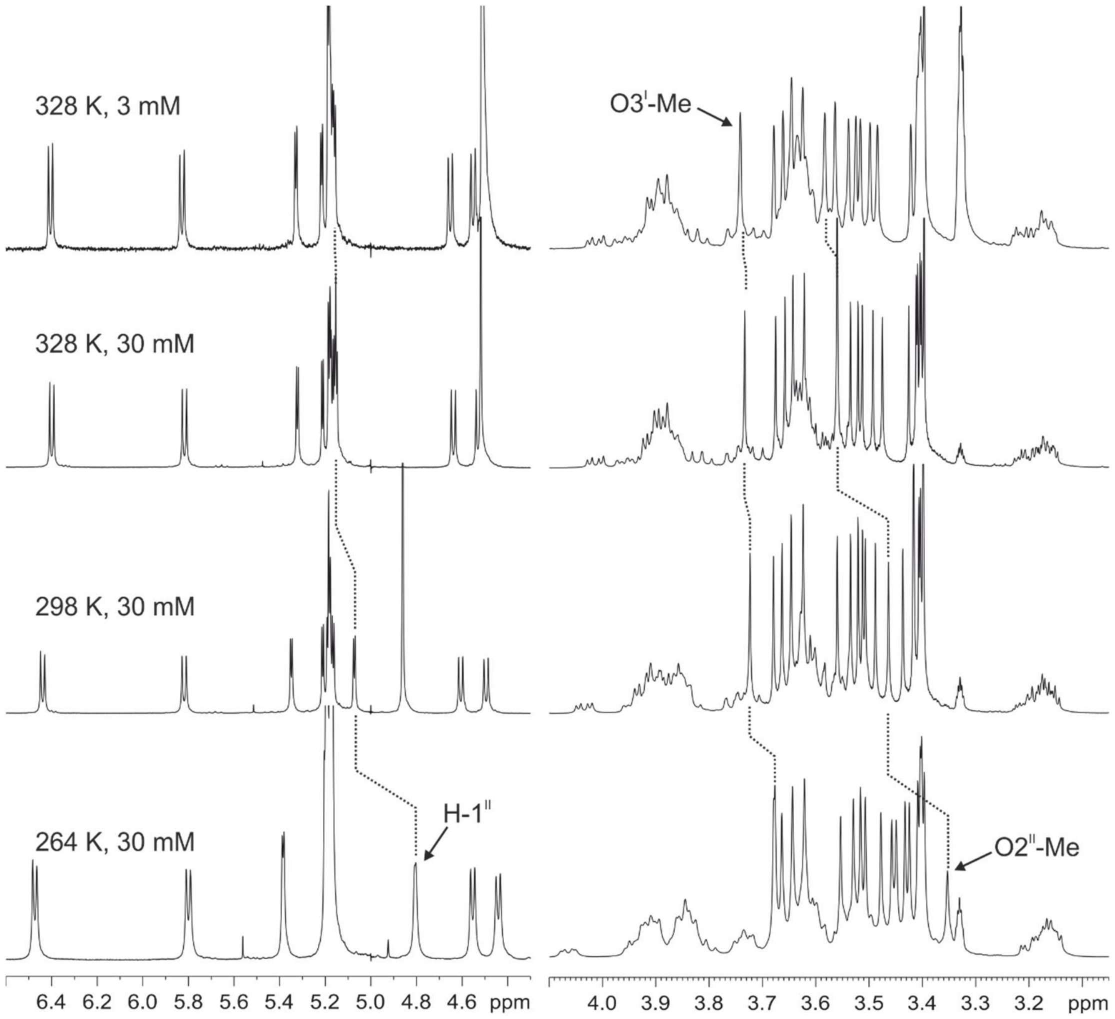

Supplementary Figure S20. Stacked ^{1}H NMR spectra (selected regions, 500 MHz, MeOD) of **4** at different concentrations and temperatures. High-field shifting resonances as a consequence of naphthalene segment shielding (H-1^{II} and O2^{II}-Me) are highlighted.

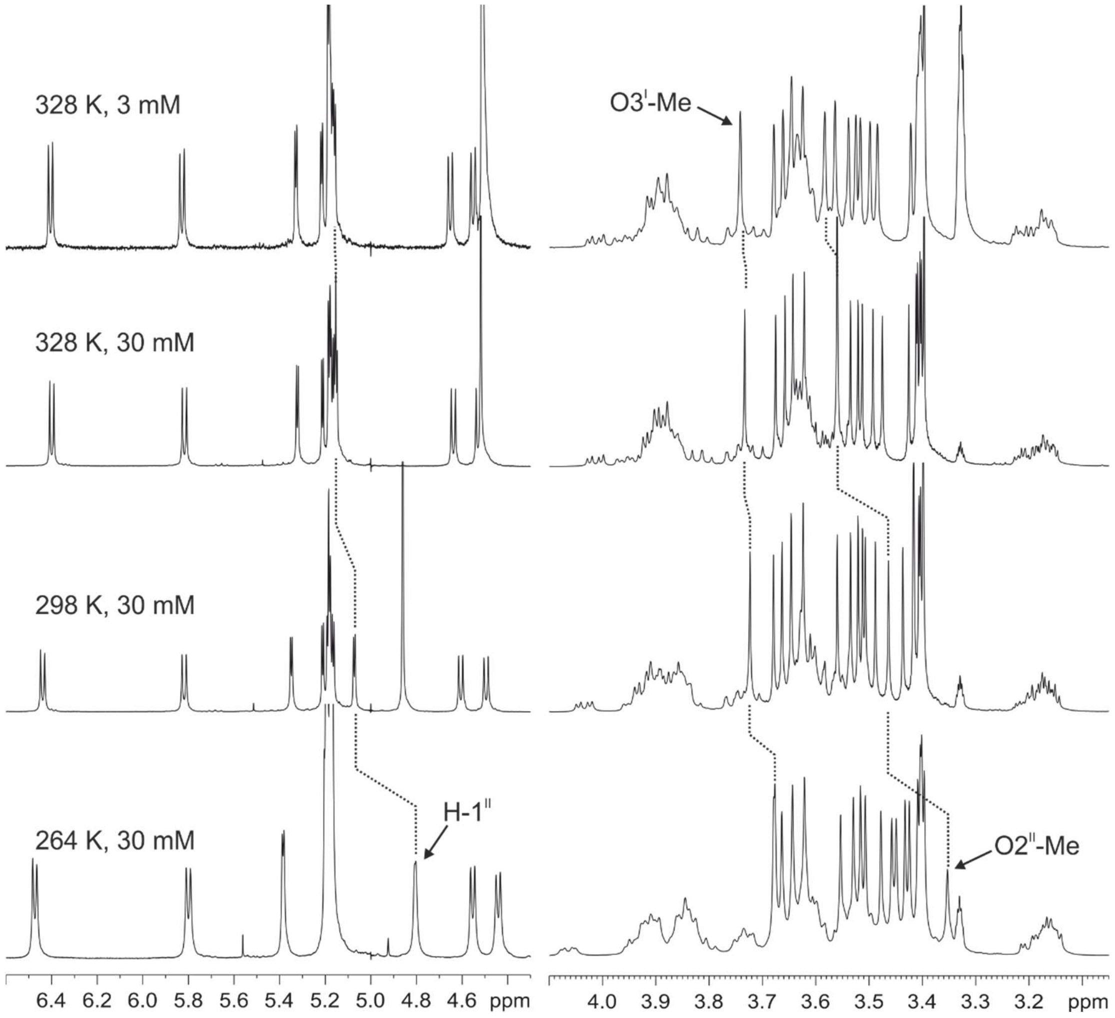

Supplementary Figure S21. Stacked ^{1}H NMR spectra (selected regions, 500 MHz, MeOD) of **5** at different concentrations and temperatures. High-field shifting resonances as a consequence of naphthalene segment shielding (H-1^{II} and O2^{II}-Me) are highlighted.

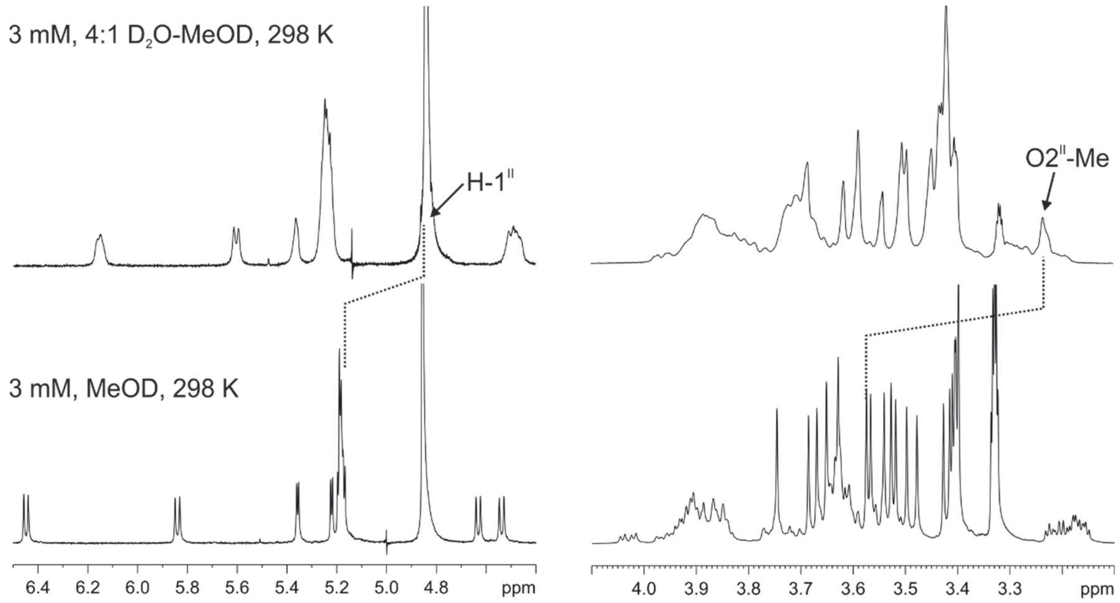

Supplementary Figure S22. Stacked ^{1}H NMR spectra (selected regions, 500 MHz, 298 K) of **4** (3 mm) in MeOD and 4:1 D_2O-MeOD. High-field shifting resonances as a consequence of naphthalene segment shielding (H-1^{II} and O2^{II}-Me) are highlighted.

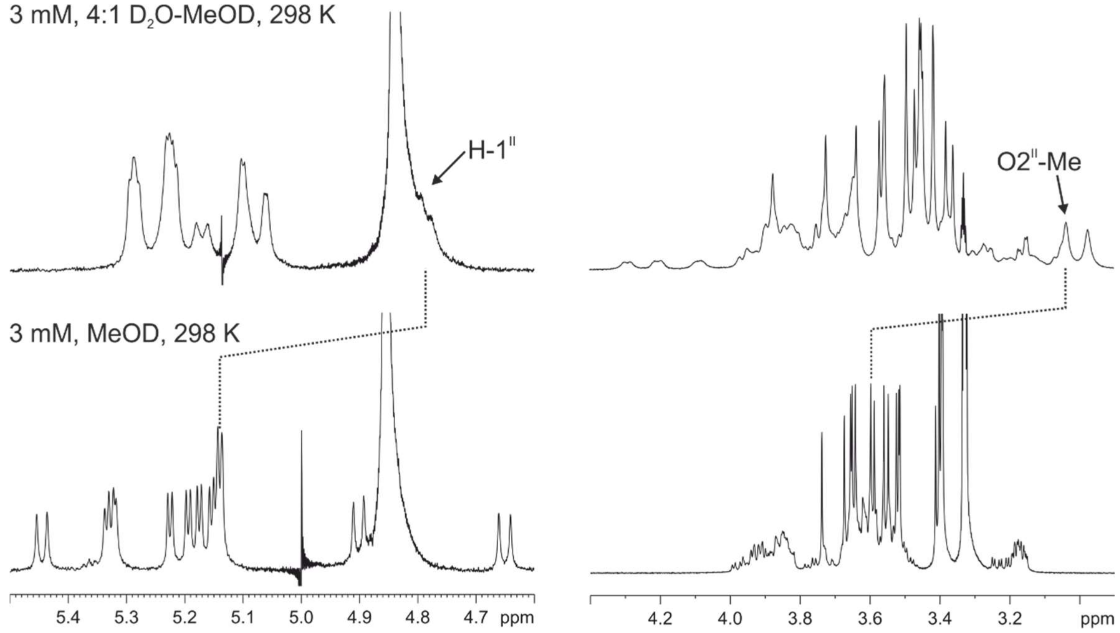

Supplementary Figure S23. Stacked ^{1}H NMR spectra (selected regions, 500 MHz, 298 K) of **5** (3 mm) in MeOD and 4:1 D_2O-MeOD. High-field shifting resonances as a consequence of naphthalene segment shielding (H-1^{II} and O2^{II}-Me) are highlighted.

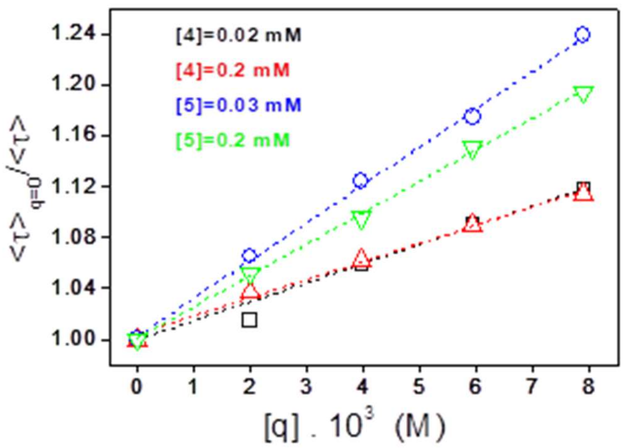

Supplementary Figure S24. Stern-Volmer plots obtained from fluorescence lifetime of **4** and **5** in water solutions at different quencher concentrations. 1.0-cm optical path quartz cells were used.

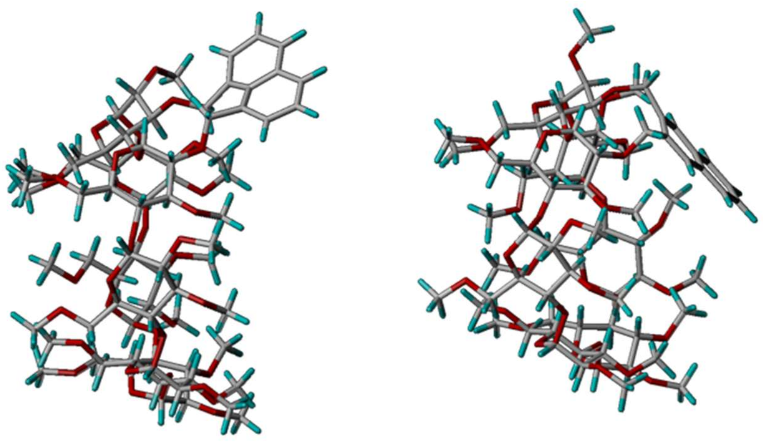

4 **5**

Supplementary Figure S25. Energy-minimized open (or fully-open) and half-open structures for CDs **4** and **5**, respectively. The most stable starting conformations for the naphthyl appended group were obtained by rotation around the C(2)-O-CH_2-C^{ar}(1) and C(3')-O-CH_2-C^{ar}(8) (or C(2)-O-CH_2-C^{ar}(2) and C('3)-O-CH_2-C^{ar}(3)) ether bonds tethering the naphthyl group to βCD for **4** (for **5**) respectively, similarly to that described previously. These rotations were defined by four named χ torsional angles.

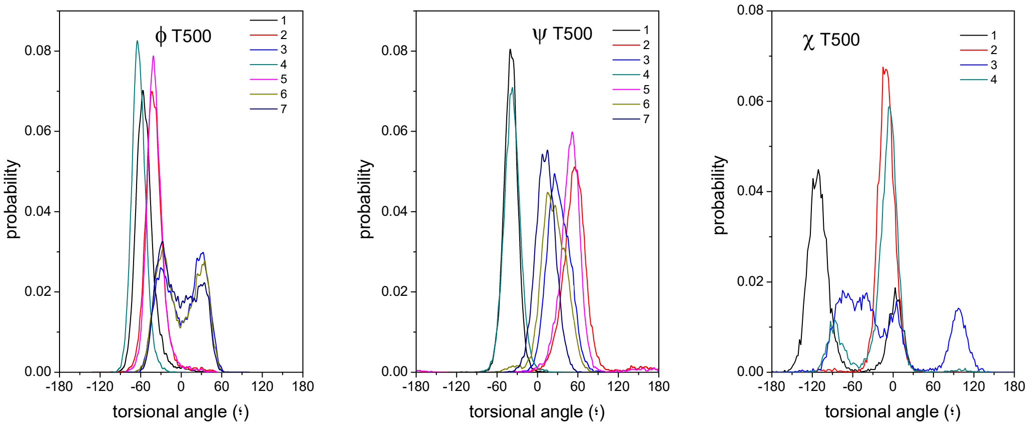

Supplementary Figure S26. Probability distribution of the torsional ϕ, ψ and χ angles obtained from the analysis of the 2-ns MD trajectory in the vacuum at 500 K for **4**. Dihedral ϕ and ψ angles were defined by $C(4^{i})\ldots C(1^{i})$-O-$C(4^{i+1})$ and $C(1^{i})$-O-$C(4^{i+1})\ldots C(1^{i+1})$ (where … denotes the 1^{i}-4^{i} or 4^{i+1}-1^{i+1} virtual bonds). Dihedral χ angles were defined by: $C(1^{i})$-$C(2^{i})$-O-CH_2; $C(2^{i})$-O-CH_2 -$C^{ar}(1)$; $C(4^{i+1})$-$C(3^{i+1})$-O-CH_2; $C(3^{i+1})$-O-CH_2 -$C^{ar}(8)$ for **4**.

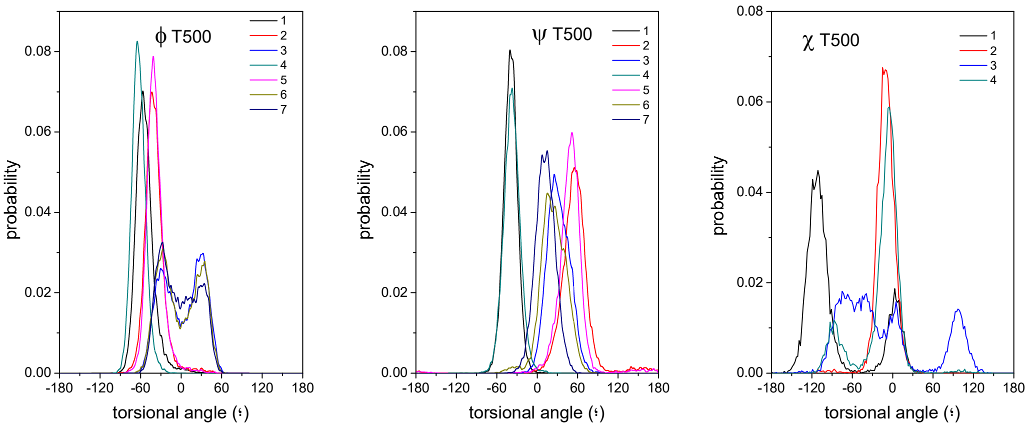

Supplementary Figure S27. Probability distribution of the torsional ϕ, ψ and χ angles obtained from the analysis of the 2-ns MD trajectory in the vacuum at 500 K for **5**. Dihedral ϕ and ψ angles were defined by $C(4^{i})\ldots C(1^{i})$-O-$C(4^{i+1})$ and $C(1^{i})$-O-$C(4^{i+1})\ldots C(1^{i+1})$ (where … denotes the 1^{i}-4^{i} or 4^{i+1}-1^{i+1} virtual bonds). Dihedral χ angles were defined by: $C(1^{i})$-$C(2^{i})$-O-CH_2; $C(2^{i})$-O-CH_2 -$C^{ar}(2)$; $C(4^{i+1})$-$C(3^{i+1})$-O-CH_2; $C(3^{i+1})$-O-CH_2 -$C^{ar}(3)$ for **5**.

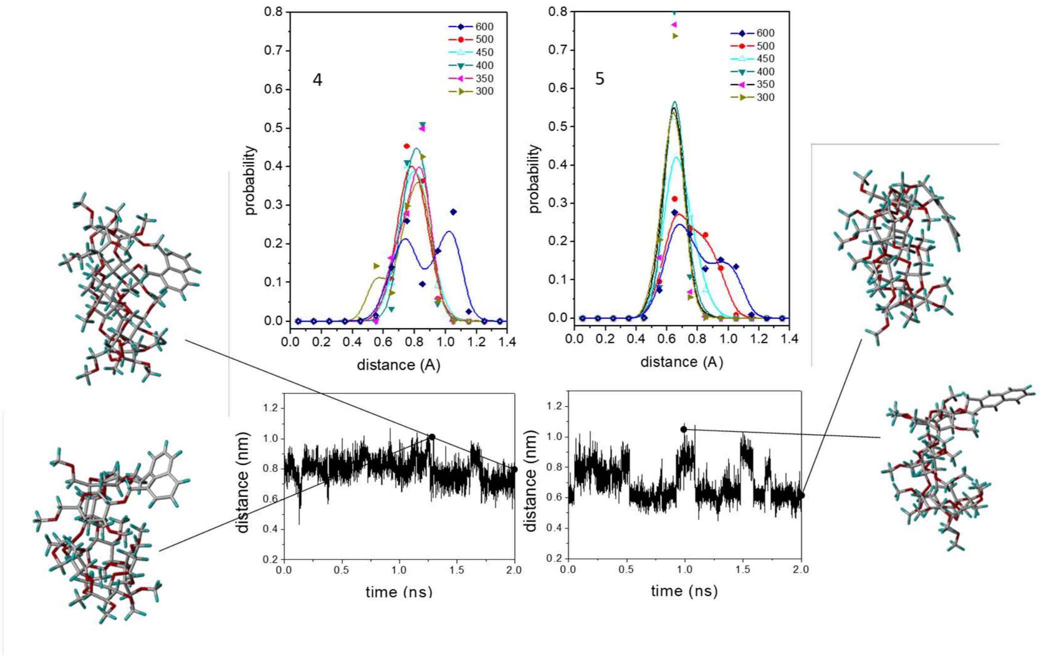

Supplementary Figure S28. Probability distribution of the distance between the center of mass of macroring oxygen atoms and the naphthalene group obtained from the analysis of the 2-ns MD trajectory in the vacuum at different temperatures for **4** (left) and **5** (right). Histories of the distances described in the previous sentence at 450 K are shown below. Some of the structures are superimposed.

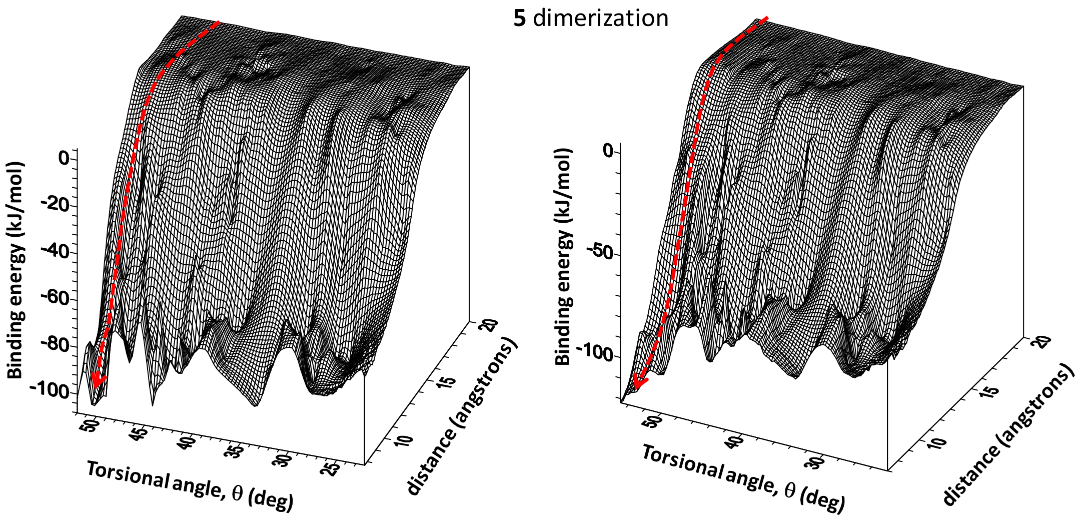

Supplementary Figure S29. Critical analysis of the structures generated by scanning the θ[O(4)-o-o'-O(4')] dihedral angle in the –180 to +180º range (initially at 30º intervals and later 10º) and the y coordinate (o-o' distance between centers of both CDs) from 2 to 0.7 nm (0.05 nm intervals) in the presence of water for **5**.

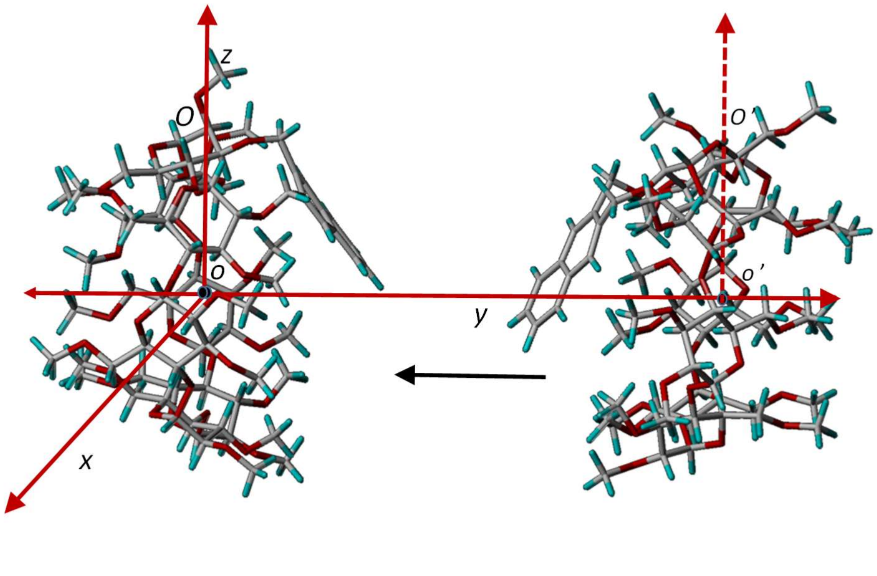

Supplementary Figure S30. Coordinate systems used to emulate the dimerization processes for the head-to-head (HH) approaching of **5** to another **5** molecule.

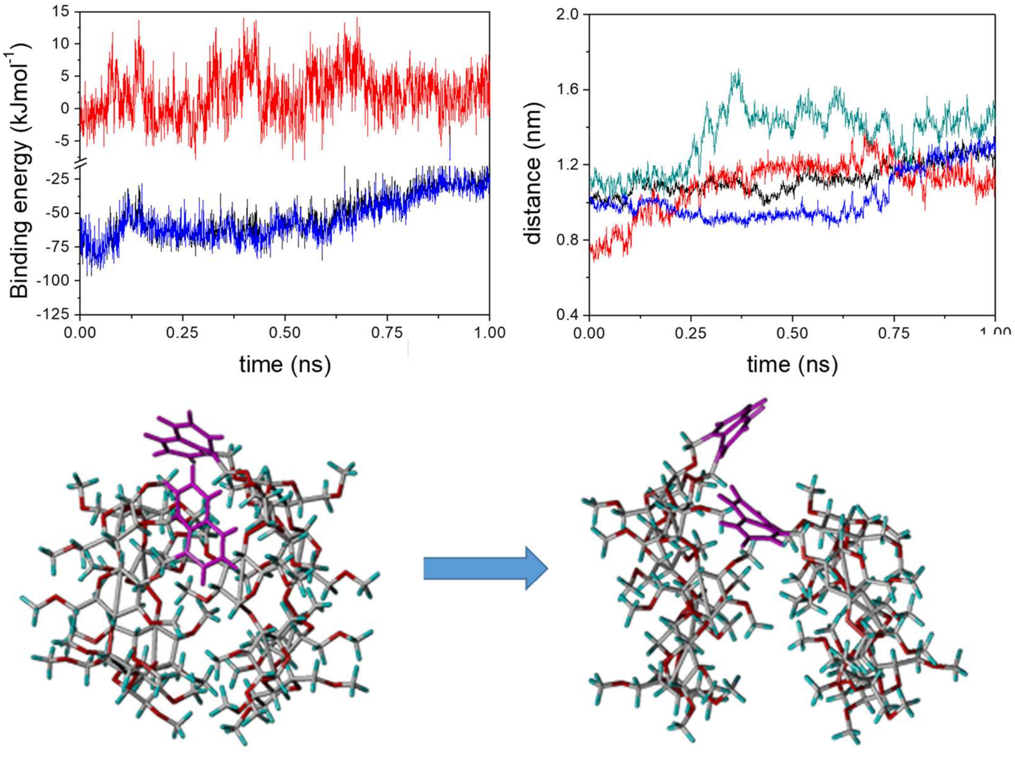

Supplementary Figure S31. Top-left panel depicts the histories for the HH-type **4-**dimer total binding of energy (black) and the electrostatic (red) and van der Waals (blue) contributions, obtained from analysis of the 1-ns MD trajectories on the **4** MBE dimer structure in water (depicted in Figure 10). Top-right panel represents the histories of distances between the center of mass of both macrorings (black), naphthyl chromophores (red), CD1 and naphthyl2 (green), and CD2 and naphthyl1 (blue).

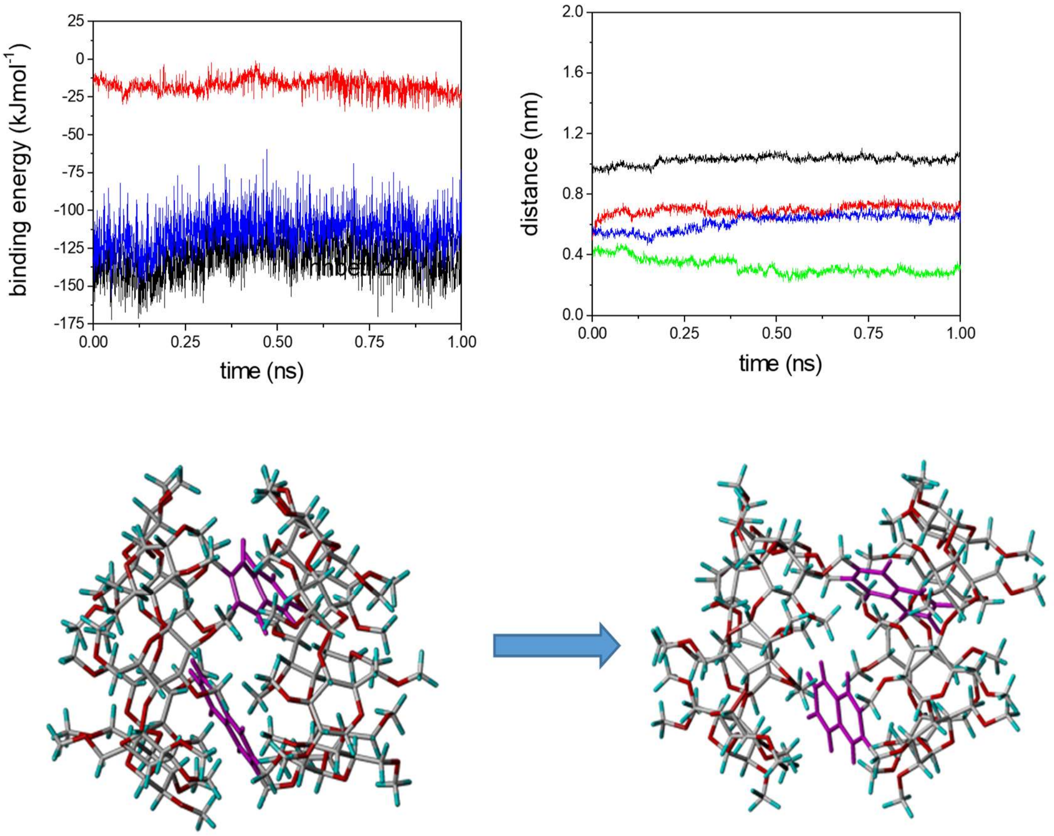

Supplementary Figure S32. Top-left panel depicts the histories for the HH-type **5-**dimer total binding of energy (black) and the electrostatic (red) and van der Waals (blue) contributions, obtained from analysis of the 1-ns MD trajectories on the **5** MBE dimer structure in water (depicted in Figure 11). Top-right panel represents the histories of distances between the center of mass of both macrorings (black), naphthyl chromophores (red), CD1 and naphthyl2 (green), and CD2 and naphthyl1 (blue).

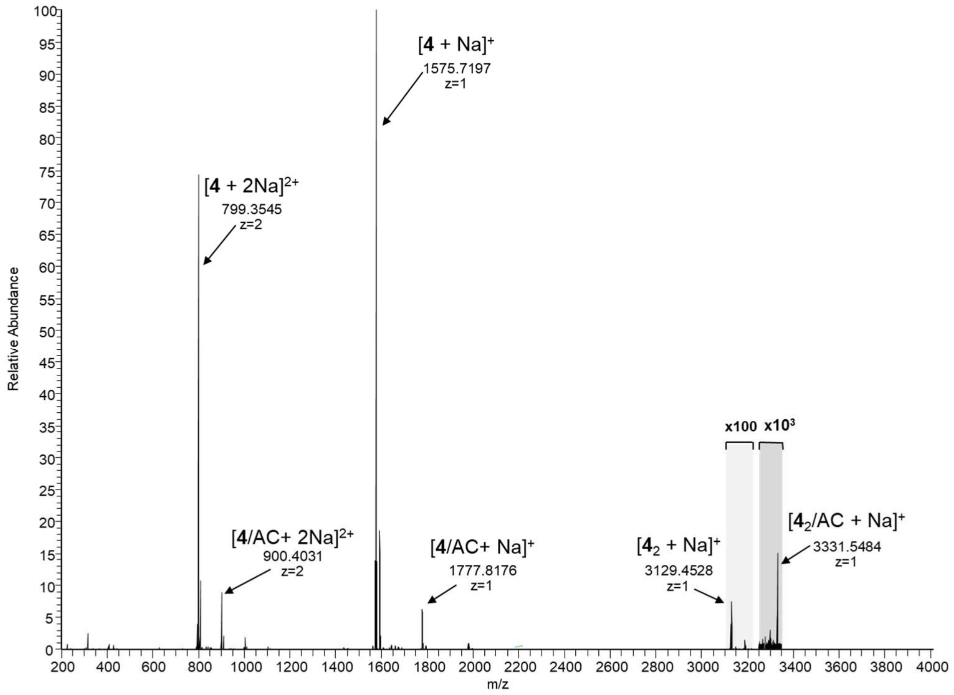

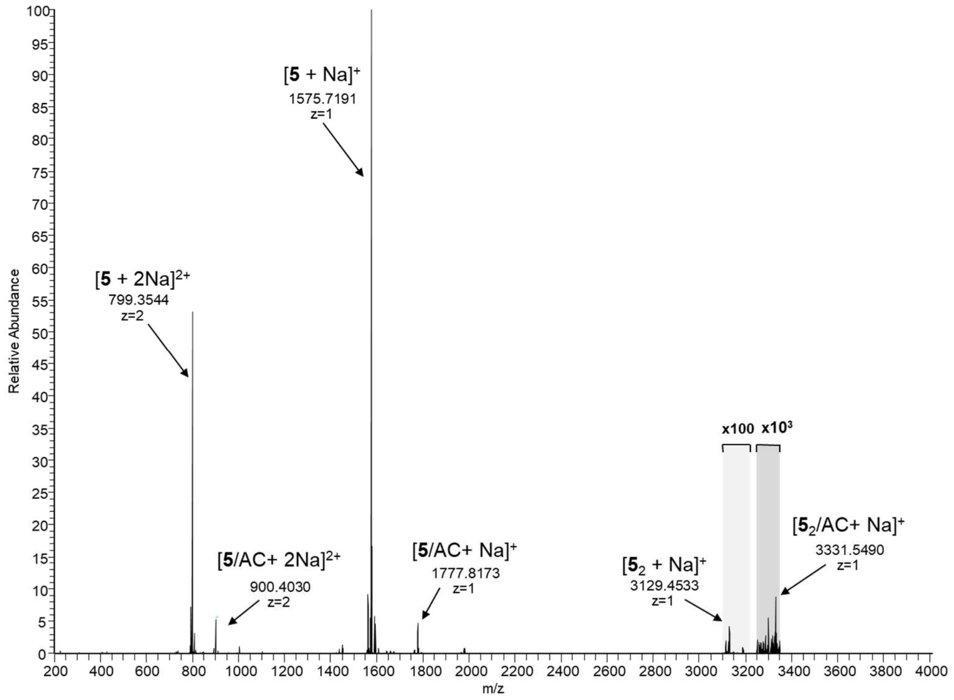

Supplementary Figure S33. HRESI mass spectra of **4**:AC (top) and **5**:AC (bottom) mixtures (1:4 molar ratio) in 80% water solution, highlighting the relative abundance of CD:AC, CD_2 and $\mathbf{CD_2}$:AC species.

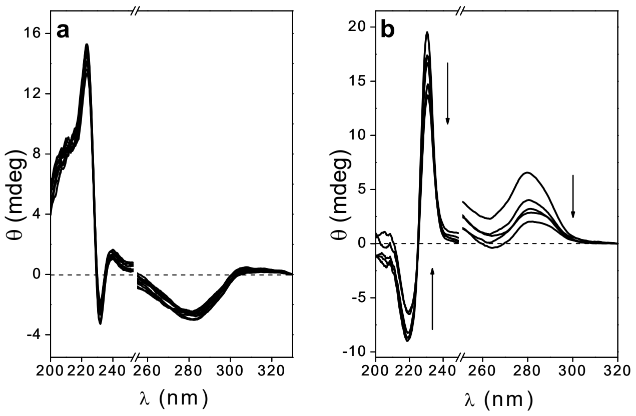

Supplementary Figure S34. Circular dichroism spectra for **4** (a, 1.36×10^{-4} m) and **5** (b, 1.47×10^{-4} m) in water at increasing AC concentration. [AC]/[**4**] = 0, 0.22, 0.44, 0.66, 0.88, 1.10, 1.31, 1.51, 1.75 and 1.97 and [AC]/[**5**] = 0, 0.25, 0.50, 0.75, 1.00, 1.25, 1.50, 1.75, 2.00 2.24 and 2.49 molar ratios.

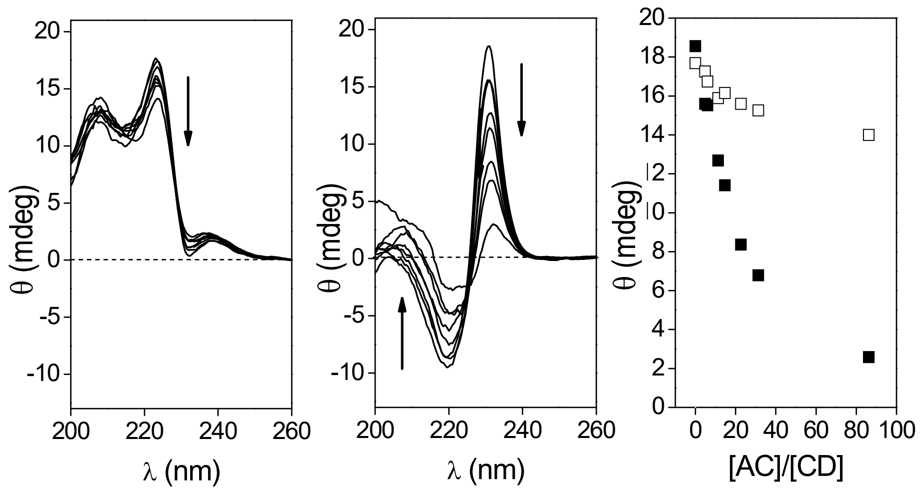

Supplementary Figure S35. Circular dichroism spectra for **4** (left) and **5** (middle) in water at different AC/CD molar ratios: [AC]/[**4**]= 0, 5, 13, 19, 29, 43, 64 and 106 and [AC]/[**5**]= 0, 5, 6, 11, 15, 23, 31 and 86. Ellipticity vs molar ratios measured at λ = 223 nm (231 nm) for **4** (□) and **5** (■) are represented in right panel. The cell path was 1.0 cm and the temperature 25 °C.

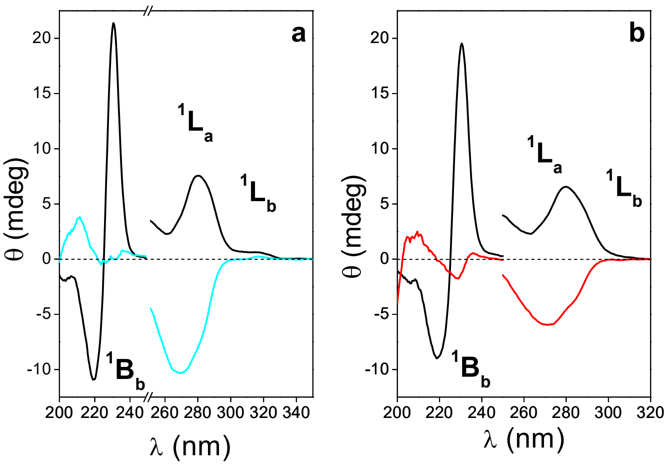

Supplementary Figure S36. Comparative circular dichroism spectra of (a) solutions of **5** alone in water (black line) and in propanol (blue line), and (b) in the absence (black lines) and in the presence (red line) of a large excess of AC in water. Concentrations of **5** were to 0.20 and 0.15 mm for (a) and (b), respectively.

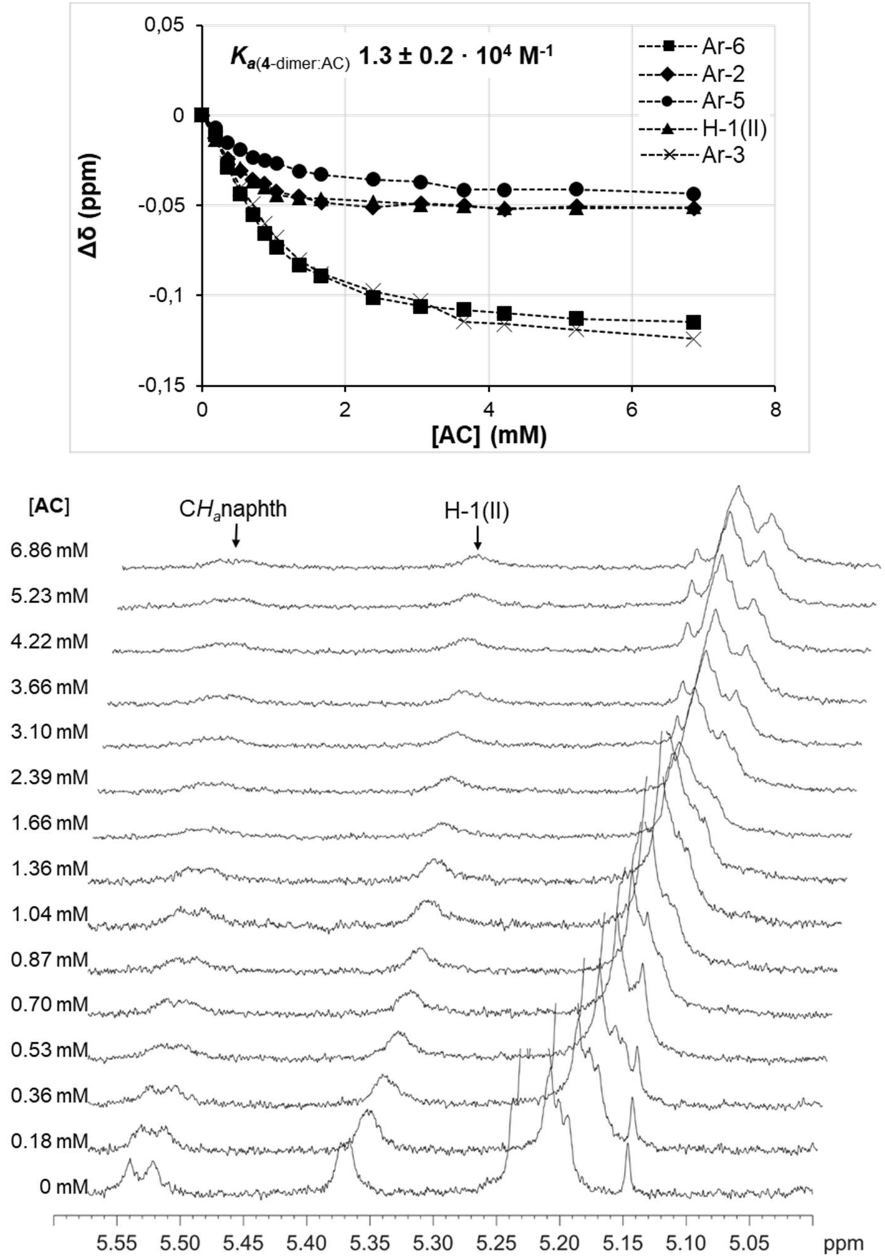

Supplementary Figure S37. Top panel: Binding isotherms obtained from the titration of CD derivative **4** (0.53 mM) with AC in phosphate-buffered D_2O (pD 7.4, 0.1 M) at 298 K. The resonances of some aromatic protons and H-1 are plotted. The curves fit to a 1:1 stoichiometry binding model between **4**-dimer and AC (i.e., a 2:1 stoichiometry based on **4**-monomer), yielding a K_a value of $1.3 \pm 0.2 \cdot 10^4$ M^{-1} for the equilibrium **4**-dimer + AC ⇆ **4**-dimer:AC. Bottom panel: Stacked ^{1}H NMR spectra (selected region) of the above **4**:AC titration experiment with indication of some of the resonances used in the K_a fitting (C$H_a$naphth(I) and H-1(II)) and the AC concentration at each experiment.

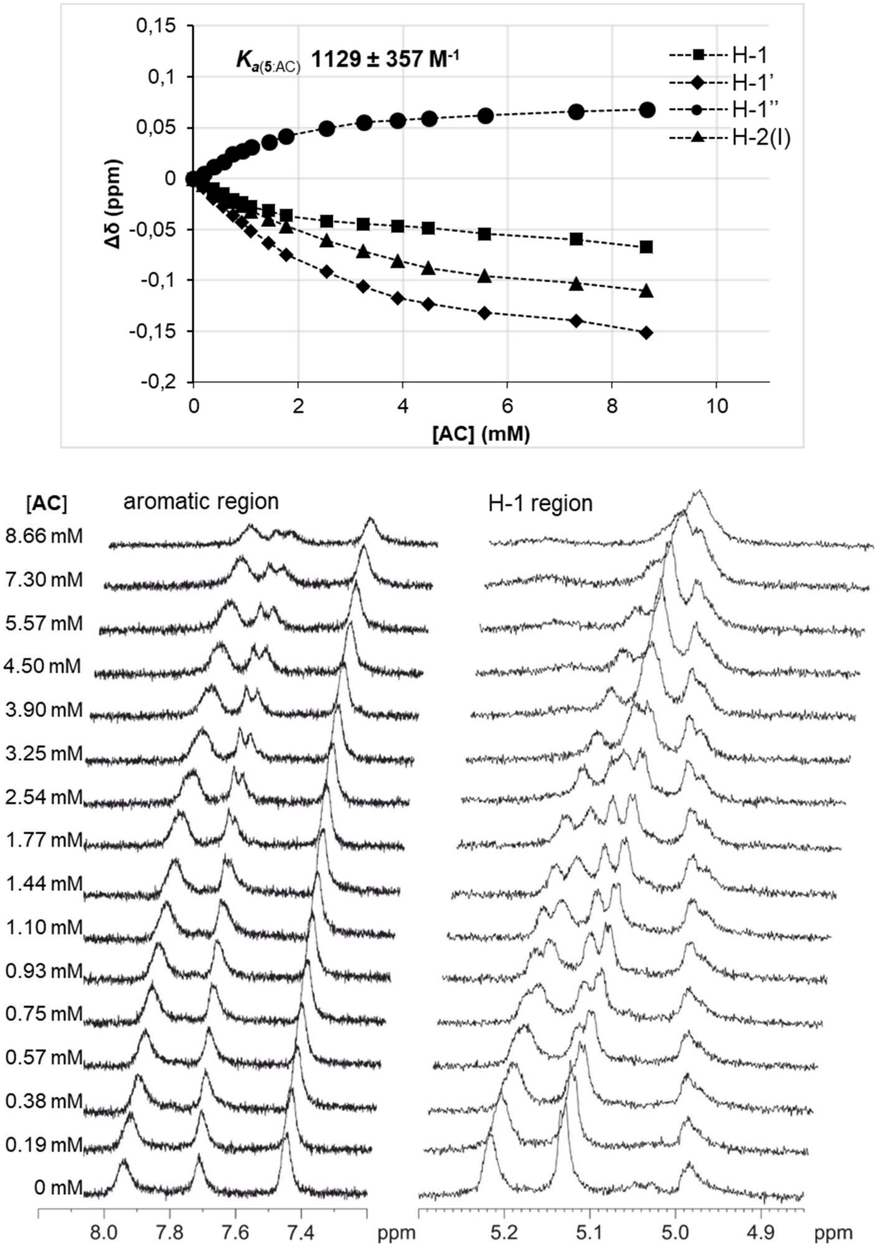

Supplementary Figure S38. Top panel: Binding isotherms obtained from the titration of CD derivative **5** (0.94 mM) with AC in phosphate-buffered D_2O (pD 7.4, 0.1 M) at 298 K. The resonances of aromatic protons and H-1 are plotted. The curves fit to a 1:1 stoichiometry binding model, yielding a K_a value of 1129 ± 357 M^{-1}. Bottom panel: Stacked ^{1}H NMR spectra (selected regions) of the above **5**:AC titration experiment with indication of some of the resonances used in the K_a fitting (C$H_a$naphth(I) and H-1) and the AC concentration at each experiment.

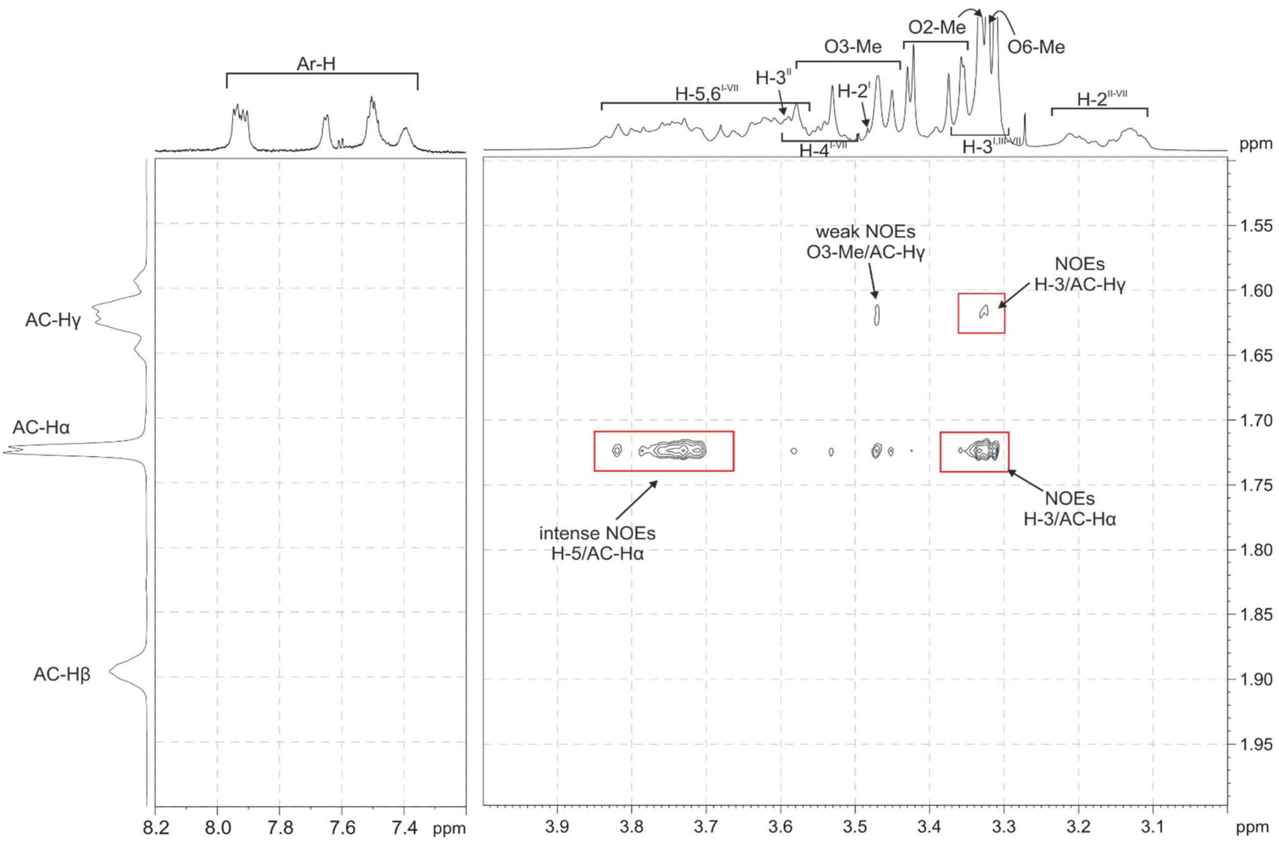

Supplementary Figure S39. Selected regions of the 2D NOESY (600 MHz, D_2O, phosphate buffer 0.1 M, pD 7.4) spectrum of **4**:AC ([**4**] 0.53 mM, [AC] 18 mM) at 0.8 s mixing time. Most relevant cross peaks determining host:guest relative orientation are highlighted.

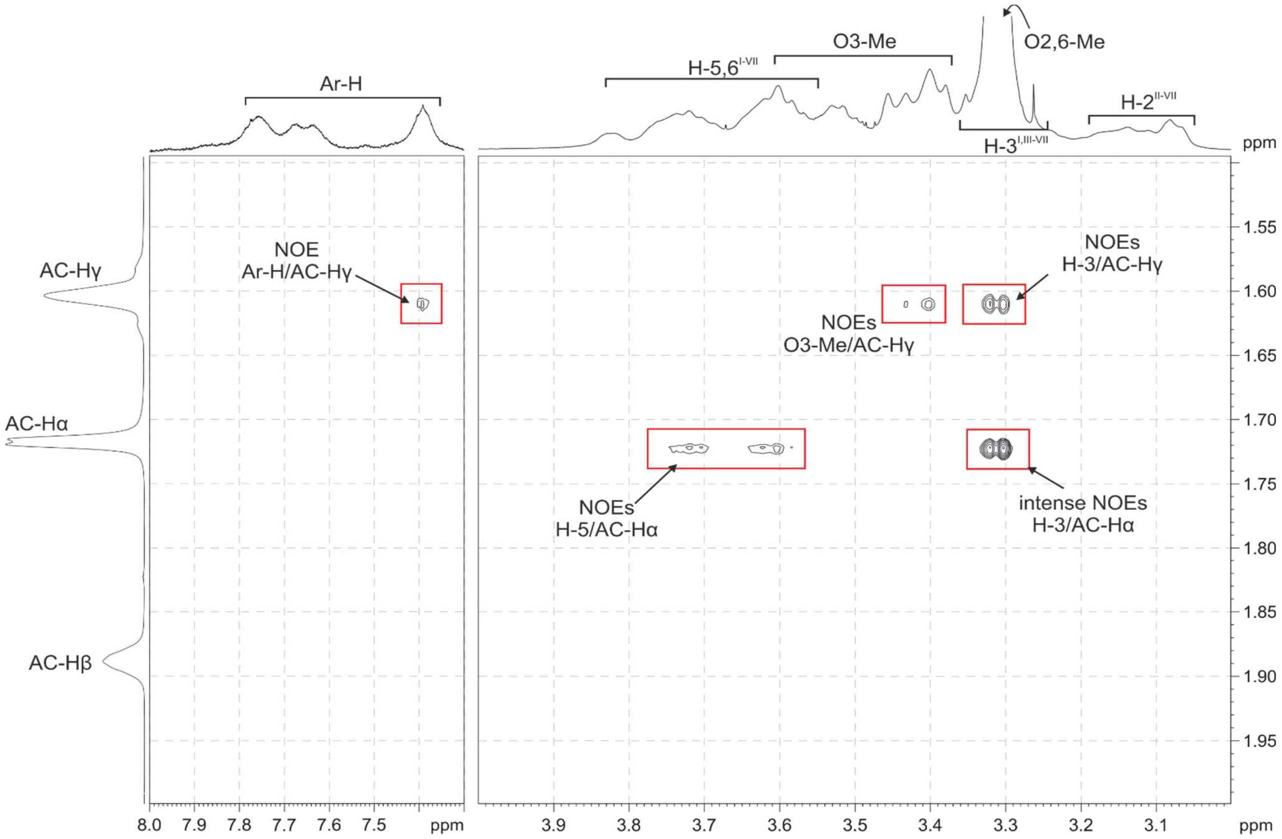

Supplementary Figure S40. Selected regions of the 2D NOESY (600 MHz, D_2O, phosphate buffer 0.1 M, pD 7.4) spectrum of **5**:AC ([**5**] 0.94 mM, [AC] 19.5 mM) at 0.8 s mixing time. Most relevant cross peaks determining host:guest relative orientation are highlighted.

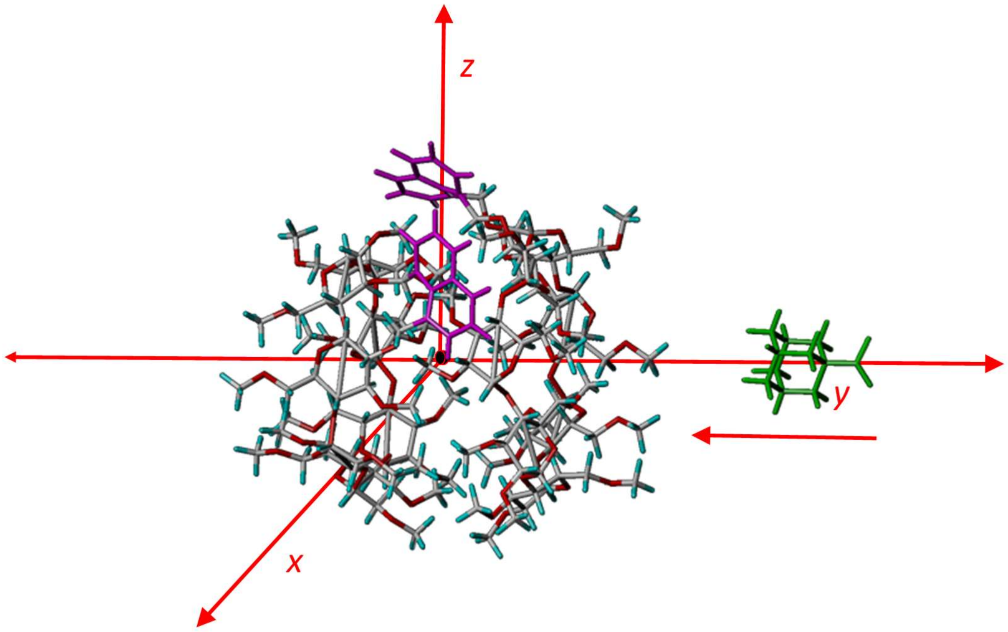

Supplementary Figure S41. Coordinate systems used to emulate the complexation process of HH **4**-dimer and AC to form the **4**-dimer:AC complex.
